# *mTrop1/Epcam* Knockout Mice Develop Congenital Tufting Enteropathy through Dysregulation of Intestinal E-cadherin/β-catenin

**DOI:** 10.1371/journal.pone.0049302

**Published:** 2012-11-28

**Authors:** Emanuela Guerra, Rossano Lattanzio, Rossana La Sorda, Francesca Dini, Gian Mario Tiboni, Mauro Piantelli, Saverio Alberti

**Affiliations:** 1 Unit of Cancer Pathology, CeSI, “University G. d'Annunzio” Foundation, Chieti, Italy; 2 Department of Medicine and Research on Aging, School of Medicine, University “G. d'Annunzio”, Chieti, Italy; 3 Department of Biomedical Sciences, University “G. d'Annunzio”, Chieti, Italy; 4 Department of Neuroscience and Imaging, BAMS, University “G. d'Annunzio”, Chieti, Italy; Montana State University, United States of America

## Abstract

Congenital tufting enteropathy (CTE) is a life-threatening hereditary disease that is characterized by enteric mucosa tufting degeneration and early onset, severe diarrhea. Loss-of-function mutations of the human *EPCAM* gene (*TROP1*, *TACSTD1*) have been indicated as the cause of CTE. However, loss of *mTrop1/Epcam* in mice appeared to lead to death *in utero*, due to placental malformation. This and indications of residual Trop-1/EpCAM expression in cases of CTE cast doubt on the role of *mTrop1/Epcam* in this disease. The aim of this study was to determine the role of *TROP1*/*EPCAM* in CTE and to generate an animal model of this disease for molecular investigation and therapy development. Using a rigorous gene-trapping approach, we obtained *mTrop1/Epcam* -null (knockout) mice. These were born alive, but failed to thrive, and died soon after birth because of hemorrhagic diarrhea. The intestine from the *mTrop1/Epcam* knockout mice showed intestinal tufts, villous atrophy and colon crypt hyperplasia, as in human CTE. No structural defects were detected in other organs. These results are consistent with *TROP1*/*EPCAM* loss being the cause of CTE, thus providing a viable animal model for this disease, and a benchmark for its pathogenetic course. In the affected enteric mucosa, E-cadherin and β-catenin were shown to be dysregulated, leading to disorganized transition from crypts to villi, with progressive loss of membrane localization and increasing intracellular accumulation, thus unraveling an essential role for Trop-1/EpCAM in the maintenance of intestinal architecture and functionality.

Supporting information is available for this article.

## Introduction

EpCAM, also known as Trop-1, from the trophoblast cells in which it was originally defined [1], is a transmembrane glycoprotein [2], [3], [4] that shares unique structural features with its paralog Trop-2 [5], [6]. Both Trop-1 and Trop-2 regulate cell-cell adhesion [7], [8] and cell growth [4], [9], [10]. Trop-1 is expressed by embryonic stem (ES) cells, where it contributes to the maintenance of pluripotency [11]. In the developing embryo, Trop-1 expression is detected in oral and nasal cavities, ear, eye, respiratory tract, gut mucosa, kidney, liver, pancreas, skin, gonads, and placental trophoblast [1], [12], [13]. Trop-1 expression in tissue primordia is developmentally regulated and it was proposed to have a morphoregulatory role [14]. In the adult organism, Trop-1 is a marker of adult epithelial and hematopoietic progenitors, and of proliferating epithelia [4], [12].

Inactivating germ-line mutations of the human *EPCAM*/*TROP1/TACSTD1* gene [15] have been associated with congenital tufting enteropathy (CTE) [16], a life-threatening intestinal dysplasia that manifests from birth. CTE is characterized by gross lesions in the intestinal epithelium, with villous atrophy, crypt hyperplasia and focal crowding of enterocytes (tufts) [17]. Affected individuals show abnormal expression of α2β1 integrin, desmoglein, laminin and heparan sulfate proteoglycan, and ultrastructural changes to cell desmosomes in the intestinal epithelium [18], [19], which indicate the loss of epithelial barrier function. Several *TROP1* homozygous or compound heterozygous mutations have been described in CTE to date, i.e., base substitutions in the donor or acceptor splice sites of exon 4, with in-frame exon skipping, and nonsense mutations or base insertions in exons 3, 5 and 6, which lead to premature truncation of the protein in the extracellular domain [16], [20], [21], [22], [23]. CTE-associated mutations have been linked to either decreased or absent Trop-1 expression [16], [22], [23].

Loss-of-function animal models have been used to tackle the *in-vivo* role of Trop-1. In zebrafish embryos, *TROP1* inactivation via retroviral insertion or somatic knockdown by antisense oligonucleotides showed that Trop-1 is required for epithelial morphogenesis and integrity, for otolith formation in the inner ear [24], and for lateral line formation by specialized cells that differentiate from migrating primordia [25]. It should be noted that in zebrafish there is only one *TACSTD*-like gene [25], thus preventing compensatory effects/functional substitution by the *TROP2* paralog.

Recently, a role for the murine EpCAM/mTrop-1 protein in intercellular adhesion and cell motility and migration was shown in a mouse conditional knockout (KO) with *Epcam*/*mTrop1-*specific inactivation in epithelial Langerhans cells [26]. On the other hand, constitutive *mTrop1* ablation [13] has been suggested to lead to embryonic lethality by day of gestation (E) 12.5, due to placental defects. This cast doubt on *TROP1* mutations as a single-gene-inactivation cause of CTE, potentially implicating other, nearby gene defects as obligate and/or modulatory determinants for disease appearance. However, *mTrop1* KO validation in this murine model was performed through surrogate markers (β-galactosidase-neomycin phosphotransferase fusion (βGEO) genotyping and β-galactosidase (β-gal) expression/activity) [13], thus preventing the identification of possible off-target effects by the gene-trapping procedure.

Hence, we used rigorous gene-replacement and gene-trapping approaches, and obtained a gene-trapped KO mouse that was devoid of a functional mTrop-1 protein. The *mTrop1*-null embryos showed no *in-utero* morphological defects, and were born alive. On the other hand, *mTrop1*-null pups showed rapidly progressing intestinal epithelium dysplasia, with focal cell crowding and tufting, which closely paralleled that seen in CTE. This led to severe hemorragic enteropathy, which caused impaired development and death within a few days from birth. No structural defects were seen in other organs. Taken together, our findings are consistent with Trop-1 loss being a single-gene cause of CTE. Molecular analysis of the affected epithelia showed disruption of cryptae-to-villi transition and progressive intracellular accumulation of the adherens-junction organizer E-cadherin and its interactor β-catenin, revealing a direct role of Trop-1 in the maintenance of intestinal architecture and functionality, through regulation of E-cadherin/β-catenin expression and cellular localization.

## Materials and Methods

### Nomenclature

*EPCAM/TROP1* indicates the human gene, *Epcam*/*mTrop1* indicates the murine gene; EpCAM/Trop-1 is the human protein product, mTrop-1 is the murine protein [4], [38]. The synonym *TROP1*, as was defined for the first time in trophoblast cells [1] and as the gene of origin of the *TROP* family [2], [5], [6] is used in this report.

The exon numbering in mouse and man differs, as an additional 5′-untranslated exon has been described in the mouse (NM_008532.2), for a total of 10 exons, *versus* 9 in man, e.g., human exon 4 corresponds to murine exon 5.

### Plasmids

The pGT1TMPFS vector was used to generate gene-trapped clones from ES cells [39]. It contains 1721 bp of the mouse *Engrailed 2* (*En2*) intronic sequence that ends with a splice-acceptor AG dinucleotide upstream of a promoterless β-GEO open reading frame (ORF) in each of the three reading frames. Upon insertion of this cassette within an intron the β-GEO ORF is spliced to the preceding exon, leading to a chimeric translated product.

### Cells

The feeder-independent E14Tg2A.4 ES cell line obtained from 129/Ola mice was used for gene-trapping. The RST412 and RST413 *mTrop1-*gene-trapped clones (International Gene Trap Consortium, IGTC) (www.genetrap.org/cgi-bin/annotation.py?gene_key=1992) were grown as described [39]. Both gene-trap clones are available to the scientific community.

### Antibodies

The G8.8 rat anti-mTrop-1 mAb [4] and the secondary Alexa Fluor-conjugated 488-goat anti-rat (GAR) IgG (Invitrogen) were used for immunofluorescence microscopy and flow cytometry. The A-11132 rabbit anti-β-galactosidase polyclonal antibody (Molecular Probes, Carlsbad, CA) [40], the 24E10 rabbit anti-E-cadherin mAb (Cell Signaling Inc., Beverly, MA) and the E-5 mouse anti-β-catenin mAb (Santa Cruz Biotechnology, Santa Cruz, CA) were used for immunohistochemistry, as indicated.

### Animals

Procedures involving animals were conducted in compliance with institutional guidelines and with national (D.L. No. 116, G.U., Suppl. 40, Feb.18, 1992; circolare No. 8, G.U., July, 1994) and international laws and policies (UKCCCR Guidelines for the Welfare of Animals in Experimental Neoplasia; EEC Council Directive 86/609, OJ L 358. 1, Dec.12, 1987; Guide for the Care and Use of Laboratory Animals, United States National Research Council, 1996). Experiments on animals were approved by the Interuniversity Animal Research Ethics Committee (CEISA) of Chieti–Pescara and Teramo Universities. Animals were anesthetized with ketamine/xylazine before any invasive procedures. Euthanasia was performed by CO_2_ inhalation followed by cervical dislocation (adult mice) or decapitation (newborn mice). All efforts were made to minimize suffering of the animals. The *mTrop1* KO mouse is available to the scientific community.

### Genotyping

Mouse genotyping was performed on genomic DNA extracted from tail biopsies or embryonic tissues (Supporting Materials and Methods). Marker-specific genotyping was performed by multiplex polymerase chain reaction (PCR) with primer pairs KO-*neo*-F2/KO-*neo*-R2/(β-GEO cassette) and m*β-globin*-F1/m*β-globin*-R1 (murine *β-globin* as housekeeping) (Table S1) and the following cycle: 95°C 5 min; 95°C 30 s, 59°C 30 s, 72°C 30 s, repeated 35 times; 72°C 5 min. Specific genotyping for the *mTrop1*-β-GEO fusion was performed by multiplex PCR with primers mT1EX2-F2, β-gal-Baygen-R3 (gene-trapped mTrop1–allele) and mT1Int3-R1 (WT *mTrop1* allele) (Table S1) and the following cycle: 95°C 5 min; 95°C 30 s, 64°C 30 s, 72°C 2 min, repeated 35 times; 72°C 5 min. The KapaBlood PCR Kit B (Cambridge, MA) was used. The mice studied had been backcrossed to B6 for at least 6 generations, resulting in a >99% B6 genetic background.

### Morphology and histopathology analyses

Timed matings between fertile males and spontaneously cycling females were set up to obtain embryos at defined developmental stages. Pregnant female mice were sacrificed between E9.5 and E10.5 (vaginal plug = E0.5). The uterus was removed and quickly rinsed in cold phosphate-buffered saline (PBS). Individual embryos were isolated either within their intact decidual swelling or as dissected from the surrounding maternal tissues. The morphology of the freshly dissected embryos was analyzed under a stereo-microscope (G.M.T.). Embryos were then embedded in optimal cutting temperature (OCT) compound and snap frozen in liquid N_2_, for subsequent histopathology and molecular analyses. Newborn mice were sacrificed at different times after birth. Internal organs were excised, formalin-fixed, and paraffin-embedded. The gastrointestinal tract (stomach, small intestine and colon) was quickly removed as a whole, rinsed in PBS and either formalin-fixed and paraffin-embedded or frozen (snap freezing in liquid nitrogen for nucleic acid extraction, or OCT embedding for cryostatic microtome sectioning). Five-µm organ sections were stained with hematoxylin and eosin (H&E) following standard procedures, and examined (M.P. and R.L.). Mouse tail tips were processed for DNA extraction and genotyping.

### Immunohistochemistry

Five-micrometer sections of formalin-fixed and paraffin-embedded tissues from WT and KO mice were stained using the indicated antibodies. Antigen retrieval was performed by microwave treatment at 750 W for 10 min in 10 mM sodium citrate buffer (pH 6.0). After blocking endogenous mouse immunoglobulins using the Rodent Block kit (Biocare Medical, Concord, CA), sections were incubated overnight with the anti-β-catenin (1∶30 dilution) and anti-E-cadherin (1∶200 dilution) primary antibodies. The anti-mouse and the anti-rabbit EnVision kits (Dako, Glostrup, Denmark) were used for signal amplification, as appropriate. In control sections the specific primary antibody was replaced with isotype-matched immunoglobulins (Dako).

### Flow cytometry

Cell staining and flow cytometry analyses (FACScalibur, FACScan, Becton Dickinson, Sunnyvale, CA) were performed as previously described [38].

### Statistical analyses

The χ^2^ test was used to compare genotype ratios. Kaplan–Meier plots [41], [42] were used to compute survival in specified cohorts. The log-rank test was used to assess equality of survival curves (SPSS package, version 15.0; SPSS, Chicago, IL). Two-way analysis of variance (ANOVA) was used for comparison of newborn growth curves [43].

## Results and Discussion

### *mTrop1* KO mice are born alive and develop CTE

We used both gene-replacement and gene-trapping approaches, and corresponding validation procedures, to obtain a KO mouse devoid of functional *mTrop1*. For inactivation of *mTrop1* through gene replacement, we used homology-guided recombination in mouse ES cells (Text S1; Figs. S1, S2). We succeeded in obtaining ES cells with one inactivated *mTrop1* allele. However, these failed to colonize blastocysts efficiently, and no chimeric mice were obtained (Text S1).

**Figure 1:**
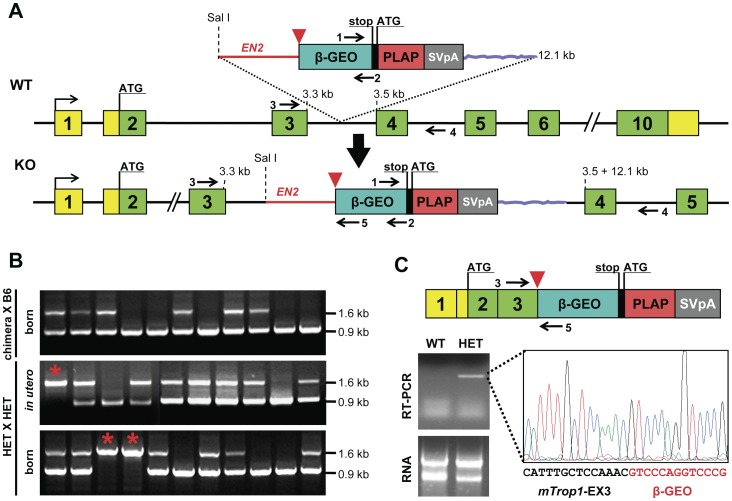
*mTrop1* gene trapping. (A) *mTrop1* gene inactivation scheme for the RST412 and RST413 ES clones. The inactivating exonic cassette within the pGT1TMPF gene-trap vector (top) contains an ATG-less ORF of β-galactosidase fused to neomycin phosphotransferase (β–GEO, cyan), followed by an internal ribosome entry site (black) for placental alkaline phosphatase (PLAP) ORF (red); simian virus 40 transcription termination/polyadenylation signal (SVpA, gray); an intronic sequence from *Engrailed2* (*EN2*) (red line) provides a strong splicing acceptor site (red arrowhead). The insertion brings the promoterless bicistronic β-GEO-PLAP-SVpA cassette in the third intron of WT *mTrop1* (middle). Cassette insertion leads to a null (KO) *mTrop1*, by leading to the splicing of the *mTrop1* exon 3 to the β-GEO cassette (*bottom*). Yellow boxes, *mTrop1* untranslated exon sequences; green boxes, *mTrop1* translated exons; arrows, PCR primer positions. (B) *mTrop1* multiplex PCR genotyping. F1 litters from RST412 chimeric mouse males crossed to B6 females (*top*). Litters from HET crossings, *in utero* at E9.5 (*middle*) and after birth at day 1 (*bottom*). The 0.9 kb PCR fragment is from the WT allele (primers 3 and 4), the 1.6 kb PCR fragment is from the KO allele (primers 3 and 5). Homozygous KO mice were identified both *in utero* and in litters at birth (red stars). (C) The fusion transcript from the KO allele (*top*) is expressed in the intestine of HET mice, as revealed by RNA reverse transcription (RT)-PCR (*bottom*, *left*) and sequencing (*bottom*, *right*), with in-frame fusion between *mTrop1* and β-GEO.

Hence, we resorted to a gene-trapping approach. Two gene-trapped ES clones were identified, i.e., RST412 and RST413, where *mTrop1* was demonstrated to be inactivated by insertion of a promoterless βGEO cassette (Fig. 1A, C; Text S1), with 5′ rapid amplification of cDNA ends (RACE) used for sequence validation. Both clones were used for blastocyst injection. Three and seven chimeric mice were obtained from clones RST412 and RST413, respectively (Fig. S3). All male chimeras (3 and 6 individuals, respectively) were bred to a C57BL/6 (B6) genetic background, to obtain first filial (F1) generation heterozygous (HET) mice bearing one null *mTrop1* allele (Fig. S3). Gene-specific genotyping was used throughout breeding to assess the *mTrop1* status of all of the littermates. Genomic and transcriptomic characterization of RST412 F1 mice showed *mTrop1*-specific insertion of the gene-trapping βGEO cassette and intestinal expression of the *mTrop1*-βGEO fusion transcript (Fig. 1B, C; Fig. S3B; Text S1). These HET mice were viable and fertile. RST413 F1 mice showed the βGEO marker, but no retention of the trapped *mTrop1* (Fig. S3B, C), possibly because of *in-vivo* genomic recombination [27], and these were not studied any further.

**Figure 2:**
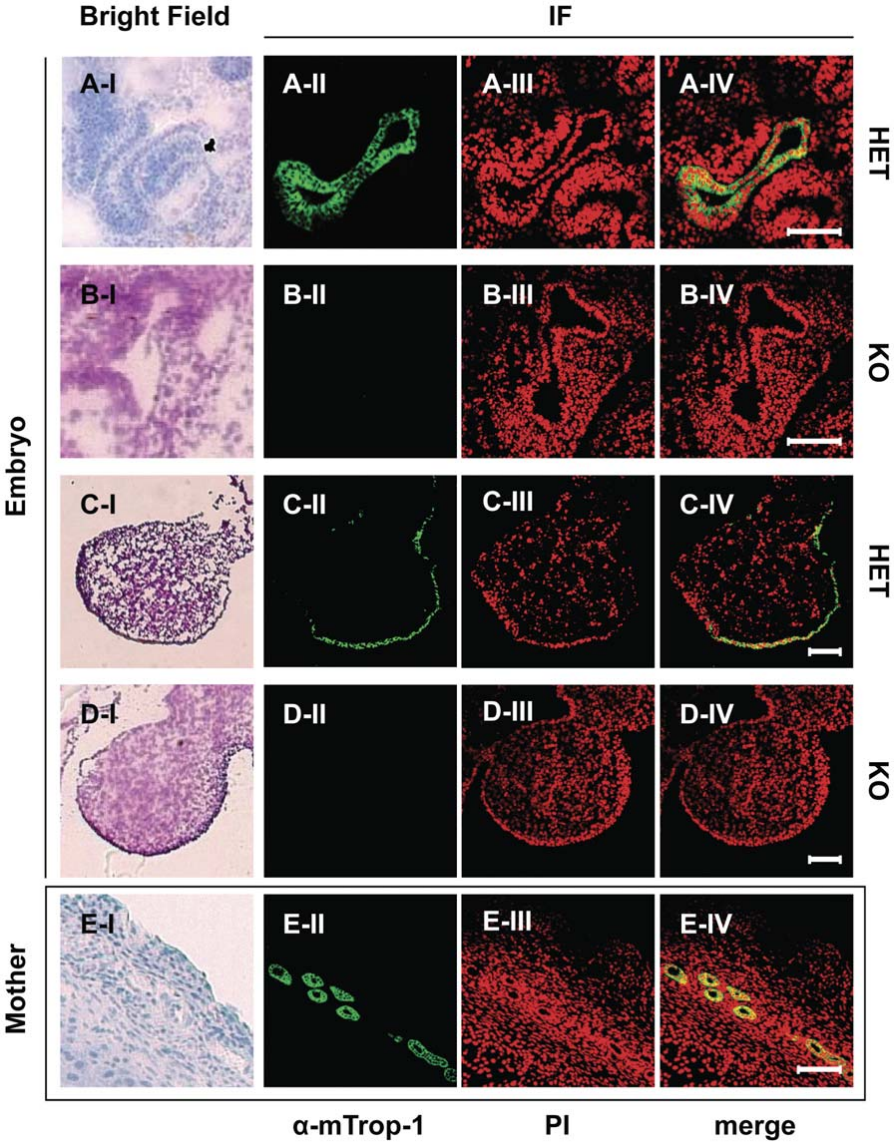
mTrop-1 protein expression in the developing mouse embryo. Confocal microscopy analysis of HET and KO embryos at E10.5. Frozen sections were stained with the G8.8 anti-mTrop-1 mAb (green); nuclei are stained with propidium iodide (PI; red) for context identification of immunofluorescent signals (merge). Matching bright field images are shown (left column) from consecutive frozen tissue sections stained with hematoxylin (A–I, E–I) or H&E (B–I, C–I, D–I). (A, B) Embryo intestine. (C, D) Embryo forelimb bud. (E) Mother uterine tissue. Embryonic tissues that express mTrop-1 at high levels are shown. HET mouse embryos show strong intestinal (A) and limb bud (C) epithelial staining localized at cell membranes, as expected. The same tissues from KO embryos (B, D) show complete absence of green signal. Uterine glands in maternal tissue express high levels of mTrop1 (E) and were used as stringent internal controls. Target tissue architecture and morphology in the HET embryos were normal. No gross differences were detected between HET and KO embryos. Scale bars: 100 µm.

To investigate embryonic development defects brought about by *mTrop1* ablation, we set up timed matings between HET mice from the RST412 colony, and we analyzed the litters *in utero* at embryonic day (E) 9.5–10.5. Homozygous gene-trapped KO embryos were indistinguishable from their wild-type (WT) and HET siblings in size, developmental stage, body symmetry and somite architecture. The embryo resorption rate was within the physiological range of healthy mouse colonies [28]. Immunofluorescence analyses with the anti-mTrop-1 G8.8 monoclonal antibody (mAb) demonstrated that mTrop-1 was absent in KO embryos (Fig. 2), thus confirming ablation of the protein in our mouse model. In WT and HET embryos, mTrop-1 expression was detected in the intestine, pharingeal cleft, nose placode, limb buds and other body-lining epithelia (Figs. S4, S5). Strong mTrop-1 staining in the nearby maternal uterine tissue (Fig. 2; Fig. S4D) provided a stringent internal control for all of the staining procedures.

**Figure 3:**
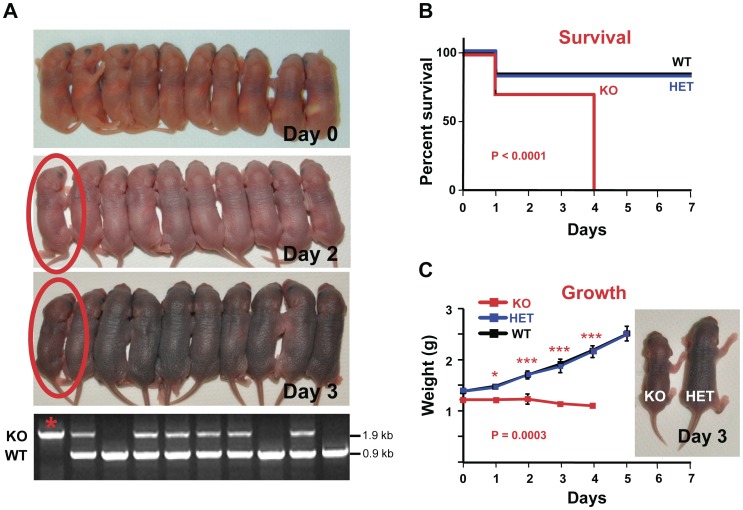
Growth arrest and early death of *mTrop1*-null mice. (A) Whole litter from a representative HET crossing. Day 0: At birth all of the 10 pups were alive; KO mice were indistinguishable from their littermates. Days 2, 3: One pup (circled in red) is appreciably smaller than the others. Bottom: genomic PCR genotyping identified the small pup as KO (star). The KO *versus* WT and HET littermate difference in weight increased over days 2, 3 and 4 (KO pup weight: 1.35 g, 1.20 g, 1.20 g, respectively; litter mean weight (±SD): 1.82±0.14 g, 2.11±0.29 g, 2.44±0.19 g, respectively). (B, C) analysis of 5 different litters from independent HET breeding pairs (all with >6 generations of backcrossing to B6). (B) Kaplan-Meier survival curves of WT, HET and KO newborns. No differences were revealed between WT and HET; all KO pups died by day 4 (P<0.0001 *versus* WT or *versus* HET, log-rank test) (C) Growth curves of WT, HET and KO pups; no differences were revealed between WT and HET (P = 0.7321, two-way ANOVA); KO pups showed no weight increase from birth to day 4 (KO vs WT: P = 0.0003, two-way ANOVA). Stars indicate statistically significant differences at single time points (Bonferroni multiple comparisons): * P<0.05; *** P<0.001. Inset: a KO mouse at day 3 *versus* a HET littermate.

These findings indicated a different pathogenetic course from that described by Nagao et al. [13]. Therefore, we analyzed litters from HET crossings at birth by *mTrop1-*specific genotyping. *mTrop*1-null newborn pups were found in essentially every litter analyzed (Fig. 1B; Table S2), at a frequency which was close to the 1∶2∶1 ratio expected for monogenic Mendelian inheritance (0.265 *versus* the expected 0.250; χ^2^ = 1.59; rejection threshold >5.99). These results demonstrated absence of negative selection *in utero* against the KO embryos. Consistently, KO pups at birth (day 0) appeared indistinguishable from their WT and HET littermates in size, morphology and behavior (Fig. 3A). However KO pups were no longer in the litter at weaning (4 weeks after birth) (Table S2), which suggested a negative effect of *mTrop1* inactivation early after birth. Hence, we went back to analysis of litters from birth. KO newborns were unable to gain weight (Fig. 3C) and appeared smaller than their siblings already at day 2 (Fig. 3A). This difference became even more apparent over the following 2 days (Fig. 3A), until death occurred, with 100% penetrance, by day 4 (Fig. 3B). No growth defects were observed in HET newborns, and their size, growth rate and post-natal survival were undistinguishable from WT pups (Fig. 3).

**Figure 4:**
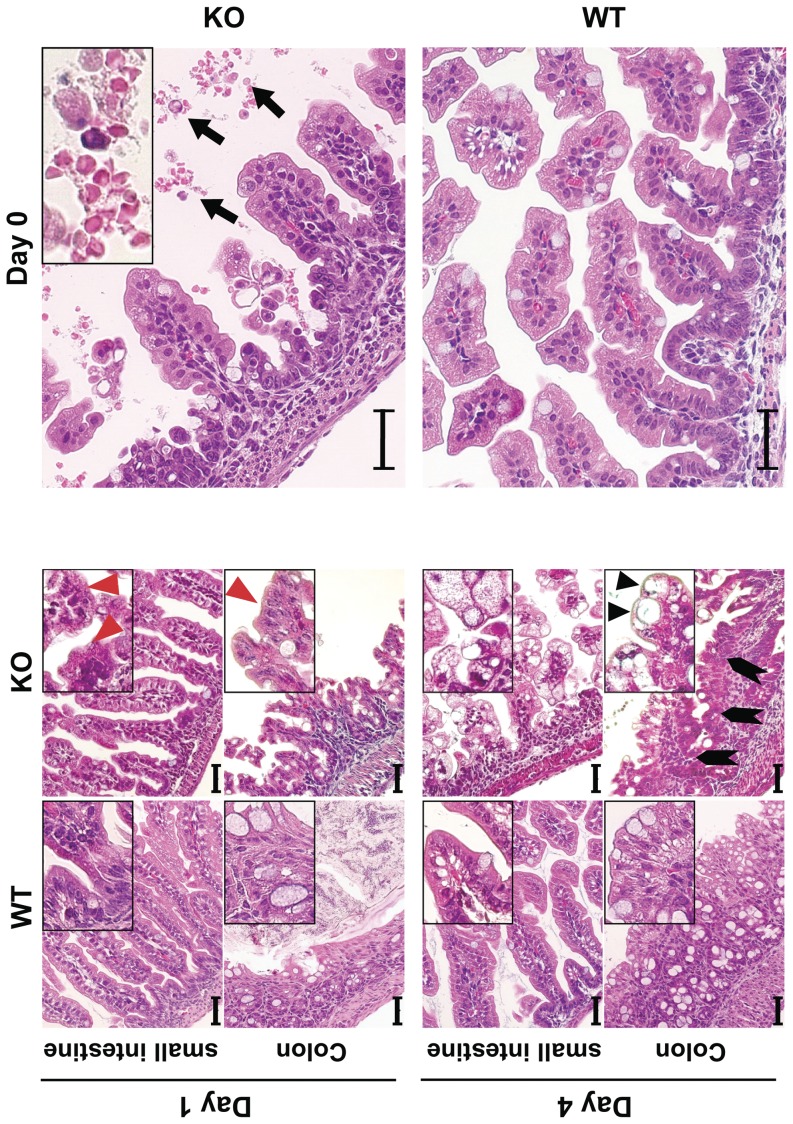
Tufting enteropathy in *mTrop1*-null mice. H&E staining of formalin-fixed paraffin-embedded small intestine and colon sections from WT and KO newborn mice, from day 0 to day 4. Insets: magnified areas. Villous atrophy was found throughout the small intestine of KO mice. Severity progressed from day 0 to day 4 (day of death). Red arrowheads: tufts of extruding epithelium, with surface enterocyte disorganization and focal crowding. These abnormalities were focally distributed, and increased over time, with highest tuft density at the time of death. Lymphocytes and plasma cells in the lamina propria were infrequent. KO colon crypts showed pseudo-cysts formation (black arrowheads) and abnormal regeneration with branching (block arrows). Hemorrhagic enteritis was apparent in the small intestine of KO mice from day 0 (top, right); black arrows: red blood cells in the intestinal lumen. Scale bars: 40 µm.

**Figure 5:**
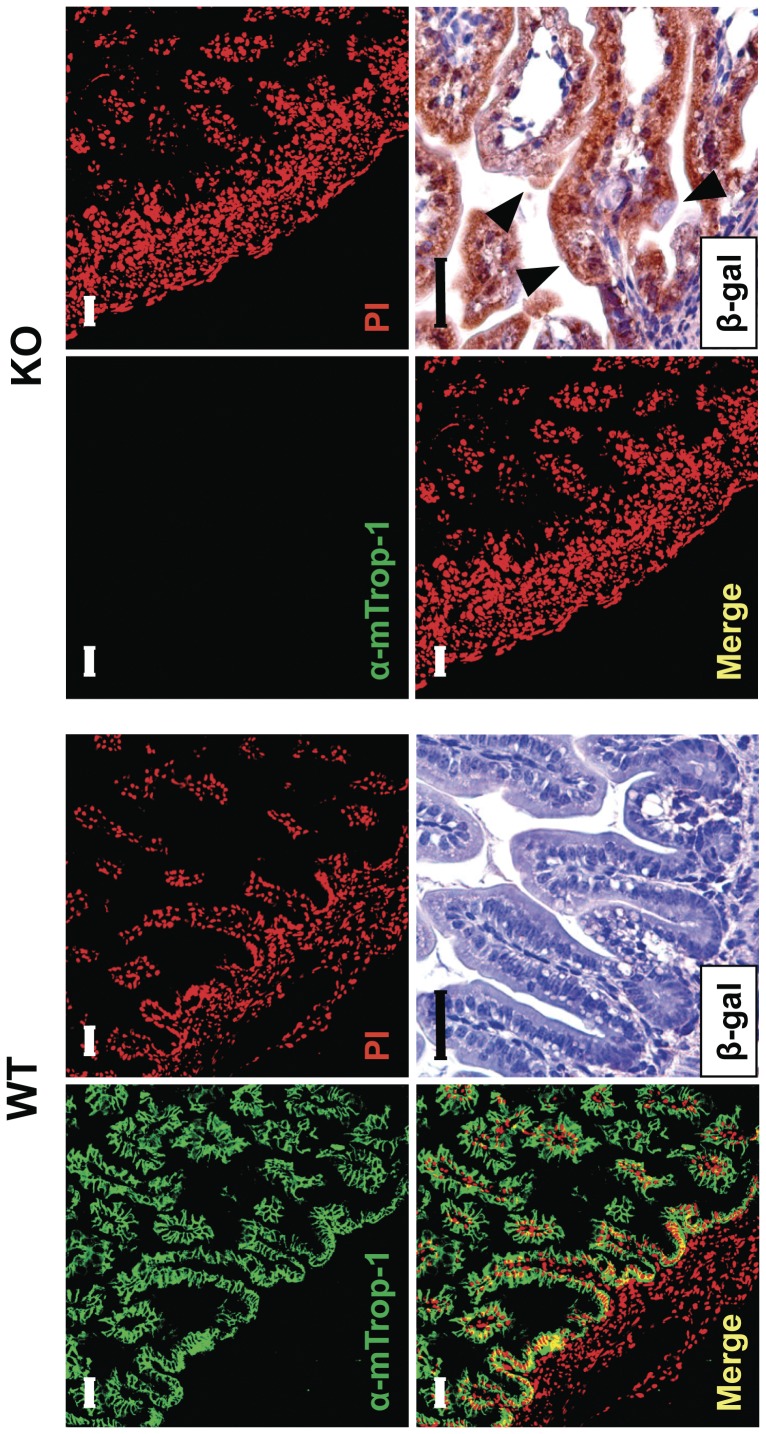
mTrop-1 expression in the intestine. Small intestine from WT (left) and KO (right) newborn pups at day 0, analyzed by immunofluorescence confocal microscopy. Staining with the G8.8 anti-mTrop-1 mAb (green). Nuclei were stained with PI (red) for context identification of immunofluorescent signals (merge). Expression of the β-gal marker from the *mTrop1* gene-trapped allele was analyzed by immunohistochemistry with anti-β-gal antibodies in sections of formalin-fixed, paraffin-embedded intestine. In WT tissues, a strong expression of mTrop-1 was observed, largely in the basolateral compartment of intestinal epithelial cells lining the villi. No β-gal staining was observed, as expected. In KO mice, mTrop-1 was completely absent (lack of green signal), while the positive staining for β-gal (brown deposits) confirmed the presence and specific expression of the gene-trapping cassette. The villi of the KO intestine showed tufting and crowding of epithelial cells (arrowheads). Scale bars: 40 µm.

Serial analysis of gene expression (SAGE) analyses and microarray hybridization profiles of embryonic tissues showed that *mTrop1* expression is highest in the intestinal epithelium (Fig. S6). Consistent with this, we found that mTrop-1 expression in the newborn intestine is high throughout the intestinal mucosa epithelial layer (see below). Taken together, these findings indicated that the intestine would be a primary target for defects linked to *mTrop1* ablation. Indeed, systematic macroscopic analyses showed that the intestine of 3-day old KO pups was smaller than the WT (Fig. S7), while other organs did not show macroscopic morphological defects. Histopathology analyses of the intestine showed villous atrophy of increasing severity, from minimal abnormalies at birth to essential loss of normal mucosal architecture by day 4 (Fig. 4; Fig. S7). Signs of hemorrhagic enteritis were evident from day 0 (Fig. 4). Surface enterocyte disorganization and crowding were focally distributed along the small intestine. Epithelial tufts increased over time, with the largest numbers in KO mice at day 4. These tufts were also observed in the colon, consistent with the histopathology of human CTE. Colon crypts showed aberrant pseudo-cyst aspects and highly proliferative enterocytes (Fig. 4; Fig. S7). No microscopic abnormalities were apparent in other organs (Fig. S8). In particular, no abnormalities were detected in the esophagous and stomach, which express little if any Trop-1 under normal conditions ([4]; Fig. S6). Immunofluorescence analysis showed no mTrop-1 in the intestine of newborn KO mice, confirming full inactivation of the gene (Fig. 5). On the other hand, the gene-trapping β-gal marker was only detected in KO (Fig. 5) and HET mice, as from the gene inactivation strategy.

Taken together, these findings show that *mTrop1* loss is a single-gene cause of CTE, which leads to severe structural alterations in the intestinal mucosa, with loss of epithelial architecture and barrier function [18], [19]. Of relevance, decreased expression of Trop-1 was first suggested to lead to CTE in human subjects [16], [22], while complete absence of expression of Trop-1 was revealed in additional cases [22], [23]. However, a less severe form of CTE associates with specific mutations of the *TROP1* gene, e.g., c.498insC [20], which possibly reflects a residual activity/expression of Trop-1, as for cases with *TROP1* exon 4 skipping [16].

### *mTrop1* loss disrupts intestinal E-cadherin/β-catenin expression and localization

**Figure 6:**
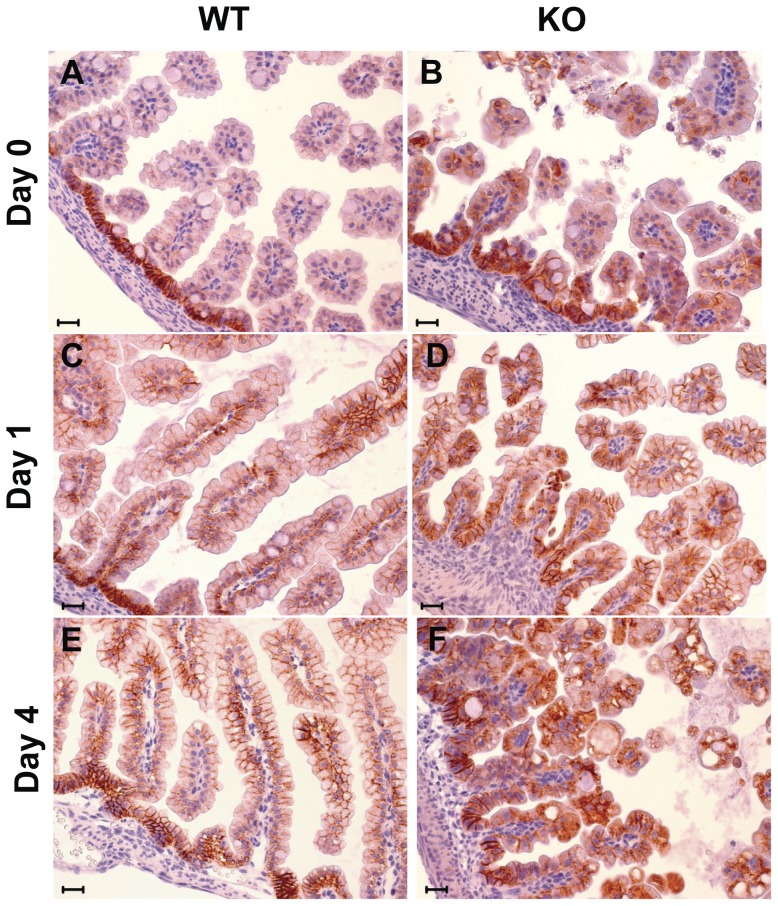
E-cadherin expression in the intestine. E-cadherin immunostaining pattern in small bowel of WT (wt) and KO mice at birth (day 0), and at day 1 and day 4 after birth. E-cadherin shows a typical membrane immunoreactivity in WT mice (A, C, E), whereas in KO mice (B, D, F) it is localized increasingly in the cytoplasm, with a prevalent cytoplasmic accumulation and membrane-disrupted pattern at day 4 after birth. (Scale bar: 20 mm).

Trop-1 ablation in zebrafish embryos was shown to cause a decrease in membrane-bound E-cadherin [24]. E-cadherin and its interactor β-catenin are essential components for adherens junction assembly. Adherens junctions mediate cell-cell contact in epithelia and modulate the actin cytoskeleton, to preserve cell structure and polarity, and ultimately epithelium integrity. E-cadherin ablation from adult mouse intestine has indeed been shown to destroy epithelial architecture, causing hemorrhagic diarrhea [29]. Consistent with this, intestine-specific E-cadherin ablation in the mouse embryo caused perinatal death with severe disruption of intestinal morphogenesis [30]. Therefore we investigated whether intestinal epithelium disruption in CTE mice was linked to alterations of E-cadherin/β-catenin. Immunohistochemistry analyses of intestine from day 0 to day 4 after birth showed that E-cadherin localized in the basolateral membrane compartment of WT epithelial cells (Fig. 6A, C, E). Here E-cadherin showed the highest expression levels in the intervillar epithelium and developing crypts, which are the sites of the most active cell proliferation. E-cadherin expression along the villi was weak at birth, and markedly increased at cell-cell junctions in the following days (Fig. 6A, C, E). E-cadherin/β-catenin complexes continuously recyle between the plasma membrane and perinuclear endocytic internal compartments [31]. This dynamic process is essential for preserving the integrity of epithelia during morphogenetic movements [31]. In the KO mice, epithelial E-cadherin expression was altered from birth, with marked expression in villar epithelium (Fig. 6B, D, F). This was accompanied by cytoplasmic localization, probably because of retention in the endoplasmic reticulum or in other membranous re-cycling compartments [31]. E-cadherin polarization was then progressively lost, with a disorganized transition between intervillar regions and villar epithelium. Intracellular E-cadherin accumulation was then shown to rapidly increase from day 0 until the time of death (Fig. 6B, D, F).

**Figure 7:**
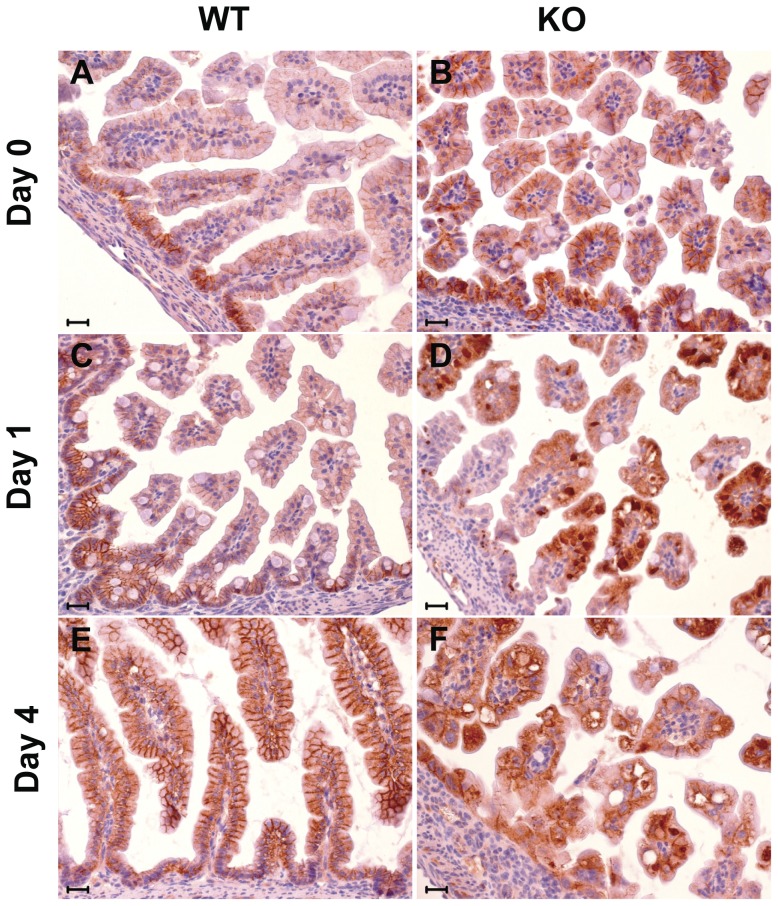
β-catenin expression in the intestine. β-catenin immunostaining pattern in small bowel of WT and KO mice at birth (day 0), and at day 1 and day 4 after birth. Membrane pattern is typically seen in WT mice (A, C, E). A progressive cytoplasmic immunoreactivity, with concomitant diffuse and “dot-like” perinuclear patterns, and disruption of the membrane immunoreactivity for β-catenin is seen in KO mice (B, D, F) starting from day 1 after birth.(Scale bar: 20 mm).

Parallel immunohistochemistry analyses were performed for β-catenin (Fig. 7). In WT intestinal epithelial cells, β-catenin expression was also localized to the basolateral membrane compartment and showed marked polarization (Fig. 7A, C, E). This showed a shift from birth, where it was strongest in the intervillar region, to day 4, where it reached the highest levels in the villous epithelium. In the KO epithelium, both intervillar and villous epithelia showed strong β-catenin membrane staining at day 0, together with anomalous perinuclear intracellular deposits (Fig. 7B, D, F), which are associated with disruption of signaling along the canonical Wnt/β-catenin pathway [32]. β-catenin intracellular accumulation might also associate with Trop-1-dependent aberrant nuclear translocation [33]. Consistent with this, intracellular deposits were predominant from day 1 to day 4 after birth, leading to almost complete loss of membrane staining at the time of death (Fig. 7F).

*In vitro* studies have shown that Trop-1 can abrogate E-cadherin-mediated cell–cell interactions by disrupting the link between α-catenin and F-actin [34]. Ectopic expression of Trop-1 in cadherin-positive cells leads to the abrogation of adherens junctions and to an increase of Trop-1-mediated intercellular junctions [7]. In the *TROP1*-KO zebrafish embryo, E-cadherin restoration does not alleviate the epithelial defects, which indicates that Trop-1 regulation of E-cadherin trafficking is likely to act through additional Trop-1 target(s) rather than a direct interaction with E-cadherin [24]. In human multipotential hematopoietic cells, Trop-1 is associated with a cytoplasmic complex that is enriched in actin-binding proteins such as afadin, α-actinin, ezrin and vinculin. Among these, ezrin can interact directly with E-cadherin/β-catenin [35] and regulate E-cadherin membrane trafficking and adherens junction formation [36], [37], which suggests that ezrin has a role in mediating Trop-1 regulation of E-cadherin/β-catenin dynamics.

## Conclusions

Our results are consistent with Trop-1 loss being a single-gene cause of CTE. The *mTrop1* KO mouse thus provides a much needed animal model for understanding the pathogenesis of intestinal alterations in CTE and as a benchmark for developing novel therapeutic approaches. Our findings also unravel an essential role for Trop-1 in the maintenance of intestinal architecture and functionality, through regulation of E-cadherin/β-catenin expression and cellular localization.

## Supporting Information

Text S1 **Supporting** **Materials and Methods** **; Supporting Results; Supporting References.**
(DOC)

Table S1 **Primer sequences.**
(DOC)

Table S2 **Genotype frequencies in litters from heterozygous** ***mTrop1*** **+/−×** ***mTrop1*** **+/− crossings.**
(DOC)

Figure S1 ***mTrop1*** **inactivation by targeted gene replacement.**
(TIF)

Figure S2 ***mTrop1*** **-targeted ES clone.**
(TIF)

Figure S3 ***mTrop1*** **gene-trapped mice.**
(TIF)

Figure S4 **Whole-embryo assessment of mTrop-1 expression.**
(TIF)

Figure S5 **Embryonic mTrop-1 expression.**
(TIF)

Figure S6 ***mTrop1*** **expression in the developing embryo.**
(TIF)

Figure S7 **Intestinal tract abnormalities in** ***mTrop1*** **-null mice.**
(TIF)

Figure S8 **Histology of tissues from WT and KO mice.**
(TIF)
