# Correction: *mTrop1/Epcam* Knockout Mice Develop Congenital Tufting Enteropathy through Dysregulation of Intestinal E-cadherin/β-catenin

**DOI:** 10.1371/annotation/4336e050-68c3-48ef-869e-80c2232b7e65

**Published:** 2013-05-03

**Authors:** Emanuela Guerra, Rossano Lattanzio, Rossana La Sorda, Francesca Dini, Gian Mario Tiboni, Mauro Piantelli, Saverio Alberti

The mTrop1 knock-out mouse project, which led to the present published article, started at the Consorzio Mario Negri Sud, Santa Maria Imbaro, Chieti, Italy, after approval granted by the Italian Ministry of Health with Ministerial Decree Prot n° 24/2001 B on the 17th of May 2001. Ministerial approval was then confirmed when we moved to our current Research Institution (University G. d' Annunzio Foundation, Chieti, Italy, Prot n° 407/2006, on the 26th June 2006), with due communication to local authorities as instructed by Italian law (Decree N°116/1992 and Application Notes N° 8/1994). 

