# Supplementary material for: mTrop1/Epcam Knockout Mice Develop Congenital Tufting Enteropathy through Dysregulation of Intestinal E-cadherin/β-catenin
Source: PLoS One. 2012 Nov 28;7(11):e49302. doi: 10.1371/journal.pone.0049302 (PMC3509129; doi:10.1371/journal.pone.0049302)
Supplement: Text S1 — Supporting Materials and Methods ; Supporting Results; Supporting References. (DOC) [file pone.0049302.s001.doc]

**SupPORTING TEXT**

Title: “*mTrop1*/*Epcam* knockout mice develop congenital tufting enteropathy through dysregulation of intestinal E-cadherin/ β-catenin”

Emanuela Guerra, Rossano Lattanzio, Rossana La Sorda, Francesca Dini, Gian Mario Tiboni, Mauro Piantelli and Saverio Alberti

**Supporting Materials and Methods**

**Gene replacement in embryonic stem (ES) cells**

The pFM54 vector was used for gene replacement. This was kindly provided by Dr. L. Ronfani, San Raffaele, Milan, Italy, and is a derivative of pPNT [1] with positive (neomycin phosphotransferase gene, *neo*) and negative (thymidilate kinase gene from herpes virus, TK) selection, each expressed from a mouse phosphoglycerokinase (PGK) promoter. The TBV-2 ES cell line, obtained from 129S2/SvPas mice, was used for homologous recombination. ES cells were grown in Dulbecco’s Modified Eagle Medium (DMEM) with 15% (v/v) heat-inactivated ES-grade fetal calf serum (FCS), 2 mM glutamine, 1× nonessential amino acids, 1 mM sodium pyruvate, 0.1 mM 2-mercaptoethanol, 50 U/ml penicillin and 50 µg/ml streptomycin (Invitrogen, Carlsbad, CA) and 1,000 units/ml leukemia inhibitory factor (LIF) (Esgro, Chemicon, Temecula, CA). Cells were cultured over a feeder layer of mitomycin-C-treated mouse embryonic fibroblasts (MEFs). The MEFs were obtained following trypsin digestion (0.05% trypsin in 0.53 mM EDTA at 37 °C for 30 min) of B6 mouse embryos at day of gestation (E)14.5. TBV-2 clones were selected in 200 μg/ml G418 (Invitrogen), manually picked, expanded, and stored in liquid nitrogen. Replicas were grown on gelatin-coated 96-well plates for DNA extraction. The D1 positive clone was grown on mitomycin-C-treated MEFs for morphological and flow cytometry analyses and for B6 blastocyst injection. This clone is available to the scientific community.

**DNA extraction**

DNA was extracted from ES clones as follows: 50 µl 100 mM Tris-Cl, pH 8, 10 mM EDTA, pH 8, 10 mM NaCl, 0.5% Sarcosyl, 1 mg/ml fresh proteinase K was added to each well. Plates were incubated at 60 °C overnight in a humidified chamber. One hundred µl cold ethanol, 75 mM NaCl was added, and the plates were incubated at room temperature for at least 60 min. The plates were centrifuged at 1,200× *g* for 5 min, the supernatant was discarded, and the DNA pellets were washed twice with 70% cold ethanol. For Southern blotting, the DNA was resuspended directly in restriction enzyme mix (New England Biolabs, Beverly, MA) and incubated overnight at 37 °C. Tail biopsies (0.5 cm), were incubated in 500 µl 50 mM Tris-Cl, pH 8, 100 mM EDTA, pH 8, 100 mM NaCl, 1% SDS and 1 mg/ml fresh proteinase K at 56 °C overnight with constant stirring; 200 µl 4.2 M NaCl, 0.63 M KCl and 10 mM Tris-Cl, pH 8, was added, with incubation at 4 °C for 60 min with constant stirring. The tubes were centrifuged for 20 min at maximum speed in a microfuge, the supernatant was tranferred to a clean tube, and the DNA was precipitated with 1 vol. isopropanol, washed with 2 vol. 70% ethanol and resuspended in 100 µl 1 mM Tris-Cl, pH 8, 0.1 mM EDTA, pH 8, and left at room temperature overnight. For embryonic tissues, 5 µm sections from “fresh” (embedded in optimal cutting temperature (OCT) compound and snap frozen in liquid N_2_,) or formalin-fixed, paraffin-embedded embryos were collected on glass slides. Tissues of interest were identified, scraped with a sterile needle and transferred to a polymerase chain reaction (PCR) tube. These were then digested in 40 µl 100 mM Tris-Cl, pH 8, 2 mM EDTA, pH 8, 1% Tween 20 (v/v) and 1 mg/ml fresh proteinase K at 56 °C overnight. One µl of this crude extract was used for PCR genotyping.

**Southern blotting**

Southern blotting was performed as previously described [2]. The probes were PCR fragments of about 300 bp in length, and they were [^32^P]-labeled by specific priming [3]. The probe sequences were chosen using repetitive and low complexity sequence analysis filters (<rodent repeat masker>, <dust>). SacI and SacI/KpnI digested genomic DNAs were hybridized at high stringency with the 5’ and the 3’ probes respectively, according to the strategy depicted in Figure S1.

**PCR optimization**

Primers were designed using Primer3 ([frodo.wi.mit.edu/primer3/](http://frodo.wi.mit.edu/primer3/)) [4] (Table S1). Different combinations of forward and reverse primers were tested using several different DNA polymerases (AmpliTaq Gold DNA polymerase, Applied Biosystems, Carlsbad, CA; HotMaster Taq DNA Polymerase, Eppendorf, Hamburg, Germany; GC*-*RICH PCR System, Roche Applied Sciences, Penzberg, Germany; Failsafe PCR System, Epicentre, Madison, WI), together with different reaction buffers (buffers A-I, FailSafe optimization set) and annealing temperatures (from 55 °C to 68 °C; gradient thermalcycler Mastercycler ep, Eppendorf).

**RNA extraction and reverse transcription (RT)-PCR**

RNA was purified by acid guanidinium thiocyanate-phenol-chloroform extraction [5] from liquid-nitrogen snap-frozen intestines. One µg of total RNA was reverse transcribed with ImProm-II Reverse Transcriptase (Promega, Madison WI) using random nonamers priming according to the manufacturer protocol. Actual cDNA amounts were quantified by ethidium bromide fluorescence in solution [6]. Nested PCR with primers mT1EX2-F1/ β-gal-Baygen-R2 followed by mT1EX2-F2/β-gal-Baygen-R3 (Table S1) was used to detect the *mTrop1*-βGEO gene fusion.

**DNA sequencing**

DNA sequencing was performed with the Sanger method [2]. DNA sequences were analyzed using Genetics Computer Group programs [7].

**DNA microarray analysis**

DNA microarray data for *mTrop1* expression in normal mouse tissues were retrieved from the NCBI GEO Profiles public database ([www.ncbi.nlm.nih.gov/geoprofiles](http://www.ncbi.nlm.nih.gov/geoprofiles)).

**Serial analysis of gene expression (SAGE)**

SAGE analysis data for m*Trop1* expression were retrieved from the SAGE public database ([cgap.nci.nih.gov/SAGE/](http://cgap.nci.nih.gov/SAGE/)). Short sequences (10-14 bp) from the 3’ region of *mTrop1* mRNA were used as unique identification tags; quantitative expression data were obtained from sequenced tag concatamers, and displayed as SAGE Expression Matrix.

**Supporting Results**

***mTrop1* inactivation by targeted gene replacement**

m*Trop1* inactivation was pursued by targeted gene replacement [8] in the TBV-2 mouse ES cell line. To obtain suitable homology arms a mouse genomic 129SV Fix II phage library [9] was extensively screened at high stringency with the EGP-314 *mTrop1* cDNA probe [10]. Following this approach, we had previously isolated two clones containing *mTrop1* exons VI, VII and VIII, and IX and X, respectively [9]. However, we did not succeed in isolating the 5' end of the gene. Sequence analysis of this region from the Mouse Genome Project (NT_039649.7, nt 74229105-74244253) showed extensive presence of low complexity and repetitive regions, which hamper specific probe hybridization. Therefore we sought to obtain homology arms by genomic PCR. To optimize the primers and PCR parameters, a 10 kb genomic region at the 5’ end of the *mTrop1* gene was systematically probed using PCR primers spaced 500 bp apart. The C57BL/6 (B6) mouse BAC RP24-315D17 (AC163652.3) containing the complete *mTrop1* gene and surrounding regions was used as a template for reconstitution PCR. All of the amplified segments were verified by sequencing. PCR reactions were then performed on 129SV-derived TBV-2 ES line genomic DNA. This led to the amplification of a 1267 bp fragment located 564 bp upstream of exon 1 (5’ arm; primers XII-for/XIII-rev; Table S1). However, we failed to obtain an isogenic 3’ arm of suitable length by genomic PCR on 129SV genomic DNA. We noted that the sequences of the PCR fragments from the 129SV genomic DNA were essentially identical to those from B6 mice (Mouse Genome database), which indicated high inter-strain conservation of this region. Hence, we decided to use a 3700 bp fragment (from intron 2 to intron 4), as amplified from the B6 BAC DNA with primers 3-XVIfor/3-XVIIbis (Table S1) as the 3’ homology arm. The 5’ and 3’ arms were cloned into the pFM54 vector at the KpnI/BamHI (5’) and SalI/NotI (3’) sites flanking the floxed PGK-*neo* cassette. This is predicted to create a null allele by replacing a 2341-bp region at the 5’ end of the *mTrop1* gene, containing the transcriptional and translational start sites and the first 27 codons of the *mTrop11* open reading frame (ORF), with a PGK-*neo* selection cassette (Fig S1). The targeting vector was linearized at the Not I site, and electroporated into TBV-2 ES cells. Three hundred and forty five G418 and ganciclovir resistant clones were screened by Southern blotting (Figs S1,S2). A D1 positive clone was identified that showed the predicted hybridization patterns for correct targeting of one of the two copies of the *mTrop1* gene. Southern blotting of the *mTrop1* 5' region of the D1 clone showed the expected 16.3 and the 5.6 kb hybridizing bands for the wild type (WT) and the gene-targeted *mTrop1* allele, respectively (Fig S2a). Correct targeting was confirmed by Southern blotting of the *mTrop1* 3' region of the D1 clone, which showed the predicted 14.2 (WT) and 8.8 (targeted) hybridizing bands (Fig S2a). The D1 clone showed normal morphology (Fig S2c) and karyotype. mTrop-1 protein levels in the recombinant clone were similar to those of the parental cell line (Fig S2b). Hence, one normal gene copy is sufficient to provide WT mTrop-1 protein levels, consistent with the recessive behavior of *TROP1* mutations in congenital tufting enteropathy (CTE). The D1 clone was then used for injection into B6 blastocysts. This led to a total of 161 embryos, from four distinct rounds of injection. Thirty-four pups were born, but no chimeric mice were obtained.

**Sequence of the *mTrop1*-βGEO locus**. *mTrop1*, β-galactosidase-neomycin phosphotransferase fusion (βGEO) (ATG-less) and placental-like alkaline phosphatase (PLAP) ORFs are in red. Arrowhead: start of transcription; yellow highlight: first codon of the mTrop1 ORF; red highlight: stop codons. Grey highlight: pGT1TMPFS gene-trap vector sequence; the encephalomyocarditis virus internal ribosome entry site (IRES) between βGEO and PLAP and the simian virus (SV) 40 virus polyadenylation signal downstream of the PLAP ORF are underlined. Primers are in magenta.

**upstream untranscribed sequence**

**..........cactcccacccctccaaagacccataaaacccaggtgtggaggagaagca**

**mTrop1-EX1**

**GCACTTCTTCCCTCTGATGGACTGATTCTGCACGTGAGACCTGCGGCGGCGGCGGCGGCG**

**mTrop1-int1 385 bp**

**GCTGCTGCAGCTGCAGCTGCATGTTTCACTAGAGgtctttcctcgattttttttttttt.**

**mTrop1-EX2**

**........gtattgtctattgtctttcgtccagGAAGTTCTCGGTTTGACTTGGTATCC**

**CTTTCGGCTTTCACGTCCAGTCTCGTCCGTGTGTCCGCGACATCCGGTCCTTCCGGGGTA**

**CTGGAATCCCCGCCTCTGGTCCGGGACAGCGCACACCTGGAGAGGGGGCGAGGTGGGGCG**

**GGTGAGTCACCGCGGGCGAGCGGGCGGGTGGGCGGGCGAGCGGAGGTGAGGGCGGGGAGG**

**GGCGTGGCCGGCCGCCGGGGCAGCAGATCCGCAGGTCCGCTCCCGCCTCGCCGCGCGCAC**

**AGCGCTCAGTCCGTCCGCCGCCGCGCAGCGCGACTGTCCTCCGAGCCGTCCCGCGCCGCA**

**mTrop1-ORF**

**CCTCCGCGAGTCGCCCTCGCCGCTCCGCGCGCAGCATGGCGGGTCCCCAGGCCCTCGCGT**

[**M**](javascript:M(1)) [**A**](javascript:A(2)) [**G**](javascript:G(3)) [**P**](javascript:P(4)) [**Q**](javascript:Q(5)) [**A**](javascript:A(6)) [**L**](javascript:L(7)) [**A**](javascript:A(8)) [**F**](javascript:F(9))

**mTrop1-int2**

**3001 bp**

**TCGGGCTCCTGCTCGCGGTGGTCACAGCGACGCTGGCCGCGGCTCAGAGAGgtgaggcgt**

[**G**](javascript:G(10)) [**L**](javascript:L(11)) [**L**](javascript:L(12)) [**L**](javascript:L(13)) [**A**](javascript:A(14)) [**V**](javascript:V(15)) [**V**](javascript:V(16)) [**T**](javascript:T(17)) [**A**](javascript:A(18)) [**T**](javascript:T(19)) [**L**](javascript:L(20)) [**A**](javascript:A(21)) [**A**](javascript:A(22)) [**A**](javascript:A(23)) [**Q**](javascript:Q(24)) [**R**](javascript:R(25)) [**D**](javascript:D(26))

**mTrop1-EX3**

**ggacctggggcggagg..........tttttttttttttacaattttctagACTGTGTCT**

[**C**](javascript:C(27)) [**V**](javascript:V(28)) [**C**](javascript:C(29))

**Primer mT1EX2-F2**

**GTGACAACTACAAGCTGGCAACAAGTTGCTCTCTGAATGAATATGGTGAATGCCAGTGTA**

[**D**](javascript:D(30)) [**N**](javascript:N(31)) [**Y**](javascript:Y(32)) [**K**](javascript:K(33)) [**L**](javascript:L(34)) [**A**](javascript:A(35)) [**T**](javascript:T(36)) [**S**](javascript:S(37)) [**C**](javascript:C(38)) [**S**](javascript:S(39)) [**L**](javascript:L(40)) [**N**](javascript:N(41)) [**E**](javascript:E(42)) [**Y**](javascript:Y(43)) [**G**](javascript:G(44)) [**E**](javascript:E(45)) [**C**](javascript:C(46)) [**Q**](javascript:Q(47)) [**C**](javascript:C(48)) [**T**](javascript:T(49))

**mTrop1-int3 284 bp**

**CTTCCTATGGTACACAGAATACTGTCATTTGCTCCAAACgtgagtaaatcaatcttctta**

[**S**](javascript:S(50)) [**Y**](javascript:Y(51)) [**G**](javascript:G(52)) [**T**](javascript:T(53)) [**Q**](javascript:Q(54)) [**N**](javascript:N(55)) [**T**](javascript:T(56)) [**V**](javascript:V(57)) [**I**](javascript:I(58)) [**C**](javascript:C(59)) [**S**](javascript:S(60)) [**K**](javascript:K(61)) [**K**](javascript:L(62))

**ttgcctggaagtttaatctcgataatacttttcaaatcgaggctcctcgagttgttttat**

**tggctctaatctgttattttgtggaaagtatcttcagccagtaattcagtgcgg.....**

**Engrailed 2 intron, 1721 bp**

**gtcgaccgagcttggaattcatgggaagaggaaccgaaagtatgtttttcagatgttctt**

**tctcagaaataggagtttgcggaggttggagtgtgtgttgtaggacacgaaccccagggt**

**ggaggagactggaggacagagccctctttcccagggagggaaggaggagagtttgagatc**

**cgctccggaagtcggggttcaggtttgagcaggccaggcctctcccgtggtctcgccctc**

**ttgtcctagaagcctcactggccaggtgtaagccaggtcgtgggtgccgagccctgctcc**

**ctcatcctcagcatggatgtgaagaggactgtatggcgtgcgggtgtgtgtgaccgtggg**

**tacacttaaaacaccgggttttggatctgcactgtcccggatgtcctctggtgctcaaag**

**acccttttgggtttgccctttggtaagagcgccgggatctacttgtctggaggccaggga**

**gtcctcagccgaggcttgccgcccctgactgcactgcactgagtagtggatgggagagtc**

**tggtaccgcactgccggtttcctccaccatccccgcagcgcagggcagtgcattccgtcc**

**tggctgcgaagggggatggtcgggccttctccagcctcttccgcttctagcgaaggggcc**

**ttgatggaagggcccgcatgtctccaaagttgattcatgcttcttgcacagagaaagacc**

**agaaagaaggtctcaagttttagccggtagcccggatggccttttcctgcacggcaccat**

**atgaaccttgtgaccctgactttgagacccctctaacccaaggcccctaccactttaccc**

**tttccctttgaaggctttcccacaccaccctccacacttnccccaaacactgccaactat**

**gtaggaggaaggggttgggactaacagaagaacccgttgtggggaagctgttgggagggt**

**cactttatgttcttgcccaaggtcagttgggtggcctgcttctgatgaggtggtcccaag**

**gtctggggtagaaggtgagagggacaggccaccaaggtcagccccccccccctatcccat**

**aggagccaggtccctctcctggacaggaagactgaaggggagatgccagagactcagtga**

**agcctggggtaccctattggagtccttcaaggaaacaaacttggcctcaccaggcctcag**

**ccttggctcctcctgggaactctactgcccttgggatcccttgtagttgtgggttacata**

**ggaaggggacggattccccttgactggctagcctactcttttcttcagtcttctccatct**

**cctctcaccgttctctcgaccctttccctaggatagacttggaaaaagataaggggagaa**

**aaacaaatgcaaacgaggccagaaagattttggctgggcattccttccgctagcttttat**

**tgggatcccctagtttgtgataggccttttagctacatctgccaatccatctcattttca**

**cacacacacacaccactttccttctggtcagtgggcacatgtccagcctcaagtttatat**

**caccacccccaatgcccaacacttgtatggccttggcgggtcatccccccccccaccccc**

**agtatctgcaacctcaagctagcttgggtgcgttggttgtggataagtagctagactcca**

**βGEO ORF (startless)**

**gcaaccagtaacctctgccctttctcctccatgacaaccagGTCCCAGGTCCCGAAAACC**

[**P**](javascript:P(575)) [**R**](javascript:R(576)) [**S**](javascript:S(577)) [**R**](javascript:R(578)) [**K**](javascript:K(579)) [**P**](javascript:P(580))

**Primer β-gal-Baygen-R3**

**AAAGAAGAAGAACGCAGATCGCAGATCGCAGATCCAGAAGTTCCTATTCCGAAGTTCCTA**

[**K**](javascript:K(581)) [**K**](javascript:K(582)) [**K**](javascript:K(583)) [**N**](javascript:N(584)) [**A**](javascript:A(585)) [**D**](javascript:D(586)) [**R**](javascript:R(587)) [**R**](javascript:R(588)) [**S**](javascript:S(589)) [**Q**](javascript:Q(590)) [**I**](javascript:I(591)) [**Q**](javascript:Q(592)) [**K**](javascript:K(593)) [**F**](javascript:F(594)) [**L**](javascript:L(595)) [**F**](javascript:F(596)) [**R**](javascript:R(597)) [**S**](javascript:S(598)) [**S**](javascript:S(599)) [**Y**](javascript:Y(600))

**TTCTCTAGAAAGTATAGGAACTTCTCAGATCTGCGGGCTGCAGGGAGAGTTGAGATGGAA**

[**S**](javascript:S(601)) [**L**](javascript:L(602)) [**E**](javascript:E(603)) [**S**](javascript:S(604)) [**I**](javascript:I(605)) [**G**](javascript:G(606)) [**T**](javascript:T(607)) [**S**](javascript:S(608)) [**Q**](javascript:Q(609)) [**I**](javascript:I(610)) [**C**](javascript:C(611)) [**G**](javascript:G(612)) [**L**](javascript:L(613)) [**Q**](javascript:Q(614)) [**G**](javascript:G(615)) [**E**](javascript:E(616)) [**L**](javascript:L(617)) [**R**](javascript:R(618)) [**W**](javascript:W(619)) [**K**](javascript:K(620))

**GGCAGAGAAGGCTCCTTCTTCCCAGTCCTGGATCACCTTCTCCCTAAAGAACCAAAAGGT**

[**A**](javascript:A(621)) [**E**](javascript:E(622)) [**K**](javascript:K(623)) [**A**](javascript:A(624)) [**P**](javascript:P(625)) [**S**](javascript:S(626)) [**S**](javascript:S(627)) [**Q**](javascript:Q(628)) [**S**](javascript:S(629)) [**W**](javascript:W(630)) [**I**](javascript:I(631)) [**T**](javascript:T(632)) [**F**](javascript:F(633)) [**S**](javascript:S(634)) [**L**](javascript:L(635)) [**K**](javascript:K(636)) [**N**](javascript:N(637)) [**Q**](javascript:Q(638)) [**K**](javascript:K(639)) [**V**](javascript:V(640))

**GTCTGTGCAGAAGTCTACTAGCAACCCCAAGTTCCAGCTGTCCGAAACGCTCCCACTCAC**

[**S**](javascript:S(641)) [**V**](javascript:V(642)) [**Q**](javascript:Q(643)) [**K**](javascript:K(644)) [**S**](javascript:S(645)) [**T**](javascript:T(646)) [**S**](javascript:S(647)) [**N**](javascript:N(648)) [**P**](javascript:P(649)) [**K**](javascript:K(650)) [**F**](javascript:F(651)) [**Q**](javascript:Q(652)) [**L**](javascript:L(653)) [**S**](javascript:S(654)) [**E**](javascript:E(655)) [**T**](javascript:T(656)) [**L**](javascript:L(657)) [**P**](javascript:P(658)) [**L**](javascript:L(659)) [**T**](javascript:T(660))

**CCTTCAGATACCCCAGGTCTCCCTTCAGTTTGCTGGTTCTGGCAACCTGACCCTGACTCT**

[**L**](javascript:L(661)) [**Q**](javascript:Q(662)) [**I**](javascript:I(663)) [**P**](javascript:P(664)) [**Q**](javascript:Q(665)) [**V**](javascript:V(666)) [**S**](javascript:S(667)) [**L**](javascript:L(668)) [**Q**](javascript:Q(669)) [**F**](javascript:F(670)) [**A**](javascript:A(671)) [**G**](javascript:G(672)) [**S**](javascript:S(673)) [**G**](javascript:G(674)) [**N**](javascript:N(675)) [**L**](javascript:L(676)) [**T**](javascript:T(677)) [**L**](javascript:L(678)) [**T**](javascript:T(679)) [**L**](javascript:L(680))

**GGACAGAGGGATACTGTATCAGGAAGTGAACCTGGTGGTGATGAAAGTGACTCAGCCCGA**

[**D**](javascript:D(681)) [**R**](javascript:R(682)) [**G**](javascript:G(683)) [**I**](javascript:I(684)) [**L**](javascript:L(685)) [**Y**](javascript:Y(686)) [**Q**](javascript:Q(687)) [**E**](javascript:E(688)) [**V**](javascript:V(689)) [**N**](javascript:N(690)) [**L**](javascript:L(691)) [**V**](javascript:V(692)) [**V**](javascript:V(693)) [**M**](javascript:M(694)) [**K**](javascript:K(695)) [**V**](javascript:V(696)) [**T**](javascript:T(697)) [**Q**](javascript:Q(698)) [**P**](javascript:P(699)) [**D**](javascript:D(700))

**CAGCAACACTTTGACCTGTGAGGTGATGGGACCCACCTCACCCAAGATGAGACTGATCTT**

[**S**](javascript:S(701)) [**N**](javascript:N(702)) [**T**](javascript:T(703)) [**L**](javascript:L(704)) [**T**](javascript:T(705)) [**C**](javascript:C(706)) [**E**](javascript:E(707)) [**V**](javascript:V(708)) [**M**](javascript:M(709)) [**G**](javascript:G(710)) [**P**](javascript:P(711)) [**T**](javascript:T(712)) [**S**](javascript:S(713)) [**P**](javascript:P(714)) [**K**](javascript:K(715)) [**M**](javascript:M(716)) [**R**](javascript:R(717)) [**L**](javascript:L(718)) [**I**](javascript:I(719)) [**L**](javascript:L(720))

**GAAGCAGGAGAATCAGGAGGCCAGGGTCTCCAGGCAGGAGAAAGTGATTCAAGTGCAGGC**

[**K**](javascript:K(721)) [**Q**](javascript:Q(722)) [**E**](javascript:E(723)) [**N**](javascript:N(724)) [**Q**](javascript:Q(725)) [**E**](javascript:E(726)) [**A**](javascript:A(727)) [**R**](javascript:R(728)) [**V**](javascript:V(729)) [**S**](javascript:S(730)) [**R**](javascript:R(731)) [**Q**](javascript:Q(732)) [**E**](javascript:E(733)) [**K**](javascript:K(734)) [**V**](javascript:V(735)) [**I**](javascript:I(736)) [**Q**](javascript:Q(737)) [**V**](javascript:V(738)) [**Q**](javascript:Q(739)) [**A**](javascript:A(740))

**CCCTGAAGCAGGGGTGTGGCAATGTCTACTGAGTGAAGGTGAAGAGGTCAAGATGGACTC**

[**P**](javascript:P(741)) [**E**](javascript:E(742)) [**A**](javascript:A(743)) [**G**](javascript:G(744)) [**V**](javascript:V(745)) [**W**](javascript:W(746)) [**Q**](javascript:Q(747)) [**C**](javascript:C(748)) [**L**](javascript:L(749)) [**L**](javascript:L(750)) [**S**](javascript:S(751)) [**E**](javascript:E(752)) [**G**](javascript:G(753)) [**E**](javascript:E(754)) [**E**](javascript:E(755)) [**V**](javascript:V(756)) [**K**](javascript:K(757)) [**M**](javascript:M(758)) [**D**](javascript:D(759)) [**S**](javascript:S(760))

**CAAGATCCAGGTTTTATCCAAAGGGTTGAACCAGACAATGTTCCTGGCTGTCGTGCTGGG**

[**K**](javascript:K(761)) [**I**](javascript:I(762)) [**Q**](javascript:Q(763)) [**V**](javascript:V(764)) [**L**](javascript:L(765)) [**S**](javascript:S(766)) [**K**](javascript:K(767)) [**G**](javascript:G(768)) [**L**](javascript:L(769)) [**N**](javascript:N(770)) [**Q**](javascript:Q(771)) [**T**](javascript:T(772)) [**M**](javascript:M(773)) [**F**](javascript:F(774)) [**L**](javascript:L(775)) [**A**](javascript:A(776)) [**V**](javascript:V(777)) [**V**](javascript:V(778)) [**L**](javascript:L(779)) [**G**](javascript:G(780))

**GAGCGCCTTCAGCTTTCTGGTTTTCACGGGGCTCTGCATCCTATTCTGTGTCAGGTGCCG**

[**S**](javascript:S(781)) [**A**](javascript:A(782)) [**F**](javascript:F(783)) [**S**](javascript:S(784)) [**F**](javascript:F(785)) [**L**](javascript:L(786)) [**V**](javascript:V(787)) [**F**](javascript:F(788)) [**T**](javascript:T(789)) [**G**](javascript:G(790)) [**L**](javascript:L(791)) [**C**](javascript:C(792)) [**I**](javascript:I(793)) [**L**](javascript:L(794)) [**F**](javascript:F(795)) [**C**](javascript:C(796)) [**V**](javascript:V(797)) [**R**](javascript:R(798)) [**C**](javascript:C(799)) [**R**](javascript:R(800))

**GCACCAACAGCGCCAGGCAGCACGGATGTCTCAGATCAAGAGGCTTCTCAGTGAGAAGAA**

[**H**](javascript:H(801)) [**Q**](javascript:Q(802)) [**Q**](javascript:Q(803)) [**R**](javascript:R(804)) [**Q**](javascript:Q(805)) [**A**](javascript:A(806)) [**A**](javascript:A(807)) [**R**](javascript:R(808)) [**M**](javascript:M(809)) [**S**](javascript:S(810)) [**Q**](javascript:Q(811)) [**I**](javascript:I(812)) [**K**](javascript:K(813)) [**R**](javascript:R(814)) [**L**](javascript:L(815)) [**L**](javascript:L(816)) [**S**](javascript:S(817)) [**E**](javascript:E(818)) [**K**](javascript:K(819)) [**K**](javascript:K(820))

**GACTTGCCAGTGCTCCCACCGGATGCAGAAAAGCCACAATCTCATATATGGGAGCGATGA**

[**T**](javascript:T(821)) [**C**](javascript:C(822)) [**Q**](javascript:Q(823)) [**C**](javascript:C(824)) [**S**](javascript:S(825)) [**H**](javascript:H(826)) [**R**](javascript:R(827)) [**M**](javascript:M(828)) [**Q**](javascript:Q(829)) [**K**](javascript:K(830)) [**S**](javascript:S(831)) [**H**](javascript:H(832)) [**N**](javascript:N(833)) [**L**](javascript:L(834)) [**I**](javascript:I(835)) [**Y**](javascript:Y(836)) [**G**](javascript:G(837)) [**S**](javascript:S(838)) [**D**](javascript:D(839)) [**D**](javascript:D(840))

**TCCCGTCGTTTTACAACGTCGTGACTGGGAAAACCCTGGCGTTACCCAACTTAATCGCCT**

[**P**](javascript:P(841)) [**V**](javascript:V(842)) [**V**](javascript:V(843)) [**L**](javascript:L(844)) [**Q**](javascript:Q(845)) [**R**](javascript:R(846)) [**R**](javascript:R(847)) [**D**](javascript:D(848)) [**W**](javascript:W(849)) [**E**](javascript:E(850)) [**N**](javascript:N(851)) [**P**](javascript:P(852)) [**G**](javascript:G(853)) [**V**](javascript:V(854)) [**T**](javascript:T(855)) [**Q**](javascript:Q(856)) [**L**](javascript:L(857)) [**N**](javascript:N(858)) [**R**](javascript:R(859)) [**L**](javascript:L(860))

**TGCAGCACATCCCCCTTTCGCCAGCTGGCGTAATAGCGAAGAGGCCCGCACCGATCGCCC**

[**A**](javascript:A(861)) [**A**](javascript:A(862)) [**H**](javascript:H(863)) [**P**](javascript:P(864)) [**P**](javascript:P(865)) [**F**](javascript:F(866)) [**A**](javascript:A(867)) [**S**](javascript:S(868)) [**W**](javascript:W(869)) [**R**](javascript:R(870)) [**N**](javascript:N(871)) [**S**](javascript:S(872)) [**E**](javascript:E(873)) [**E**](javascript:E(874)) [**A**](javascript:A(875)) [**R**](javascript:R(876)) [**T**](javascript:T(877)) [**D**](javascript:D(878)) [**R**](javascript:R(879)) [**P**](javascript:P(880))

**TTCCCAACAGTTGCGCAGCCTGAATGGCGAATGGCGCTTTGCCTGGTTTCCGGCACCAGA**

[**S**](javascript:S(881)) [**Q**](javascript:Q(882)) [**Q**](javascript:Q(883)) [**L**](javascript:L(884)) [**R**](javascript:R(885)) [**S**](javascript:S(886)) [**L**](javascript:L(887)) [**N**](javascript:N(888)) [**G**](javascript:G(889)) [**E**](javascript:E(890)) [**W**](javascript:W(891)) [**R**](javascript:R(892)) [**F**](javascript:F(893)) [**A**](javascript:A(894)) [**W**](javascript:W(895)) [**F**](javascript:F(896)) [**P**](javascript:P(897)) [**A**](javascript:A(898)) [**P**](javascript:P(899)) [**E**](javascript:E(900))

**AGCGGTGCCGGAAAGCTGGCTGGAGTGCGATCTTCCTGAGGCCGATACTGTCGTCGTCCC**

[**A**](javascript:A(901)) [**V**](javascript:V(902)) [**P**](javascript:P(903)) [**E**](javascript:E(904)) [**S**](javascript:S(905)) [**W**](javascript:W(906)) [**L**](javascript:L(907)) [**E**](javascript:E(908)) [**C**](javascript:C(909)) [**D**](javascript:D(910)) [**L**](javascript:L(911)) [**P**](javascript:P(912)) [**E**](javascript:E(913)) [**A**](javascript:A(914)) [**D**](javascript:D(915)) [**T**](javascript:T(916)) [**V**](javascript:V(917)) [**V**](javascript:V(918)) [**V**](javascript:V(919)) [**P**](javascript:P(920))

**CTCAAACTGGCAGATGCACGGTTACGATGCGCCCATCTACACCAACGTAACCTATCCCAT**

[**S**](javascript:S(921)) [**N**](javascript:N(922)) [**W**](javascript:W(923)) [**Q**](javascript:Q(924)) [**M**](javascript:M(925)) [**H**](javascript:H(926)) [**G**](javascript:G(927)) [**Y**](javascript:Y(928)) [**D**](javascript:D(929)) [**A**](javascript:A(930)) [**P**](javascript:P(931)) [**I**](javascript:I(932)) [**Y**](javascript:Y(933)) [**T**](javascript:T(934)) [**N**](javascript:N(935)) [**V**](javascript:V(936)) [**T**](javascript:T(937)) [**Y**](javascript:Y(938)) [**P**](javascript:P(939)) [**I**](javascript:I(940))

**TACGGTCAATCCGCCGTTTGTTCCCACGGAGAATCCGACGGGTTGTTACTCGCTCACATT**

[**T**](javascript:T(941)) [**V**](javascript:V(942)) [**N**](javascript:N(943)) [**P**](javascript:P(944)) [**P**](javascript:P(945)) [**F**](javascript:F(946)) [**V**](javascript:V(947)) [**P**](javascript:P(948)) [**T**](javascript:T(949)) [**E**](javascript:E(950)) [**N**](javascript:N(951)) [**P**](javascript:P(952)) [**T**](javascript:T(953)) [**G**](javascript:G(954)) [**C**](javascript:C(955)) [**Y**](javascript:Y(956)) [**S**](javascript:S(957)) [**L**](javascript:L(958)) [**T**](javascript:T(959)) [**F**](javascript:F(960))

**TAATGTTGATGAAAGCTGGCTACAGGAAGGCCAGACGCGAATTATTTTTGATGGCGTTAA**

[**N**](javascript:N(961)) [**V**](javascript:V(962)) [**D**](javascript:D(963)) [**E**](javascript:E(964)) [**S**](javascript:S(965)) [**W**](javascript:W(966)) [**L**](javascript:L(967)) [**Q**](javascript:Q(968)) [**E**](javascript:E(969)) [**G**](javascript:G(970)) [**Q**](javascript:Q(971)) [**T**](javascript:T(972)) [**R**](javascript:R(973)) [**I**](javascript:I(974)) [**I**](javascript:I(975)) [**F**](javascript:F(976)) [**D**](javascript:D(977)) [**G**](javascript:G(978)) [**V**](javascript:V(979)) [**N**](javascript:N(980))

**CTCGGCGTTTCATCTGTGGTGCAACGGGCGCTGGGTCGGTTACGGCCAGGACAGTCGTTT**

[**S**](javascript:S(981)) [**A**](javascript:A(982)) [**F**](javascript:F(983)) [**H**](javascript:H(984)) [**L**](javascript:L(985)) [**W**](javascript:W(986)) [**C**](javascript:C(987)) [**N**](javascript:N(988)) [**G**](javascript:G(989)) [**R**](javascript:R(990)) [**W**](javascript:W(991)) [**V**](javascript:V(992)) [**G**](javascript:G(993)) [**Y**](javascript:Y(994)) [**G**](javascript:G(995)) [**Q**](javascript:Q(996)) [**D**](javascript:D(997)) [**S**](javascript:S(998)) [**R**](javascript:R(999)) [**L**](javascript:L(1000))

**GCCGTCTGAATTTGACCTGAGCGCATTTTTACGCGCCGGAGAAAACCGCCTCGCGGTGAT**

[**P**](javascript:P(1001)) [**S**](javascript:S(1002)) [**E**](javascript:E(1003)) [**F**](javascript:F(1004)) [**D**](javascript:D(1005)) [**L**](javascript:L(1006)) [**S**](javascript:S(1007)) [**A**](javascript:A(1008)) [**F**](javascript:F(1009)) [**L**](javascript:L(1010)) [**R**](javascript:R(1011)) [**A**](javascript:A(1012)) [**G**](javascript:G(1013)) [**E**](javascript:E(1014)) [**N**](javascript:N(1015)) [**R**](javascript:R(1016)) [**L**](javascript:L(1017)) [**A**](javascript:A(1018)) [**V**](javascript:V(1019)) [**M**](javascript:M(1020))

**GGTGCTGCGTTGGAGTGACGGCAGTTATCTGGAAGATCAGGATATGTGGCGGATGAGCGG**

[**V**](javascript:V(1021)) [**L**](javascript:L(1022)) [**R**](javascript:R(1023)) [**W**](javascript:W(1024)) [**S**](javascript:S(1025)) [**D**](javascript:D(1026)) [**G**](javascript:G(1027)) [**S**](javascript:S(1028)) [**Y**](javascript:Y(1029)) [**L**](javascript:L(1030)) [**E**](javascript:E(1031)) [**D**](javascript:D(1032)) [**Q**](javascript:Q(1033)) [**D**](javascript:D(1034)) [**M**](javascript:M(1035)) [**W**](javascript:W(1036)) [**R**](javascript:R(1037)) [**M**](javascript:M(1038)) [**S**](javascript:S(1039)) [**G**](javascript:G(1040))

**CATTTTCCGTGACGTCTCGTTGCTGCATAAACCGACTACACAAATCAGCGATTTCCATGT**

[**I**](javascript:I(1041)) [**F**](javascript:F(1042)) [**R**](javascript:R(1043)) [**D**](javascript:D(1044)) [**V**](javascript:V(1045)) [**S**](javascript:S(1046)) [**L**](javascript:L(1047)) [**L**](javascript:L(1048)) [**H**](javascript:H(1049)) [**K**](javascript:K(1050)) [**P**](javascript:P(1051)) [**T**](javascript:T(1052)) [**T**](javascript:T(1053)) [**Q**](javascript:Q(1054)) [**I**](javascript:I(1055)) [**S**](javascript:S(1056)) [**D**](javascript:D(1057)) [**F**](javascript:F(1058)) [**H**](javascript:H(1059)) [**V**](javascript:V(1060))

**TGCCACTCGCTTTAATGATGATTTCAGCCGCGCTGTACTGGAGGCTGAAGTTCAGATGTG**

[**A**](javascript:A(1061)) [**T**](javascript:T(1062)) [**R**](javascript:R(1063)) [**F**](javascript:F(1064)) [**N**](javascript:N(1065)) [**D**](javascript:D(1066)) [**D**](javascript:D(1067)) [**F**](javascript:F(1068)) [**S**](javascript:S(1069)) [**R**](javascript:R(1070)) [**A**](javascript:A(1071)) [**V**](javascript:V(1072)) [**L**](javascript:L(1073)) [**E**](javascript:E(1074)) [**A**](javascript:A(1075)) [**E**](javascript:E(1076)) [**V**](javascript:V(1077)) [**Q**](javascript:Q(1078)) [**M**](javascript:M(1079)) [**C**](javascript:C(1080))

**CGGCGAGTTGCGTGACTACCTACGGGTAACAGTTTCTTTATGGCAGGGTGAAACGCAGGT**

[**G**](javascript:G(1081)) [**E**](javascript:E(1082)) [**L**](javascript:L(1083)) [**R**](javascript:R(1084)) [**D**](javascript:D(1085)) [**Y**](javascript:Y(1086)) [**L**](javascript:L(1087)) [**R**](javascript:R(1088)) [**V**](javascript:V(1089)) [**T**](javascript:T(1090)) [**V**](javascript:V(1091)) [**S**](javascript:S(1092)) [**L**](javascript:L(1093)) [**W**](javascript:W(1094)) [**Q**](javascript:Q(1095)) [**G**](javascript:G(1096)) [**E**](javascript:E(1097)) [**T**](javascript:T(1098)) [**Q**](javascript:Q(1099)) [**V**](javascript:V(1100))

**CGCCAGCGGCACCGCGCCTTTCGGCGGTGAAATTATCGATGAGCGTGGTGGTTATGCCGA**

[**A**](javascript:A(1101)) [**S**](javascript:S(1102)) [**G**](javascript:G(1103)) [**T**](javascript:T(1104)) [**A**](javascript:A(1105)) [**P**](javascript:P(1106)) [**F**](javascript:F(1107)) [**G**](javascript:G(1108)) [**G**](javascript:G(1109)) [**E**](javascript:E(1110)) [**I**](javascript:I(1111)) [**I**](javascript:I(1112)) [**D**](javascript:D(1113)) [**E**](javascript:E(1114)) [**R**](javascript:R(1115)) [**G**](javascript:G(1116)) [**G**](javascript:G(1117)) [**Y**](javascript:Y(1118)) [**A**](javascript:A(1119)) [**D**](javascript:D(1120))

**TCGCGTCACACTACGTCTGAACGTCGAAAACCCGAAACTGTGGAGCGCCGAAATCCCGAA**

[**R**](javascript:R(1121)) [**V**](javascript:V(1122)) [**T**](javascript:T(1123)) [**L**](javascript:L(1124)) [**R**](javascript:R(1125)) [**L**](javascript:L(1126)) [**N**](javascript:N(1127)) [**V**](javascript:V(1128)) [**E**](javascript:E(1129)) [**N**](javascript:N(1130)) [**P**](javascript:P(1131)) [**K**](javascript:K(1132)) [**L**](javascript:L(1133)) [**W**](javascript:W(1134)) [**S**](javascript:S(1135)) [**A**](javascript:A(1136)) [**E**](javascript:E(1137)) [**I**](javascript:I(1138)) [**P**](javascript:P(1139)) [**N**](javascript:N(1140))

**TCTCTATCGTGCGGTGGTTGAACTGCACACCGCCGACGGCACGCTGATTGAAGCAGAAGC**

[**L**](javascript:L(1141)) [**Y**](javascript:Y(1142)) [**R**](javascript:R(1143)) [**A**](javascript:A(1144)) [**V**](javascript:V(1145)) [**V**](javascript:V(1146)) [**E**](javascript:E(1147)) [**L**](javascript:L(1148)) [**H**](javascript:H(1149)) [**T**](javascript:T(1150)) [**A**](javascript:A(1151)) [**D**](javascript:D(1152)) [**G**](javascript:G(1153)) [**T**](javascript:T(1154)) [**L**](javascript:L(1155)) [**I**](javascript:I(1156)) [**E**](javascript:E(1157)) [**A**](javascript:A(1158)) [**E**](javascript:E(1159)) [**A**](javascript:A(1160))

**CTGCGATGTCGGTTTCCGCGAGGTGCGGATTGAAAATGGTCTGCTGCTGCTGAACGGCAA**

[**C**](javascript:C(1161)) [**D**](javascript:D(1162)) [**V**](javascript:V(1163)) [**G**](javascript:G(1164)) [**F**](javascript:F(1165)) [**R**](javascript:R(1166)) [**E**](javascript:E(1167)) [**V**](javascript:V(1168)) [**R**](javascript:R(1169)) [**I**](javascript:I(1170)) [**E**](javascript:E(1171)) [**N**](javascript:N(1172)) [**G**](javascript:G(1173)) [**L**](javascript:L(1174)) [**L**](javascript:L(1175)) [**L**](javascript:L(1176)) [**L**](javascript:L(1177)) [**N**](javascript:N(1178)) [**G**](javascript:G(1179)) [**K**](javascript:K(1180))

**GCCGTTGCTGATTCGAGGCGTTAACCGTCACGAGCATCATCCTCTGCATGGTCAGGTCAT**

[**P**](javascript:P(1181)) [**L**](javascript:L(1182)) [**L**](javascript:L(1183)) [**I**](javascript:I(1184)) [**R**](javascript:R(1185)) [**G**](javascript:G(1186)) [**V**](javascript:V(1187)) [**N**](javascript:N(1188)) [**R**](javascript:R(1189)) [**H**](javascript:H(1190)) [**E**](javascript:E(1191)) [**H**](javascript:H(1192)) [**H**](javascript:H(1193)) [**P**](javascript:P(1194)) [**L**](javascript:L(1195)) [**H**](javascript:H(1196)) [**G**](javascript:G(1197)) [**Q**](javascript:Q(1198)) [**V**](javascript:V(1199)) [**M**](javascript:M(1200))

**GGATGAGCAGACGATGGTGCAGGATATCCTGCTGATGAAGCAGAACAACTTTAACGCCGT**

[**D**](javascript:D(1201)) [**E**](javascript:E(1202)) [**Q**](javascript:Q(1203)) [**T**](javascript:T(1204)) [**M**](javascript:M(1205)) [**V**](javascript:V(1206)) [**Q**](javascript:Q(1207)) [**D**](javascript:D(1208)) [**I**](javascript:I(1209)) [**L**](javascript:L(1210)) [**L**](javascript:L(1211)) [**M**](javascript:M(1212)) [**K**](javascript:K(1213)) [**Q**](javascript:Q(1214)) [**N**](javascript:N(1215)) [**N**](javascript:N(1216)) [**F**](javascript:F(1217)) [**N**](javascript:N(1218)) [**A**](javascript:A(1219)) [**V**](javascript:V(1220))

**GCGCTGTTCGCATTATCCGAACCATCCGCTGTGGTACACGCTGTGCGACCGCTACGGCCT**

[**R**](javascript:R(1221)) [**C**](javascript:C(1222)) [**S**](javascript:S(1223)) [**H**](javascript:H(1224)) [**Y**](javascript:Y(1225)) [**P**](javascript:P(1226)) [**N**](javascript:N(1227)) [**H**](javascript:H(1228)) [**P**](javascript:P(1229)) [**L**](javascript:L(1230)) [**W**](javascript:W(1231)) [**Y**](javascript:Y(1232)) [**T**](javascript:T(1233)) [**L**](javascript:L(1234)) [**C**](javascript:C(1235)) [**D**](javascript:D(1236)) [**R**](javascript:R(1237)) [**Y**](javascript:Y(1238)) [**G**](javascript:G(1239)) [**L**](javascript:L(1240))

**GTATGTGGTGGATGAAGCCAATATTGAAACCCACGGCATGGTGCCAATGAATCGTCTGAC**

[**Y**](javascript:Y(1241)) [**V**](javascript:V(1242)) [**V**](javascript:V(1243)) [**D**](javascript:D(1244)) [**E**](javascript:E(1245)) [**A**](javascript:A(1246)) [**N**](javascript:N(1247)) [**I**](javascript:I(1248)) [**E**](javascript:E(1249)) [**T**](javascript:T(1250)) [**H**](javascript:H(1251)) [**G**](javascript:G(1252)) [**M**](javascript:M(1253)) [**V**](javascript:V(1254)) [**P**](javascript:P(1255)) [**M**](javascript:M(1256)) [**N**](javascript:N(1257)) [**R**](javascript:R(1258)) [**L**](javascript:L(1259)) [**T**](javascript:T(1260))

**CGATGATCCGCGCTGGCTACCGGCGATGAGCGAACGCGTAACGCGAATGGTGCAGCGCGA**

[**D**](javascript:D(1261)) [**D**](javascript:D(1262)) [**P**](javascript:P(1263)) [**R**](javascript:R(1264)) [**W**](javascript:W(1265)) [**L**](javascript:L(1266)) [**P**](javascript:P(1267)) [**A**](javascript:A(1268)) [**M**](javascript:M(1269)) [**S**](javascript:S(1270)) [**E**](javascript:E(1271)) [**R**](javascript:R(1272)) [**V**](javascript:V(1273)) [**T**](javascript:T(1274)) [**R**](javascript:R(1275)) [**M**](javascript:M(1276)) [**V**](javascript:V(1277)) [**Q**](javascript:Q(1278)) [**R**](javascript:R(1279)) [**D**](javascript:D(1280))

**TCGTAATCACCCGAGTGTGATCATCTGGTCGCTGGGGAATGAATCAGGCCACGGCGCTAA**

[**R**](javascript:R(1281)) [**N**](javascript:N(1282)) [**H**](javascript:H(1283)) [**P**](javascript:P(1284)) [**S**](javascript:S(1285)) [**V**](javascript:V(1286)) [**I**](javascript:I(1287)) [**I**](javascript:I(1288)) [**W**](javascript:W(1289)) [**S**](javascript:S(1290)) [**L**](javascript:L(1291)) [**G**](javascript:G(1292)) [**N**](javascript:N(1293)) [**E**](javascript:E(1294)) [**S**](javascript:S(1295)) [**G**](javascript:G(1296)) [**H**](javascript:H(1297)) [**G**](javascript:G(1298)) [**A**](javascript:A(1299)) [**N**](javascript:N(1300))

**TCACGACGCGCTGTATCGCTGGATCAAATCTGTCGATCCTTCCCGCCCGGTGCAGTATGA**

[**H**](javascript:H(1301)) [**D**](javascript:D(1302)) [**A**](javascript:A(1303)) [**L**](javascript:L(1304)) [**Y**](javascript:Y(1305)) [**R**](javascript:R(1306)) [**W**](javascript:W(1307)) [**I**](javascript:I(1308)) [**K**](javascript:K(1309)) [**S**](javascript:S(1310)) [**V**](javascript:V(1311)) [**D**](javascript:D(1312)) [**P**](javascript:P(1313)) [**S**](javascript:S(1314)) [**R**](javascript:R(1315)) [**P**](javascript:P(1316)) [**V**](javascript:V(1317)) [**Q**](javascript:Q(1318)) [**Y**](javascript:Y(1319)) [**E**](javascript:E(1320))

**AGGCGGCGGAGCCGACACCACGGCCACCGATATTATTTGCCCGATGTACGCGCGCGTGGA**

[**G**](javascript:G(1321)) [**G**](javascript:G(1322)) [**G**](javascript:G(1323)) [**A**](javascript:A(1324)) [**D**](javascript:D(1325)) [**T**](javascript:T(1326)) [**T**](javascript:T(1327)) [**A**](javascript:A(1328)) [**T**](javascript:T(1329)) [**D**](javascript:D(1330)) [**I**](javascript:I(1331)) [**I**](javascript:I(1332)) [**C**](javascript:C(1333)) [**P**](javascript:P(1334)) [**M**](javascript:M(1335)) [**Y**](javascript:Y(1336)) [**A**](javascript:A(1337)) [**R**](javascript:R(1338)) [**V**](javascript:V(1339)) [**D**](javascript:D(1340))

**TGAAGACCAGCCCTTCCCGGCTGTGCCGAAATGGTCCATCAAAAAATGGCTTTCGCTACC**

[**E**](javascript:E(1341)) [**D**](javascript:D(1342)) [**Q**](javascript:Q(1343)) [**P**](javascript:P(1344)) [**F**](javascript:F(1345)) [**P**](javascript:P(1346)) [**A**](javascript:A(1347)) [**V**](javascript:V(1348)) [**P**](javascript:P(1349)) [**K**](javascript:K(1350)) [**W**](javascript:W(1351)) [**S**](javascript:S(1352)) [**I**](javascript:I(1353)) [**K**](javascript:K(1354)) [**K**](javascript:K(1355)) [**W**](javascript:W(1356)) [**L**](javascript:L(1357)) [**S**](javascript:S(1358)) [**L**](javascript:L(1359)) [**P**](javascript:P(1360))

**TGGAGAGACGCGCCCGCTGATCCTTTGCGAATACGCCCACGCGATGGGTAACAGTCTTGG**

[**G**](javascript:G(1361)) [**E**](javascript:E(1362)) [**T**](javascript:T(1363)) [**R**](javascript:R(1364)) [**P**](javascript:P(1365)) [**L**](javascript:L(1366)) [**I**](javascript:I(1367)) [**L**](javascript:L(1368)) [**C**](javascript:C(1369)) [**E**](javascript:E(1370)) [**Y**](javascript:Y(1371)) [**A**](javascript:A(1372)) [**H**](javascript:H(1373)) [**A**](javascript:A(1374)) [**M**](javascript:M(1375)) [**G**](javascript:G(1376)) [**N**](javascript:N(1377)) [**S**](javascript:S(1378)) [**L**](javascript:L(1379)) [**G**](javascript:G(1380))

**CGGTTTCGCTAAATACTGGCAGGCGTTTCGTCAGTATCCCCGTTTACAGGGCGGCTTCGT**

[**G**](javascript:G(1381)) [**F**](javascript:F(1382)) [**A**](javascript:A(1383)) [**K**](javascript:K(1384)) [**Y**](javascript:Y(1385)) [**W**](javascript:W(1386)) [**Q**](javascript:Q(1387)) [**A**](javascript:A(1388)) [**F**](javascript:F(1389)) [**R**](javascript:R(1390)) [**Q**](javascript:Q(1391)) [**Y**](javascript:Y(1392)) [**P**](javascript:P(1393)) [**R**](javascript:R(1394)) [**L**](javascript:L(1395)) [**Q**](javascript:Q(1396)) [**G**](javascript:G(1397)) [**G**](javascript:G(1398)) [**F**](javascript:F(1399)) [**V**](javascript:V(1400))

**CTGGGACTGGGTGGATCAGTCGCTGATTAAATATGATGAAAACGGCAACCCGTGGTCGGC**

[**W**](javascript:W(1401)) [**D**](javascript:D(1402)) [**W**](javascript:W(1403)) [**V**](javascript:V(1404)) [**D**](javascript:D(1405)) [**Q**](javascript:Q(1406)) [**S**](javascript:S(1407)) [**L**](javascript:L(1408)) [**I**](javascript:I(1409)) [**K**](javascript:K(1410)) [**Y**](javascript:Y(1411)) [**D**](javascript:D(1412)) [**E**](javascript:E(1413)) [**N**](javascript:N(1414)) [**G**](javascript:G(1415)) [**N**](javascript:N(1416)) [**P**](javascript:P(1417)) [**W**](javascript:W(1418)) [**S**](javascript:S(1419)) [**A**](javascript:A(1420))

**TTACGGCGGTGATTTTGGCGATACGCCGAACGATCGCCAGTTCTGTATGAACGGTCTGGT**

[**Y**](javascript:Y(1421)) [**G**](javascript:G(1422)) [**G**](javascript:G(1423)) [**D**](javascript:D(1424)) [**F**](javascript:F(1425)) [**G**](javascript:G(1426)) [**D**](javascript:D(1427)) [**T**](javascript:T(1428)) [**P**](javascript:P(1429)) [**N**](javascript:N(1430)) [**D**](javascript:D(1431)) [**R**](javascript:R(1432)) [**Q**](javascript:Q(1433)) [**F**](javascript:F(1434)) [**C**](javascript:C(1435)) [**M**](javascript:M(1436)) [**N**](javascript:N(1437)) [**G**](javascript:G(1438)) [**L**](javascript:L(1439)) [**V**](javascript:V(1440))

**CTTTGCCGACCGCACGCCGCATCCAGCGCTGACGGAAGCAAAACACCAGCAGCAGTTTTT**

[**F**](javascript:F(1441)) [**A**](javascript:A(1442)) [**D**](javascript:D(1443)) [**R**](javascript:R(1444)) [**T**](javascript:T(1445)) [**P**](javascript:P(1446)) [**H**](javascript:H(1447)) [**P**](javascript:P(1448)) [**A**](javascript:A(1449)) [**L**](javascript:L(1450)) [**T**](javascript:T(1451)) [**E**](javascript:E(1452)) [**A**](javascript:A(1453)) [**K**](javascript:K(1454)) [**H**](javascript:H(1455)) [**Q**](javascript:Q(1456)) [**Q**](javascript:Q(1457)) [**Q**](javascript:Q(1458)) [**F**](javascript:F(1459)) [**F**](javascript:F(1460))

**CCAGTTCCGTTTATCCGGGCAAACCATCGAAGTGACCAGCGAATACCTGTTCCGTCATAG**

[**Q**](javascript:Q(1461)) [**F**](javascript:F(1462)) [**R**](javascript:R(1463)) [**L**](javascript:L(1464)) [**S**](javascript:S(1465)) [**G**](javascript:G(1466)) [**Q**](javascript:Q(1467)) [**T**](javascript:T(1468)) [**I**](javascript:I(1469)) [**E**](javascript:E(1470)) [**V**](javascript:V(1471)) [**T**](javascript:T(1472)) [**S**](javascript:S(1473)) [**E**](javascript:E(1474)) [**Y**](javascript:Y(1475)) [**L**](javascript:L(1476)) [**F**](javascript:F(1477)) [**R**](javascript:R(1478)) [**H**](javascript:H(1479)) [**S**](javascript:S(1480))

**CGATAACGAGCTCCTGCACTGGATGGTGGCGCTGGATGGTAAGCCGCTGGCAAGCGGTGA**

[**D**](javascript:D(1481)) [**N**](javascript:N(1482)) [**E**](javascript:E(1483)) [**L**](javascript:L(1484)) [**L**](javascript:L(1485)) [**H**](javascript:H(1486)) [**W**](javascript:W(1487)) [**M**](javascript:M(1488)) [**V**](javascript:V(1489)) [**A**](javascript:A(1490)) [**L**](javascript:L(1491)) [**D**](javascript:D(1492)) [**G**](javascript:G(1493)) [**K**](javascript:K(1494)) [**P**](javascript:P(1495)) [**L**](javascript:L(1496)) [**A**](javascript:A(1497)) [**S**](javascript:S(1498)) [**G**](javascript:G(1499)) [**E**](javascript:E(1500))

**AGTGCCTCTGGATGTCGCTCCACAAGGTAAACAGTTGATTGAACTGCCTGAACTACCGCA**

[**V**](javascript:V(1501)) [**P**](javascript:P(1502)) [**L**](javascript:L(1503)) [**D**](javascript:D(1504)) [**V**](javascript:V(1505)) [**A**](javascript:A(1506)) [**P**](javascript:P(1507)) [**Q**](javascript:Q(1508)) [**G**](javascript:G(1509)) [**K**](javascript:K(1510)) [**Q**](javascript:Q(1511)) [**L**](javascript:L(1512)) [**I**](javascript:I(1513)) [**E**](javascript:E(1514)) [**L**](javascript:L(1515)) [**P**](javascript:P(1516)) [**E**](javascript:E(1517)) [**L**](javascript:L(1518)) [**P**](javascript:P(1519)) [**Q**](javascript:Q(1520))

**GCCGGAGAGCGCCGGGCAACTCTGGCTCACAGTACGCGTAGTGCAACCGAACGCGACCGC**

[**P**](javascript:P(1521)) [**E**](javascript:E(1522)) [**S**](javascript:S(1523)) [**A**](javascript:A(1524)) [**G**](javascript:G(1525)) [**Q**](javascript:Q(1526)) [**L**](javascript:L(1527)) [**W**](javascript:W(1528)) [**L**](javascript:L(1529)) [**T**](javascript:T(1530)) [**V**](javascript:V(1531)) [**R**](javascript:R(1532)) [**V**](javascript:V(1533)) [**V**](javascript:V(1534)) [**Q**](javascript:Q(1535)) [**P**](javascript:P(1536)) [**N**](javascript:N(1537)) [**A**](javascript:A(1538)) [**T**](javascript:T(1539)) [**A**](javascript:A(1540))

**ATGGTCAGAAGCCGGGCACATCAGCGCCTGGCAGCAGTGGCGTCTGGCGGAAAACCTCAG**

[**W**](javascript:W(1541)) [**S**](javascript:S(1542)) [**E**](javascript:E(1543)) [**A**](javascript:A(1544)) [**G**](javascript:G(1545)) [**H**](javascript:H(1546)) [**I**](javascript:I(1547)) [**S**](javascript:S(1548)) [**A**](javascript:A(1549)) [**W**](javascript:W(1550)) [**Q**](javascript:Q(1551)) [**Q**](javascript:Q(1552)) [**W**](javascript:W(1553)) [**R**](javascript:R(1554)) [**L**](javascript:L(1555)) [**A**](javascript:A(1556)) [**E**](javascript:E(1557)) [**N**](javascript:N(1558)) [**L**](javascript:L(1559)) [**S**](javascript:S(1560))

**TGTGACGCTCCCCGCCGCGTCCCACGCCATCCCGCATCTGACCACCAGCGAAATGGATTT**

[**V**](javascript:V(1561)) [**T**](javascript:T(1562)) [**L**](javascript:L(1563)) [**P**](javascript:P(1564)) [**A**](javascript:A(1565)) [**A**](javascript:A(1566)) [**S**](javascript:S(1567)) [**H**](javascript:H(1568)) [**A**](javascript:A(1569)) [**I**](javascript:I(1570)) [**P**](javascript:P(1571)) [**H**](javascript:H(1572)) [**L**](javascript:L(1573)) [**T**](javascript:T(1574)) [**T**](javascript:T(1575)) [**S**](javascript:S(1576)) [**E**](javascript:E(1577)) [**M**](javascript:M(1578)) [**D**](javascript:D(1579)) [**F**](javascript:F(1580))

**TTGCATCGAGCTGGGTAATAAGCGTTGGCAATTTAACCGCCAGTCAGGCTTTCTTTCACA**

[**C**](javascript:C(1581)) [**I**](javascript:I(1582)) [**E**](javascript:E(1583)) [**L**](javascript:L(1584)) [**G**](javascript:G(1585)) [**N**](javascript:N(1586)) [**K**](javascript:K(1587)) [**R**](javascript:R(1588)) [**W**](javascript:W(1589)) [**Q**](javascript:Q(1590)) [**F**](javascript:F(1591)) [**N**](javascript:N(1592)) [**R**](javascript:R(1593)) [**Q**](javascript:Q(1594)) [**S**](javascript:S(1595)) [**G**](javascript:G(1596)) [**F**](javascript:F(1597)) [**L**](javascript:L(1598)) [**S**](javascript:S(1599)) [**Q**](javascript:Q(1600))

**GATGTGGATTGGCGATAAAAAACAACTGCTGACGCCGCTGCGCGATCAGTTCACCCGTGC**

[**M**](javascript:M(1601)) [**W**](javascript:W(1602)) [**I**](javascript:I(1603)) [**G**](javascript:G(1604)) [**D**](javascript:D(1605)) [**K**](javascript:K(1606)) [**K**](javascript:K(1607)) [**Q**](javascript:Q(1608)) [**L**](javascript:L(1609)) [**L**](javascript:L(1610)) [**T**](javascript:T(1611)) [**P**](javascript:P(1612)) [**L**](javascript:L(1613)) [**R**](javascript:R(1614)) [**D**](javascript:D(1615)) [**Q**](javascript:Q(1616)) [**F**](javascript:F(1617)) [**T**](javascript:T(1618)) [**R**](javascript:R(1619)) [**A**](javascript:A(1620))

**ACCGCTGGATAACGACATTGGCGTAAGTGAAGCGACCCGCATTGACCCTAACGCCTGGGT**

[**P**](javascript:P(1621)) [**L**](javascript:L(1622)) [**D**](javascript:D(1623)) [**N**](javascript:N(1624)) [**D**](javascript:D(1625)) [**I**](javascript:I(1626)) [**G**](javascript:G(1627)) [**V**](javascript:V(1628)) [**S**](javascript:S(1629)) [**E**](javascript:E(1630)) [**A**](javascript:A(1631)) [**T**](javascript:T(1632)) [**R**](javascript:R(1633)) [**I**](javascript:I(1634)) [**D**](javascript:D(1635)) [**P**](javascript:P(1636)) [**N**](javascript:N(1637)) [**A**](javascript:A(1638)) [**W**](javascript:W(1639)) [**V**](javascript:V(1640))

**CGAACGCTGGAAGGCGGCGGGCCATTACCAGGCCGAAGCAGCGTTGTTGCAGTGCACGGC**

[**E**](javascript:E(1641)) [**R**](javascript:R(1642)) [**W**](javascript:W(1643)) [**K**](javascript:K(1644)) [**A**](javascript:A(1645)) [**A**](javascript:A(1646)) [**G**](javascript:G(1647)) [**H**](javascript:H(1648)) [**Y**](javascript:Y(1649)) [**Q**](javascript:Q(1650)) [**A**](javascript:A(1651)) [**E**](javascript:E(1652)) [**A**](javascript:A(1653)) [**A**](javascript:A(1654)) [**L**](javascript:L(1655)) [**L**](javascript:L(1656)) [**Q**](javascript:Q(1657)) [**C**](javascript:C(1658)) [**T**](javascript:T(1659)) [**A**](javascript:A(1660))

**AGATACACTTGCTGATGCGGTGCTGATTACGACCGCTCACGCGTGGCAGCATCAGGGGAA**

[**D**](javascript:D(1661)) [**T**](javascript:T(1662)) [**L**](javascript:L(1663)) [**A**](javascript:A(1664)) [**D**](javascript:D(1665)) [**A**](javascript:A(1666)) [**V**](javascript:V(1667)) [**L**](javascript:L(1668)) [**I**](javascript:I(1669)) [**T**](javascript:T(1670)) [**T**](javascript:T(1671)) [**A**](javascript:A(1672)) [**H**](javascript:H(1673)) [**A**](javascript:A(1674)) [**W**](javascript:W(1675)) [**Q**](javascript:Q(1676)) [**H**](javascript:H(1677)) [**Q**](javascript:Q(1678)) [**G**](javascript:G(1679)) [**K**](javascript:K(1680))

**AACCTTATTTATCAGCCGGAAAACCTACCGGATTGATGGTAGTGGTCAAATGGCGATTAC**

[**T**](javascript:T(1681)) [**L**](javascript:L(1682)) [**F**](javascript:F(1683)) [**I**](javascript:I(1684)) [**S**](javascript:S(1685)) [**R**](javascript:R(1686)) [**K**](javascript:K(1687)) [**T**](javascript:T(1688)) [**Y**](javascript:Y(1689)) [**R**](javascript:R(1690)) [**I**](javascript:I(1691)) [**D**](javascript:D(1692)) [**G**](javascript:G(1693)) [**S**](javascript:S(1694)) [**G**](javascript:G(1695)) [**Q**](javascript:Q(1696)) [**M**](javascript:M(1697)) [**A**](javascript:A(1698)) [**I**](javascript:I(1699)) [**T**](javascript:T(1700))

**CGTTGATGTTGAAGTGGCGAGCGATACACCGCATCCGGCGCGGATTGGCCTGAACTGCCA**

[**V**](javascript:V(1701)) [**D**](javascript:D(1702)) [**V**](javascript:V(1703)) [**E**](javascript:E(1704)) [**V**](javascript:V(1705)) [**A**](javascript:A(1706)) [**S**](javascript:S(1707)) [**D**](javascript:D(1708)) [**T**](javascript:T(1709)) [**P**](javascript:P(1710)) [**H**](javascript:H(1711)) [**P**](javascript:P(1712)) [**A**](javascript:A(1713)) [**R**](javascript:R(1714)) [**I**](javascript:I(1715)) [**G**](javascript:G(1716)) [**L**](javascript:L(1717)) [**N**](javascript:N(1718)) [**C**](javascript:C(1719)) [**Q**](javascript:Q(1720))

**GCTGGCGCAGGTAGCAGAGCGGGTAAACTGGCTCGGATTAGGGCCGCAAGAAAACTATCC**

[**L**](javascript:L(1721)) [**A**](javascript:A(1722)) [**Q**](javascript:Q(1723)) [**V**](javascript:V(1724)) [**A**](javascript:A(1725)) [**E**](javascript:E(1726)) [**R**](javascript:R(1727)) [**V**](javascript:V(1728)) [**N**](javascript:N(1729)) [**W**](javascript:W(1730)) [**L**](javascript:L(1731)) [**G**](javascript:G(1732)) [**L**](javascript:L(1733)) [**G**](javascript:G(1734)) [**P**](javascript:P(1735)) [**Q**](javascript:Q(1736)) [**E**](javascript:E(1737)) [**N**](javascript:N(1738)) [**Y**](javascript:Y(1739)) [**P**](javascript:P(1740))

**CGACCGCCTTACTGCCGCCTGTTTTGACCGCTGGGATCTGCCATTGTCAGACATGTATAC**

[**D**](javascript:D(1741)) [**R**](javascript:R(1742)) [**L**](javascript:L(1743)) [**T**](javascript:T(1744)) [**A**](javascript:A(1745)) [**A**](javascript:A(1746)) [**C**](javascript:C(1747)) [**F**](javascript:F(1748)) [**D**](javascript:D(1749)) [**R**](javascript:R(1750)) [**W**](javascript:W(1751)) [**D**](javascript:D(1752)) [**L**](javascript:L(1753)) [**P**](javascript:P(1754)) [**L**](javascript:L(1755)) [**S**](javascript:S(1756)) [**D**](javascript:D(1757)) [**M**](javascript:M(1758)) [**Y**](javascript:Y(1759)) [**T**](javascript:T(1760))

**CCCGTACGTCTTCCCGAGCGAAAACGGTCTGCGCTGCGGGACGCGCGAATTGAATTATGG**

[**P**](javascript:P(1761)) [**Y**](javascript:Y(1762)) [**V**](javascript:V(1763)) [**F**](javascript:F(1764)) [**P**](javascript:P(1765)) [**S**](javascript:S(1766)) [**E**](javascript:E(1767)) [**N**](javascript:N(1768)) [**G**](javascript:G(1769)) [**L**](javascript:L(1770)) [**R**](javascript:R(1771)) [**C**](javascript:C(1772)) [**G**](javascript:G(1773)) [**T**](javascript:T(1774)) [**R**](javascript:R(1775)) [**E**](javascript:E(1776)) [**L**](javascript:L(1777)) [**N**](javascript:N(1778)) [**Y**](javascript:Y(1779)) [**G**](javascript:G(1780))

**CCCACACCAGTGGCGCGGCGACTTCCAGTTCAACATCAGCCGCTACAGTCAACAGCAACT**

[**P**](javascript:P(1781)) [**H**](javascript:H(1782)) [**Q**](javascript:Q(1783)) [**W**](javascript:W(1784)) [**R**](javascript:R(1785)) [**G**](javascript:G(1786)) [**D**](javascript:D(1787)) [**F**](javascript:F(1788)) [**Q**](javascript:Q(1789)) [**F**](javascript:F(1790)) [**N**](javascript:N(1791)) [**I**](javascript:I(1792)) [**S**](javascript:S(1793)) [**R**](javascript:R(1794)) [**Y**](javascript:Y(1795)) [**S**](javascript:S(1796)) [**Q**](javascript:Q(1797)) [**Q**](javascript:Q(1798)) [**Q**](javascript:Q(1799)) [**L**](javascript:L(1800))

**GATGGAAACCAGCCATCGCCATCTGCTGCACGCGGAAGAAGGCACATGGCTGAATATCGA**

[**M**](javascript:M(1801)) [**E**](javascript:E(1802)) [**T**](javascript:T(1803)) [**S**](javascript:S(1804)) [**H**](javascript:H(1805)) [**R**](javascript:R(1806)) [**H**](javascript:H(1807)) [**L**](javascript:L(1808)) [**L**](javascript:L(1809)) [**H**](javascript:H(1810)) [**A**](javascript:A(1811)) [**E**](javascript:E(1812)) [**E**](javascript:E(1813)) [**G**](javascript:G(1814)) [**T**](javascript:T(1815)) [**W**](javascript:W(1816)) [**L**](javascript:L(1817)) [**N**](javascript:N(1818)) [**I**](javascript:I(1819)) [**D**](javascript:D(1820))

**CGGTTTCCATATGGGGATTGGTGGCGACGACTCCTGGAGCCCGTCAGTATCGGCGGAATT**

[**G**](javascript:G(1821)) [**F**](javascript:F(1822)) [**H**](javascript:H(1823)) [**M**](javascript:M(1824)) [**G**](javascript:G(1825)) [**I**](javascript:I(1826)) [**G**](javascript:G(1827)) [**G**](javascript:G(1828)) [**D**](javascript:D(1829)) [**D**](javascript:D(1830)) [**S**](javascript:S(1831)) [**W**](javascript:W(1832)) [**S**](javascript:S(1833)) [**P**](javascript:P(1834)) [**S**](javascript:S(1835)) [**V**](javascript:V(1836)) [**S**](javascript:S(1837)) [**A**](javascript:A(1838)) [**E**](javascript:E(1839)) [**F**](javascript:F(1840))

**CCAGCTGAGCGCCGGTCGCTACCATTACCAGTTGGTCTGGTGTCAGGGGATCCCCCGGGC**

[**Q**](javascript:Q(1841)) [**L**](javascript:L(1842)) [**S**](javascript:S(1843)) [**A**](javascript:A(1844)) [**G**](javascript:G(1845)) [**R**](javascript:R(1846)) [**Y**](javascript:Y(1847)) [**H**](javascript:H(1848)) [**Y**](javascript:Y(1849)) [**Q**](javascript:Q(1850)) [**L**](javascript:L(1851)) [**V**](javascript:V(1852)) [**W**](javascript:W(1853)) [**C**](javascript:C(1854)) [**Q**](javascript:Q(1855)) [**G**](javascript:G(1856)) [**I**](javascript:I(1857)) [**P**](javascript:P(1858)) [**R**](javascript:R(1859)) [**A**](javascript:A(1860))

**TGCAGCCAATATGGGATCGGCCATTGAACAAGATGGATTGCACGCAGGTTCTCCGGCCGC**

[**A**](javascript:A(1861)) [**A**](javascript:A(1862)) [**N**](javascript:N(1863)) [**M**](javascript:M(1864)) [**G**](javascript:G(1865)) [**S**](javascript:S(1866)) [**A**](javascript:A(1867)) [**I**](javascript:I(1868)) [**E**](javascript:E(1869)) [**Q**](javascript:Q(1870)) [**D**](javascript:D(1871)) [**G**](javascript:G(1872)) [**L**](javascript:L(1873)) [**H**](javascript:H(1874)) [**A**](javascript:A(1875)) [**G**](javascript:G(1876)) [**S**](javascript:S(1877)) [**P**](javascript:P(1878)) [**A**](javascript:A(1879)) [**A**](javascript:A(1880))

**TTGGGTGGAGAGGCTATTCGGCTATGACTGGGCACAACAGACAATCGGCTGCTCTGATGC**

[**W**](javascript:W(1881)) [**V**](javascript:V(1882)) [**E**](javascript:E(1883)) [**R**](javascript:R(1884)) [**L**](javascript:L(1885)) [**F**](javascript:F(1886)) [**G**](javascript:G(1887)) [**Y**](javascript:Y(1888)) [**D**](javascript:D(1889)) [**W**](javascript:W(1890)) [**A**](javascript:A(1891)) [**Q**](javascript:Q(1892)) [**Q**](javascript:Q(1893)) [**T**](javascript:T(1894)) [**I**](javascript:I(1895)) [**G**](javascript:G(1896)) [**C**](javascript:C(1897)) [**S**](javascript:S(1898)) [**D**](javascript:D(1899)) [**A**](javascript:A(1900))

**CGCCGTGTTCCGGCTGTCAGCGCAGGGGCGCCCGGTTCTTTTTGTCAAGACCGACCTGTC**

[**A**](javascript:A(1901)) [**V**](javascript:V(1902)) [**F**](javascript:F(1903)) [**R**](javascript:R(1904)) [**L**](javascript:L(1905)) [**S**](javascript:S(1906)) [**A**](javascript:A(1907)) [**Q**](javascript:Q(1908)) [**G**](javascript:G(1909)) [**R**](javascript:R(1910)) [**P**](javascript:P(1911)) [**V**](javascript:V(1912)) [**L**](javascript:L(1913)) [**F**](javascript:F(1914)) [**V**](javascript:V(1915)) [**K**](javascript:K(1916)) [**T**](javascript:T(1917)) [**D**](javascript:D(1918)) [**L**](javascript:L(1919)) [**S**](javascript:S(1920))

**CGGTGCCCTGAATGAACTGCAGGACGAGGCAGCGCGGCTATCGTGGCTGGCCACGACGGG**

[**G**](javascript:G(1921)) [**A**](javascript:A(1922)) [**L**](javascript:L(1923)) [**N**](javascript:N(1924)) [**E**](javascript:E(1925)) [**L**](javascript:L(1926)) [**Q**](javascript:Q(1927)) [**D**](javascript:D(1928)) [**E**](javascript:E(1929)) [**A**](javascript:A(1930)) [**A**](javascript:A(1931)) [**R**](javascript:R(1932)) [**L**](javascript:L(1933)) [**S**](javascript:S(1934)) [**W**](javascript:W(1935)) [**L**](javascript:L(1936)) [**A**](javascript:A(1937)) [**T**](javascript:T(1938)) [**T**](javascript:T(1939)) [**G**](javascript:G(1940))

**CGTTCCTTGCGCAGCTGTGCTCGACGTTGTCACTGAAGCGGGAAGGGACTGGCTGCTATT**

[**V**](javascript:V(1941)) [**P**](javascript:P(1942)) [**C**](javascript:C(1943)) [**A**](javascript:A(1944)) [**A**](javascript:A(1945)) [**V**](javascript:V(1946)) [**L**](javascript:L(1947)) [**D**](javascript:D(1948)) [**V**](javascript:V(1949)) [**V**](javascript:V(1950)) [**T**](javascript:T(1951)) [**E**](javascript:E(1952)) [**A**](javascript:A(1953)) [**G**](javascript:G(1954)) [**R**](javascript:R(1955)) [**D**](javascript:D(1956)) [**W**](javascript:W(1957)) [**L**](javascript:L(1958)) [**L**](javascript:L(1959)) [**L**](javascript:L(1960))

**Primer KO-*neo*-F1**

**GGGCGAAGTGCCGGGGCAGGATCTCCTGTCATCTCACCTTGCTCCTGCCGAGAAAGTATC**

[**G**](javascript:G(1961)) [**E**](javascript:E(1962)) [**V**](javascript:V(1963)) [**P**](javascript:P(1964)) [**G**](javascript:G(1965)) [**Q**](javascript:Q(1966)) [**D**](javascript:D(1967)) [**L**](javascript:L(1968)) [**L**](javascript:L(1969)) [**S**](javascript:S(1970)) [**S**](javascript:S(1971)) [**H**](javascript:H(1972)) [**L**](javascript:L(1973)) [**A**](javascript:A(1974)) [**P**](javascript:P(1975)) [**A**](javascript:A(1976)) [**E**](javascript:E(1977)) [**K**](javascript:K(1978)) [**V**](javascript:V(1979)) [**S**](javascript:S(1980))

**CATCATGGCTGATGCAATGCGGCGGCTGCATACGCTTGATCCGGCTACCTGCCCATTCGA**

[**I**](javascript:I(1981)) [**M**](javascript:M(1982)) [**A**](javascript:A(1983)) [**D**](javascript:D(1984)) [**A**](javascript:A(1985)) [**M**](javascript:M(1986)) [**R**](javascript:R(1987)) [**R**](javascript:R(1988)) [**L**](javascript:L(1989)) [**H**](javascript:H(1990)) [**T**](javascript:T(1991)) [**L**](javascript:L(1992)) [**D**](javascript:D(1993)) [**P**](javascript:P(1994)) [**A**](javascript:A(1995)) [**T**](javascript:T(1996)) [**C**](javascript:C(1997)) [**P**](javascript:P(1998)) [**F**](javascript:F(1999)) [**D**](javascript:D(2000))

**Primer KO-*neo*-F2**

**CCACCAAGCGAAACATCGCATCGAGCGAGCACGTACTCGGATGGAAGCCGGTCTTGTCGA**

[**H**](javascript:H(2001)) [**Q**](javascript:Q(2002)) [**A**](javascript:A(2003)) [**K**](javascript:K(2004)) [**H**](javascript:H(2005)) [**R**](javascript:R(2006)) [**I**](javascript:I(2007)) [**E**](javascript:E(2008)) [**R**](javascript:R(2009)) [**A**](javascript:A(2010)) [**R**](javascript:R(2011)) [**T**](javascript:T(2012)) [**R**](javascript:R(2013)) [**M**](javascript:M(2014)) [**E**](javascript:E(2015)) [**A**](javascript:A(2016)) [**G**](javascript:G(2017)) [**L**](javascript:L(2018)) [**V**](javascript:V(2019)) [**D**](javascript:D(2020))

**TCAGGATGATCTGGACGAAGAGCATCAGGGGCTCGCGCCAGCCGAACTGTTCGCCAGGCT**

[**Q**](javascript:Q(2021)) [**D**](javascript:D(2022)) [**D**](javascript:D(2023)) [**L**](javascript:L(2024)) [**D**](javascript:D(2025)) [**E**](javascript:E(2026)) [**E**](javascript:E(2027)) [**H**](javascript:H(2028)) [**Q**](javascript:Q(2029)) [**G**](javascript:G(2030)) [**L**](javascript:L(2031)) [**A**](javascript:A(2032)) [**P**](javascript:P(2033)) [**A**](javascript:A(2034)) [**E**](javascript:E(2035)) [**L**](javascript:L(2036)) [**F**](javascript:F(2037)) [**A**](javascript:A(2038)) [**R**](javascript:R(2039)) [**L**](javascript:L(2040))

**CAAGGCGCGCATGCCCGACGGCGAGGATCTCGTCGTGACCCATGGCGATGCCTGCTTGCC**

[**K**](javascript:K(2041)) [**A**](javascript:A(2042)) [**R**](javascript:R(2043)) [**M**](javascript:M(2044)) [**P**](javascript:P(2045)) [**D**](javascript:D(2046)) [**G**](javascript:G(2047)) [**E**](javascript:E(2048)) [**D**](javascript:D(2049)) [**L**](javascript:L(2050)) [**V**](javascript:V(2051)) [**V**](javascript:V(2052)) [**T**](javascript:T(2053)) [**H**](javascript:H(2054)) [**G**](javascript:G(2055)) [**D**](javascript:D(2056)) [**A**](javascript:A(2057)) [**C**](javascript:C(2058)) [**L**](javascript:L(2059)) [**P**](javascript:P(2060))

**GAATATCATGGTGGAAAATGGCCGCTTTTCTGGATTCATCGACTGTGGCCGGCTGGGTGT**

[**N**](javascript:N(2061)) [**I**](javascript:I(2062)) [**M**](javascript:M(2063)) [**V**](javascript:V(2064)) [**E**](javascript:E(2065)) [**N**](javascript:N(2066)) [**G**](javascript:G(2067)) [**R**](javascript:R(2068)) [**F**](javascript:F(2069)) [**S**](javascript:S(2070)) [**G**](javascript:G(2071)) [**F**](javascript:F(2072)) [**I**](javascript:I(2073)) [**D**](javascript:D(2074)) [**C**](javascript:C(2075)) [**G**](javascript:G(2076)) [**R**](javascript:R(2077)) [**L**](javascript:L(2078)) [**G**](javascript:G(2079)) [**V**](javascript:V(2080))

**Primer KO-*neo*-R2**

**GGCGGACCGCTATCAGGACATAGCGTTGGCTACCCGTGATATTGCTGAAGAGCTTGGCGG**

[**A**](javascript:A(2081)) [**D**](javascript:D(2082)) [**R**](javascript:R(2083)) [**Y**](javascript:Y(2084)) [**Q**](javascript:Q(2085)) [**D**](javascript:D(2086)) [**I**](javascript:I(2087)) [**A**](javascript:A(2088)) [**L**](javascript:L(2089)) [**A**](javascript:A(2090)) [**T**](javascript:T(2091)) [**R**](javascript:R(2092)) [**D**](javascript:D(2093)) [**I**](javascript:I(2094)) [**A**](javascript:A(2095)) [**E**](javascript:E(2096)) [**E**](javascript:E(2097)) [**L**](javascript:L(2098)) [**G**](javascript:G(2099)) [**G**](javascript:G(2100))

**CGAATGGGCTGACCGCTTCCTCGTGCTTTACGGTATCGCCGCTCCCGATTCGCAGCGCAT**

[**E**](javascript:E(2101)) [**W**](javascript:W(2102)) [**A**](javascript:A(2103)) [**D**](javascript:D(2104)) [**R**](javascript:R(2105)) [**F**](javascript:F(2106)) [**L**](javascript:L(2107)) [**V**](javascript:V(2108)) [**L**](javascript:L(2109)) [**Y**](javascript:Y(2110)) [**G**](javascript:G(2111)) [**I**](javascript:I(2112)) [**A**](javascript:A(2113)) [**A**](javascript:A(2114)) [**P**](javascript:P(2115)) [**D**](javascript:D(2116)) [**S**](javascript:S(2117)) [**Q**](javascript:Q(2118)) [**R**](javascript:R(2119)) [**I**](javascript:I(2120))

**End of βGEO ORF**

**Primer KO-*neo*-R1**

**CGCCTTCTATCGCCTTCTTGACGAGTTCTTCTGAGCGGGACTCTGGGGTTCGAAATGACC**

[**A**](javascript:A(2121)) [**F**](javascript:F(2122)) [**Y**](javascript:Y(2123)) [**R**](javascript:R(2124)) [**L**](javascript:L(2125)) [**L**](javascript:L(2126)) [**D**](javascript:D(2127)) [**E**](javascript:E(2128)) [**F**](javascript:F(2129)) [**F**](javascript:F(2130)) [*****](javascript:Z(2131))

**GACCAAGCGACGCCCAACCTGCCATCACGAGATTTCGATTCCACCGCCGCCTTCTATGAA**

**AGGTTGGGCTTCGGAATCGTTTTCCGGGACGCCGGCTGGATGATCCTCCAGCGCGGGGAT**

**CTCATGCTGGAGTTCTTCGCCCACCCCCCGGATCTAAGCTCTAGCCGGCCGCCTCGAGAA**

**GTTCCTATTCCGAAGTTCCTATTCTCTAGAAAGTATAGGAACTTCTAAGGACGAGCTGTG**

**ATTGAATTCCGCCCCCCCCCCCCCCCCCTCTCCCTCCCCCCCCCCTAACGTTACTGGCCG**

**AAGCCGCTTGGAATAAGGCCGGTGTGCGTTTGTCTATATGTTATTTTCCACCATATTGCC**

**GTCTTTTGGCAATGTGAGGGCCCGGAAACCTGGCCCTGTCTTCTTGACGAGCATTCCTAG**

**GGGTCTTTCCCCTCTCGCCAAAGGAATGCAAGGTCTGTTGAATGTCGTGAAGGAAGCAGT**

**TCCTCTGGAAGCTTCTTGAAGACAAACAACGTCTGTAGCGACCCTTTGCAGGCAGCGGAA**

**CCCCCCACCTGGCGACAGGTGCCTCTGCGGCCAAAAGCCACGTGTATAAGATACACCTGC**

**AAAGGCGGCACAACCCCAGTGCCACGTTGTGAGTTGGATAGTTGTGGAAAGAGTCAAATG**

**GCTCTCCTCAAGCGTATTCAACAAGGGGCTGAAGGATGCCCAGAAGGTACCCCATTGTAT**

**GGGATCTGATCTGGGGCCTCGGTGCACATGCTTTACGTGTGTTTAGTCGAGGTTAAAAAA**

**PLAP ORF**

**CGTCTAGGCCCCCCGAACCACGGGGACGTGGTTTTCCTTTGAAAAACACGATGATAATAT**

[**M**](javascript:M(1))

**GGCCACAACCATGCTGCTGCTGCTGCTGCTGCTGGGCCTGAGGCTACAGCTCTCCCTGGG**

[**A**](javascript:A(2)) [**T**](javascript:T(3)) [**T**](javascript:T(4)) [**M**](javascript:M(5)) [**L**](javascript:L(6)) [**L**](javascript:L(7)) [**L**](javascript:L(8)) [**L**](javascript:L(9)) [**L**](javascript:L(10)) [**L**](javascript:L(11)) [**L**](javascript:L(12)) [**G**](javascript:G(13)) [**L**](javascript:L(14)) [**R**](javascript:R(15)) [**L**](javascript:L(16)) [**Q**](javascript:Q(17)) [**L**](javascript:L(18)) [**S**](javascript:S(19)) [**L**](javascript:L(20)) [**G**](javascript:G(21))

**CATCATCCCAGTTGAGGAGGAGAACCCGGACTTCTGGAACCGCGAGGCAGCCGAGGCCCT**

[**I**](javascript:I(22)) [**I**](javascript:I(23)) [**P**](javascript:P(24)) [**V**](javascript:V(25)) [**E**](javascript:E(26)) [**E**](javascript:E(27)) [**E**](javascript:E(28)) [**N**](javascript:N(29)) [**P**](javascript:P(30)) [**D**](javascript:D(31)) [**F**](javascript:F(32)) [**W**](javascript:W(33)) [**N**](javascript:N(34)) [**R**](javascript:R(35)) [**E**](javascript:E(36)) [**A**](javascript:A(37)) [**A**](javascript:A(38)) [**E**](javascript:E(39)) [**A**](javascript:A(40)) [**L**](javascript:L(41))

**GGGTGCCGCCAAGAAGCTGCAGCCTGCACAGACAGCCGCCAAGAACCTCATCATCTTCCT**

[**G**](javascript:G(42)) [**A**](javascript:A(43)) [**A**](javascript:A(44)) [**K**](javascript:K(45)) [**K**](javascript:K(46)) [**L**](javascript:L(47)) [**Q**](javascript:Q(48)) [**P**](javascript:P(49)) [**A**](javascript:A(50)) [**Q**](javascript:Q(51)) [**T**](javascript:T(52)) [**A**](javascript:A(53)) [**A**](javascript:A(54)) [**K**](javascript:K(55)) [**N**](javascript:N(56)) [**L**](javascript:L(57)) [**I**](javascript:I(58)) [**I**](javascript:I(59)) [**F**](javascript:F(60)) [**L**](javascript:L(61))

**GGGCGATGGGATGGGGGTGTCTACGGTGACAGCTGCCAGGATCCTAAAAGGGCAGAAGAA**

[**G**](javascript:G(62)) [**D**](javascript:D(63)) [**G**](javascript:G(64)) [**M**](javascript:M(65)) [**G**](javascript:G(66)) [**V**](javascript:V(67)) [**S**](javascript:S(68)) [**T**](javascript:T(69)) [**V**](javascript:V(70)) [**T**](javascript:T(71)) [**A**](javascript:A(72)) [**A**](javascript:A(73)) [**R**](javascript:R(74)) [**I**](javascript:I(75)) [**L**](javascript:L(76)) [**K**](javascript:K(77)) [**G**](javascript:G(78)) [**Q**](javascript:Q(79)) [**K**](javascript:K(80)) [**K**](javascript:K(81))

**GGACAAACTGGGGCCTGAGATACCCCTGGCCATGGACCGCTTCCCATATGTGGCTCTGTC**

[**D**](javascript:D(82)) [**K**](javascript:K(83)) [**L**](javascript:L(84)) [**G**](javascript:G(85)) [**P**](javascript:P(86)) [**E**](javascript:E(87)) [**I**](javascript:I(88)) [**P**](javascript:P(89)) [**L**](javascript:L(90)) [**A**](javascript:A(91)) [**M**](javascript:M(92)) [**D**](javascript:D(93)) [**R**](javascript:R(94)) [**F**](javascript:F(95)) [**P**](javascript:P(96)) [**Y**](javascript:Y(97)) [**V**](javascript:V(98)) [**A**](javascript:A(99)) [**L**](javascript:L(100)) [**S**](javascript:S(101))

**CAAGACATACAATGTAGACAAACATGTGCCAGACAGTGGAGCCACAGCCACGGCCTACCT**

[**K**](javascript:K(102)) [**T**](javascript:T(103)) [**Y**](javascript:Y(104)) [**N**](javascript:N(105)) [**V**](javascript:V(106)) [**D**](javascript:D(107)) [**K**](javascript:K(108)) [**H**](javascript:H(109)) [**V**](javascript:V(110)) [**P**](javascript:P(111)) [**D**](javascript:D(112)) [**S**](javascript:S(113)) [**G**](javascript:G(114)) [**A**](javascript:A(115)) [**T**](javascript:T(116)) [**A**](javascript:A(117)) [**T**](javascript:T(118)) [**A**](javascript:A(119)) [**Y**](javascript:Y(120)) [**L**](javascript:L(121))

**GTGCGGGGTCAAGGGCAACTTCCAGACCATTGGCTTGAGTGCAGCCGCCCGCTTTAACCA**

[**C**](javascript:C(122)) [**G**](javascript:G(123)) [**V**](javascript:V(124)) [**K**](javascript:K(125)) [**G**](javascript:G(126)) [**N**](javascript:N(127)) [**F**](javascript:F(128)) [**Q**](javascript:Q(129)) [**T**](javascript:T(130)) [**I**](javascript:I(131)) [**G**](javascript:G(132)) [**L**](javascript:L(133)) [**S**](javascript:S(134)) [**A**](javascript:A(135)) [**A**](javascript:A(136)) [**A**](javascript:A(137)) [**R**](javascript:R(138)) [**F**](javascript:F(139)) [**N**](javascript:N(140)) [**Q**](javascript:Q(141))

**GTGCAACACGACACGCGGCAACGAGGTCATCTCCGTGATGAATCGGGCCAAGAAAGCAGG**

[**C**](javascript:C(142)) [**N**](javascript:N(143)) [**T**](javascript:T(144)) [**T**](javascript:T(145)) [**R**](javascript:R(146)) [**G**](javascript:G(147)) [**N**](javascript:N(148)) [**E**](javascript:E(149)) [**V**](javascript:V(150)) [**I**](javascript:I(151)) [**S**](javascript:S(152)) [**V**](javascript:V(153)) [**M**](javascript:M(154)) [**N**](javascript:N(155)) [**R**](javascript:R(156)) [**A**](javascript:A(157)) [**K**](javascript:K(158)) [**K**](javascript:K(159)) [**A**](javascript:A(160)) [**G**](javascript:G(161))

**GAAGTCAGTGGGAGTGGTAACCACCACACGAGTGCAGCACGCCTCGCCAGCCGGCACCTA**

[**K**](javascript:K(162)) [**S**](javascript:S(163)) [**V**](javascript:V(164)) [**G**](javascript:G(165)) [**V**](javascript:V(166)) [**V**](javascript:V(167)) [**T**](javascript:T(168)) [**T**](javascript:T(169)) [**T**](javascript:T(170)) [**R**](javascript:R(171)) [**V**](javascript:V(172)) [**Q**](javascript:Q(173)) [**H**](javascript:H(174)) [**A**](javascript:A(175)) [**S**](javascript:S(176)) [**P**](javascript:P(177)) [**A**](javascript:A(178)) [**G**](javascript:G(179)) [**T**](javascript:T(180)) [**Y**](javascript:Y(181))

**CGCCCACACGGTGAACCGCAACTGGTACTCGGACGCCGACGTGCCTGCCTCGGCCCGCCA**

[**A**](javascript:A(182)) [**H**](javascript:H(183)) [**T**](javascript:T(184)) [**V**](javascript:V(185)) [**N**](javascript:N(186)) [**R**](javascript:R(187)) [**N**](javascript:N(188)) [**W**](javascript:W(189)) [**Y**](javascript:Y(190)) [**S**](javascript:S(191)) [**D**](javascript:D(192)) [**A**](javascript:A(193)) [**D**](javascript:D(194)) [**V**](javascript:V(195)) [**P**](javascript:P(196)) [**A**](javascript:A(197)) [**S**](javascript:S(198)) [**A**](javascript:A(199)) [**R**](javascript:R(200)) [**Q**](javascript:Q(201))

**GGAGGGGTGCCAGGACATCGCTACGCAGCTCATCTCCAACATGGACATTGATGTGATCCT**

[**E**](javascript:E(202)) [**G**](javascript:G(203)) [**C**](javascript:C(204)) [**Q**](javascript:Q(205)) [**D**](javascript:D(206)) [**I**](javascript:I(207)) [**A**](javascript:A(208)) [**T**](javascript:T(209)) [**Q**](javascript:Q(210)) [**L**](javascript:L(211)) [**I**](javascript:I(212)) [**S**](javascript:S(213)) [**N**](javascript:N(214)) [**M**](javascript:M(215)) [**D**](javascript:D(216)) [**I**](javascript:I(217)) [**D**](javascript:D(218)) [**V**](javascript:V(219)) [**I**](javascript:I(220)) [**L**](javascript:L(221))

**AGGTGGAGGCCGAAAGTACATGTTTCGCATGGGAACCCCAGACCCTGAGTACCCAGATGA**

[**G**](javascript:G(222)) [**G**](javascript:G(223)) [**G**](javascript:G(224)) [**R**](javascript:R(225)) [**K**](javascript:K(226)) [**Y**](javascript:Y(227)) [**M**](javascript:M(228)) [**F**](javascript:F(229)) [**R**](javascript:R(230)) [**M**](javascript:M(231)) [**G**](javascript:G(232)) [**T**](javascript:T(233)) [**P**](javascript:P(234)) [**D**](javascript:D(235)) [**P**](javascript:P(236)) [**E**](javascript:E(237)) [**Y**](javascript:Y(238)) [**P**](javascript:P(239)) [**D**](javascript:D(240)) [**D**](javascript:D(241))

**CTACAGCCAAGGTGGGACCAGGCTGGACGGGAAGAATCTGGTGCAGGAATGGCTCGGCGA**

[**Y**](javascript:Y(242)) [**S**](javascript:S(243)) [**Q**](javascript:Q(244)) [**G**](javascript:G(245)) [**G**](javascript:G(246)) [**T**](javascript:T(247)) [**R**](javascript:R(248)) [**L**](javascript:L(249)) [**D**](javascript:D(250)) [**G**](javascript:G(251)) [**K**](javascript:K(252)) [**N**](javascript:N(253)) [**L**](javascript:L(254)) [**V**](javascript:V(255)) [**Q**](javascript:Q(256)) [**E**](javascript:E(257)) [**W**](javascript:W(258)) [**L**](javascript:L(259)) [**G**](javascript:G(260)) [**E**](javascript:E(261))

**ACGCCAGGGTGCCCGGTACGTGTGGAACCGCACTGAGCTCATGCAGGCTTCCCTGGACCC**

[**R**](javascript:R(262)) [**Q**](javascript:Q(263)) [**G**](javascript:G(264)) [**A**](javascript:A(265)) [**R**](javascript:R(266)) [**Y**](javascript:Y(267)) [**V**](javascript:V(268)) [**W**](javascript:W(269)) [**N**](javascript:N(270)) [**R**](javascript:R(271)) [**T**](javascript:T(272)) [**E**](javascript:E(273)) [**L**](javascript:L(274)) [**M**](javascript:M(275)) [**Q**](javascript:Q(276)) [**A**](javascript:A(277)) [**S**](javascript:S(278)) [**L**](javascript:L(279)) [**D**](javascript:D(280)) [**P**](javascript:P(281))

**GTCTGTGACCCATCTCATGGGTCTCTTTGAGCCTGGAGACATGAAATACGAGATCCACCG**

[**S**](javascript:S(282)) [**V**](javascript:V(283)) [**T**](javascript:T(284)) [**H**](javascript:H(285)) [**L**](javascript:L(286)) [**M**](javascript:M(287)) [**G**](javascript:G(288)) [**L**](javascript:L(289)) [**F**](javascript:F(290)) [**E**](javascript:E(291)) [**P**](javascript:P(292)) [**G**](javascript:G(293)) [**D**](javascript:D(294)) [**M**](javascript:M(295)) [**K**](javascript:K(296)) [**Y**](javascript:Y(297)) [**E**](javascript:E(298)) [**I**](javascript:I(299)) [**H**](javascript:H(300)) [**R**](javascript:R(301))

**AGACTCCACACTGGACCCCTCCCTGATGGAGATGACAGAGGCTGCCCTGCGCCTGCTGAG**

[**D**](javascript:D(302)) [**S**](javascript:S(303)) [**T**](javascript:T(304)) [**L**](javascript:L(305)) [**D**](javascript:D(306)) [**P**](javascript:P(307)) [**S**](javascript:S(308)) [**L**](javascript:L(309)) [**M**](javascript:M(310)) [**E**](javascript:E(311)) [**M**](javascript:M(312)) [**T**](javascript:T(313)) [**E**](javascript:E(314)) [**A**](javascript:A(315)) [**A**](javascript:A(316)) [**L**](javascript:L(317)) [**R**](javascript:R(318)) [**L**](javascript:L(319)) [**L**](javascript:L(320)) [**S**](javascript:S(321))

**CAGACACCCCCGCGGCTTCTTCCTCTTCGTGGAGGGTGGTCGCATCGACCATGGTCATCA**

[**R**](javascript:R(322)) [**H**](javascript:H(323)) [**P**](javascript:P(324)) [**R**](javascript:R(325)) [**G**](javascript:G(326)) [**F**](javascript:F(327)) [**F**](javascript:F(328)) [**L**](javascript:L(329)) [**F**](javascript:F(330)) [**V**](javascript:V(331)) [**E**](javascript:E(332)) [**G**](javascript:G(333)) [**G**](javascript:G(334)) [**R**](javascript:R(335)) [**I**](javascript:I(336)) [**D**](javascript:D(337)) [**H**](javascript:H(338)) [**G**](javascript:G(339)) [**H**](javascript:H(340)) [**H**](javascript:H(341))

**TGAAAGCAGGGCTTACCGGGCACTGACTGAGACGATCATGTTCGACGACGCCATTGAGAG**

[**E**](javascript:E(342)) [**S**](javascript:S(343)) [**R**](javascript:R(344)) [**A**](javascript:A(345)) [**Y**](javascript:Y(346)) [**R**](javascript:R(347)) [**A**](javascript:A(348)) [**L**](javascript:L(349)) [**T**](javascript:T(350)) [**E**](javascript:E(351)) [**T**](javascript:T(352)) [**I**](javascript:I(353)) [**M**](javascript:M(354)) [**F**](javascript:F(355)) [**D**](javascript:D(356)) [**D**](javascript:D(357)) [**A**](javascript:A(358)) [**I**](javascript:I(359)) [**E**](javascript:E(360)) [**R**](javascript:R(361))

**GGCGGGCCAGCTCACCAGCGAGGAGGACACGCTGAGCCTCGTCACTGCCGACCACTCCCA**

[**A**](javascript:A(362)) [**G**](javascript:G(363)) [**Q**](javascript:Q(364)) [**L**](javascript:L(365)) [**T**](javascript:T(366)) [**S**](javascript:S(367)) [**E**](javascript:E(368)) [**E**](javascript:E(369)) [**D**](javascript:D(370)) [**T**](javascript:T(371)) [**L**](javascript:L(372)) [**S**](javascript:S(373)) [**L**](javascript:L(374)) [**V**](javascript:V(375)) [**T**](javascript:T(376)) [**A**](javascript:A(377)) [**D**](javascript:D(378)) [**H**](javascript:H(379)) [**S**](javascript:S(380)) [**H**](javascript:H(381))

**CGTCTTCTCCTTCGGAGGCTACCCCCTGCGAGGGAGCTCCTTCATCGGGCTGGCCGCTGG**

[**V**](javascript:V(382)) [**F**](javascript:F(383)) [**S**](javascript:S(384)) [**F**](javascript:F(385)) [**G**](javascript:G(386)) [**G**](javascript:G(387)) [**Y**](javascript:Y(388)) [**P**](javascript:P(389)) [**L**](javascript:L(390)) [**R**](javascript:R(391)) [**G**](javascript:G(392)) [**S**](javascript:S(393)) [**S**](javascript:S(394)) [**F**](javascript:F(395)) [**I**](javascript:I(396)) [**G**](javascript:G(397)) [**L**](javascript:L(398)) [**A**](javascript:A(399)) [**A**](javascript:A(400)) [**G**](javascript:G(401))

**CAAGGCCCGGGACAGGAAGGCCTACACGGTCCTCCTATACGGAAACGGTCCAGGCTATGT**

[**K**](javascript:K(402)) [**A**](javascript:A(403)) [**R**](javascript:R(404)) [**D**](javascript:D(405)) [**R**](javascript:R(406)) [**K**](javascript:K(407)) [**A**](javascript:A(408)) [**Y**](javascript:Y(409)) [**T**](javascript:T(410)) [**V**](javascript:V(411)) [**L**](javascript:L(412)) [**L**](javascript:L(413)) [**Y**](javascript:Y(414)) [**G**](javascript:G(415)) [**N**](javascript:N(416)) [**G**](javascript:G(417)) [**P**](javascript:P(418)) [**G**](javascript:G(419)) [**Y**](javascript:Y(420)) [**V**](javascript:V(421))

**GCTCAAGGACGGCGCCCGGCCGGATGTTACCGAGAGCGAGAGCGGGAGCCCCGAGTATCG**

[**L**](javascript:L(422)) [**K**](javascript:K(423)) [**D**](javascript:D(424)) [**G**](javascript:G(425)) [**A**](javascript:A(426)) [**R**](javascript:R(427)) [**P**](javascript:P(428)) [**D**](javascript:D(429)) [**V**](javascript:V(430)) [**T**](javascript:T(431)) [**E**](javascript:E(432)) [**S**](javascript:S(433)) [**E**](javascript:E(434)) [**S**](javascript:S(435)) [**G**](javascript:G(436)) [**S**](javascript:S(437)) [**P**](javascript:P(438)) [**E**](javascript:E(439)) [**Y**](javascript:Y(440)) [**R**](javascript:R(441))

**GCAGCAGTCAGCAGTGCCCCTGGACGAAGAGACCCACGCAGGCGAGGACGTGGCGGTGTT**

[**Q**](javascript:Q(442)) [**Q**](javascript:Q(443)) [**S**](javascript:S(444)) [**A**](javascript:A(445)) [**V**](javascript:V(446)) [**P**](javascript:P(447)) [**L**](javascript:L(448)) [**D**](javascript:D(449)) [**E**](javascript:E(450)) [**E**](javascript:E(451)) [**T**](javascript:T(452)) [**H**](javascript:H(453)) [**A**](javascript:A(454)) [**G**](javascript:G(455)) [**E**](javascript:E(456)) [**D**](javascript:D(457)) [**V**](javascript:V(458)) [**A**](javascript:A(459)) [**V**](javascript:V(460)) [**F**](javascript:F(461))

**CGCGCGCGGCCCGCAGGCGCACCTGGTTCACGGCGTGCAGGAGCAGACCTTCATAGCGCA**

[**A**](javascript:A(462)) [**R**](javascript:R(463)) [**G**](javascript:G(464)) [**P**](javascript:P(465)) [**Q**](javascript:Q(466)) [**A**](javascript:A(467)) [**H**](javascript:H(468)) [**L**](javascript:L(469)) [**V**](javascript:V(470)) [**H**](javascript:H(471)) [**G**](javascript:G(472)) [**V**](javascript:V(473)) [**Q**](javascript:Q(474)) [**E**](javascript:E(475)) [**Q**](javascript:Q(476)) [**T**](javascript:T(477)) [**F**](javascript:F(478)) [**I**](javascript:I(479)) [**A**](javascript:A(480)) [**H**](javascript:H(481))

**CGTCATGGCCTTCGCCGCCTGCCTGGAGCCCTACACCGCCTGCGACCTGGCGCCCCCCGC**

[**V**](javascript:V(482)) [**M**](javascript:M(483)) [**A**](javascript:A(484)) [**F**](javascript:F(485)) [**A**](javascript:A(486)) [**A**](javascript:A(487)) [**C**](javascript:C(488)) [**L**](javascript:L(489)) [**E**](javascript:E(490)) [**P**](javascript:P(491)) [**Y**](javascript:Y(492)) [**T**](javascript:T(493)) [**A**](javascript:A(494)) [**C**](javascript:C(495)) [**D**](javascript:D(496)) [**L**](javascript:L(497)) [**A**](javascript:A(498)) [**P**](javascript:P(499)) [**P**](javascript:P(500)) [**A**](javascript:A(501))

**CGGCACCACCGACGCCGCGCACCCGGGGCGGTCCGTGGTCCCCGCGTTGCTTCCTCTGCT**

[**G**](javascript:G(502)) [**T**](javascript:T(503)) [**T**](javascript:T(504)) [**D**](javascript:D(505)) [**A**](javascript:A(506)) [**A**](javascript:A(507)) [**H**](javascript:H(508)) [**P**](javascript:P(509)) [**G**](javascript:G(510)) [**R**](javascript:R(511)) [**S**](javascript:S(512)) [**V**](javascript:V(513)) [**V**](javascript:V(514)) [**P**](javascript:P(515)) [**A**](javascript:A(516)) [**L**](javascript:L(517)) [**L**](javascript:L(518)) [**P**](javascript:P(519)) [**L**](javascript:L(520)) [**L**](javascript:L(521))

**End of PLAP ORF**

**GGCCGGGACCCTGCTGCTGCTGGAGACGGCCACTGCTCCCTGAGTGTCCCGTCCCTGGGG**

[**A**](javascript:A(522)) [**G**](javascript:G(523)) [**T**](javascript:T(524)) [**L**](javascript:L(525)) [**L**](javascript:L(526)) [**L**](javascript:L(527)) [**L**](javascript:L(528)) [**E**](javascript:E(529)) [**T**](javascript:T(530)) [**A**](javascript:A(531)) [**T**](javascript:T(532)) [**A**](javascript:A(533)) [**P**](javascript:P(534)) [*****](javascript:Z(535))

**CTCCTGCTTCCCCATCCCGGAGTTCTCCTGCTCCCCACCTCCTGTCGTCCTGCCTGGCCT**

**CCAGCCCGAGTCGTCATCCCCGGAGTCCCTATACAGAGGTCCTGCCATGGAACCTTCCCC**

**TCCCCGTGCGCTCTGGGGACTGAGCCCATGACACCAAACCTGCCCCTTGGCTGCTCTCGG**

**ACTCCCTACCCCAACCCCAGGGACTGCAGGTTGTGCCCTGTGGCTGCCTGCACCCCAGGA**

**AAGGAGGGGGCTCAGGCCATCCAGCCACCACCTACAGCCCAGTGGGTCGCTCTAGAGCGA**

**CCTCGAGGGGCTAGANNTGATCATAATCAGCCATACCACATTTGTAGAGGTTTTACTTGC**

**TTTAAAAAACCTCCCACACCTCCCCCTGAACCTGAAACATAAAATGAATGCAATTGTTGT**

**TGTTAACTTGTTTATTGCAGCTTATAATGGTTACAAATAAAGCAATAGCATCACAAATTT**

**CACAAATAAAGCATTTTTTTCACTGCATTCTAGTTGTGGTTTGTCCAAACTCATCAATGT**

**ATCTTATCATGTCTGGATCCCCGGGCGAGCTCGAATTCGTAATCATGGTCATAGCTGTTT**

**CCTGTGTGAAATTGTTATCCGCTCACAATTCCACACAACATACGAGCCGGAAGCATAAAG**

**TGTAAAGCCTGGGGTGCCTAATGAGTGAGCTAACTCACATTAATTGCGTTGCGCTCACTG**

**CCCGCTTTCCAGTCGGGAAACCTGTCGTGCCAGCTGCATTAATGAATCGGCCAACGCGCG**

**GGGAGAGGCGGTTTGCGTATTGGGCGCTCTTCCGCTTCCTCGCTCACTGACTCGCTGCGC**

**TCGGTCGTTCGGCTGCGGCGAGCGGTATCAGCTCACTCAAAGGCGGTAATACGGTTATCC**

**ACAGAATCAGGGGATAACGCAGGAAAGAACATGTGAGCAAAAGGCCAGCAAAAGGCCAGG**

**AACCGTAAAAAGGCCGCGTTGCTGGCGTTTTTCCATAGGCTCCGCCCCCCTGACGAGCAT**

**CACAAAAATCGACGCTCAAGTCAGAGGTGGCGAAACCCGACAGGACTATAAAGATACCAG**

**GCGTTTCCCCCTGGAAGCTCCCTCGTGCGCTCTCCTGTTCCGACCCTGCCGCTTACCGGA**

**TACCTGTCCGCCTTTCTCCCTTCGGGAAGCGTGGCGCTTTCTCATAGCTCACGCTGTAGG**

**TATCTCAGTTCGGTGTAGGTCGTTCGCTCCAAGCTGGGCTGTGTGCACGAACCCCCCGTT**

**CAGCCCGACCGCTGCGCCTTATCCGGTAACTATCGTCTTGAGTCCAACCCGGTAAGACAC**

**GACTTATCGCCACTGGCAGCAGCCACTGGTAACAGGATTAGCAGAGCGAGGTATGTAGGC**

**GGTGCTACAGAGTTCTTGAAGTGGTGGCCTAACTACGGCTACACTAGAAGGACAGTATTT**

**GGTATCTGCGCTCTGCTGAAGCCAGTTACCTTCGGAAAAAGAGTTGGTAGCTCTTGATCC**

**GGCAAACAAACCACCGCTGGTAGCGGTGGTTTTTTTGTTTGCAAGCAGCAGATTACGCGC**

**AGAAAAAAAGGATCTCAAGAAGATCCTTTGATCTTTTCTACGGGGTCTGACGCTCAGTGG**

**AACGAAAACTCACGTTAAGGGATTTTGGTCATGAGATTATCAAAAAGGATCTTCACCTAG**

**ATCCTTTTAAATTAAAAATGAAGTTTTAAATCAATCTAAAGTATATATGAGTAAACTTGG**

**TCTGACAGTTACCAATGCTTAATCAGTGAGGCACCTATCTCAGCGATCTGTCTATTTCGT**

**TCATCCATAGTTGCCTGACTCCCCGTCGTGTAGATAACTACGATACGGGAGGGCTTACCA**

**TCTGGCCCCAGTGCTGCAATGATACCGCGAGACCCACGCTCACCGGCTCCAGATTTATCA**

**GCAATAAACCAGCCAGCCGGAAGGGCCGAGCGCAGAAGTGGTCCTGCAACTTTATCCGCC**

**TCCATCCAGTCTATTAATTGTTGCCGGGAAGCTAGAGTAAGTAGTTCGCCAGTTAATAGT**

**TTGCGCAACGTTGTTGCCATTGCTACAGGCATCGTGGTGTCACGCTCGTCGTTTGGTATG**

**GCTTCATTCAGCTCCGGTTCCCAACGATCAAGGCGAGTTACATGATCCCCCATGTTGTGC**

**AAAAAAGCGGTTAGCTCCTTCGGTCCTCCGATCGTTGTCAGAAGTAAGTTGGCCGCAGTG**

**TTATCACTCATGGTTATGGCAGCACTGCATAATTCTCTTACTGTCATGCCATCCGTAAGA**

**TGCTTTTCTGTGACTGGTGAGTACTCAACCAAGTCATTCTGAGAATAGTGTATGCGGCGA**

**CCGAGTTGCTCTTGCCCGGCGTCAATACGGGATAATACCGCGCCACATAGCAGAACTTTA**

**AAAGTGCTCATCATTGGAAAACGTTCTTCGGGGCGAAAACTCTCAAGGATCTTACCGCTG**

**TTGAGATCCAGTTCGATGTAACCCACTCGTGCACCCAACTGATCTTCAGCATCTTTTACT**

**TTCACCAGCGTTTCTGGGTGAGCAAAAACAGGAAGGCAAAATGCCGCAAAAAAGGGAATA**

**AGGGCGACACGGAAATGTTGAATACTCATACTCTTCCTTTTTCAATATTATTGAAGCATT**

**TATCAGGGTTATTGTCTCATGAGCGGATACATATTTGAATGTATTTAGAAAAATAAACAA**

**ATAGGGGTTCCGCGCACATTTCCCCGAAAAGTGCCACCTGACGTCTAAGAAACCATTATT**

**ATCATGACATTAACCTATAAAAATAGGCGTATCACGAGGCCCTTTCGTCTCGCGCGTTTC**

**GGTGATGACGGTGAAAACCTCTGACACATGCAGCTCCCGGAGACGGTCACAGCTTGTCTG**

**TAAGCGGATGCCGGGAGCAGACAAGCCCGTCAGGGCGCGTCAGCGGGTGTTGGCGGGTGT**

**CGGGGCTGGCTTAACTATGCGGCATCAGAGCAGATTGTACTGAGAGTGCACCATATGCGG**

**TGTGAAATACCGCACAGATGCGTAAGGAGAAAATACCGCATCAGGCGCCATTCGCCATTC**

**AGGCTGCGCAACTGTTGGGAAGGGCGATCGGTGCGGGCCTCTTCGCTATTACGCCAGCTG**

**GCGAAAGGGGGATGTGCTGCAAGGCGATTAAGTTGGGTAACGCCAGGGTTTTCCCAGTCA**

**CGACGTTGTAAAACGACGGCCAGTGCCAAGCTGCGTCGAC....................**

**mTrop1-EX4**

**.........................catcgcctttccagTGGCGTCTAAATGCTTGGCGA**

[**A**](javascript:A(63)) [**S**](javascript:S(64)) [**K**](javascript:K(65)) [**C**](javascript:C(66)) [**L**](javascript:L(67)) [**A**](javascript:A(68)) [**M**](javascript:M(69))

**TGAAAGCAGAAATGACTCACAGCAAGTCTGGGAGGAGGATAAAGCCCGAAGGGGCGATCC**

[**K**](javascript:K(70)) [**A**](javascript:A(71)) [**E**](javascript:E(72)) [**M**](javascript:M(73)) [**T**](javascript:T(74)) [**H**](javascript:H(75)) [**S**](javascript:S(76)) [**K**](javascript:K(77)) [**S**](javascript:S(78)) [**G**](javascript:G(79)) [**R**](javascript:R(80)) [**R**](javascript:R(81)) [**I**](javascript:I(82)) [**K**](javascript:K(83)) [**P**](javascript:P(84)) [**E**](javascript:E(85)) [**G**](javascript:G(86)) [**A**](javascript:A(87)) [**I**](javascript:I(88)) [**Q**](javascript:Q(89))

**AGAACAACGATGGGCTGTACGACCCCGACTGCGACGAGCAGGGGCTCTTCAAAGCCAAGC**

[**N**](javascript:N(90)) [**N**](javascript:N(91)) [**D**](javascript:D(92)) [**G**](javascript:G(93)) [**L**](javascript:L(94)) [**Y**](javascript:Y(95)) [**D**](javascript:D(96)) [**P**](javascript:P(97)) [**D**](javascript:D(98)) [**C**](javascript:C(99)) [**D**](javascript:D(100)) [**E**](javascript:E(101)) [**Q**](javascript:Q(102)) [**G**](javascript:G(103)) [**L**](javascript:L(104)) [**F**](javascript:F(105)) [**K**](javascript:K(106)) [**A**](javascript:A(107)) [**K**](javascript:K(108)) [**Q**](javascript:Q(109))

**AGTGCAACGGCACCGCCACGTGCTGGTGTGTCAACACCGCCGGAGTCCGAAGAACCGACA**

[**C**](javascript:C(110)) [**N**](javascript:N(111)) [**G**](javascript:G(112)) [**T**](javascript:T(113)) [**A**](javascript:A(114)) [**T**](javascript:T(115)) [**C**](javascript:C(116)) [**W**](javascript:W(117)) [**C**](javascript:C(118)) [**V**](javascript:V(119)) [**N**](javascript:N(120)) [**T**](javascript:T(121)) [**A**](javascript:A(122)) [**G**](javascript:G(123)) [**V**](javascript:V(124)) [**R**](javascript:R(125)) [**R**](javascript:R(126)) [**T**](javascript:T(127)) [**D**](javascript:D(128)) [**K**](javascript:K(129))

**mTrop1-int4 905 bp**

**AGGACACGGAGATCACGTGCTCCGAGCGCGTGAGGACCTAgtgagtgagctttccagtgc**

[**D**](javascript:D(130)) [**T**](javascript:T(131)) [**E**](javascript:E(132)) [**I**](javascript:I(133)) [**T**](javascript:T(134)) [**C**](javascript:C(135)) [**S**](javascript:S(136)) [**E**](javascript:E(137)) [**R**](javascript:R(138)) [**V**](javascript:V(139)) [**R**](javascript:R(140)) [**T**](javascript:T(141)) [**Y**](javascript:Y(142))

**gcttaagtttagctttgaataccaaataagatttttgtgcctcggaattcaaaaaaccgg**

**gaaaagccgggcgtagtagttcagggccagccacagtgagtttgagaccagcctgggcta**

**cttgagatcccttttggagtgacaagtgtccatcctgtctccgtgcctcatgaaatggca**

**Primer mT1INT3-R1**

**gtcaccagccttgagttgggctttattggccctaatccagttatatttgttttct.....**

**mTrop1-EX5**

**...............cagctgacaccatcttgtcttacagCTGGATCATCATTGAACTAA**

[**W**](javascript:W(143)) [**I**](javascript:I(144)) [**I**](javascript:I(145)) [**I**](javascript:I(146)) [**E**](javascript:E(147)) [**L**](javascript:L(148)) [**K**](javascript:K(149))

**mTrop1-int5 905 bp**

**AACACAAAGAAAGAGAAAGCCCCTACGACCATCAGAGCTTGCAGACgtgagtgtgattgc**

[**H**](javascript:H(150)) [**K**](javascript:K(151)) [**E**](javascript:E(152)) [**R**](javascript:R(153)) [**E**](javascript:E(154)) [**S**](javascript:S(155)) [**P**](javascript:P(156)) [**Y**](javascript:Y(157)) [**D**](javascript:D(158)) [**H**](javascript:H(159)) [**Q**](javascript:Q(160)) [**S**](javascript:S(161)) [**L**](javascript:L(162)) [**Q**](javascript:Q(163)) [**T**](javascript:T(164))

**mTrop1-EX6**

**acctatactac... ......aacgaaacttgtaaattatttgcagTGCGCTTCAAGAGG**

[**A**](javascript:A(165)) [**L**](javascript:L(166)) [**Q**](javascript:Q(167)) [**E**](javascript:E(168)) [**A**](javascript:A(169))

**mTrop1-int6 1376 bp**

**CGTTCACATCTCGATATAAGCTGAATCAGAAATTTATCAAAAACATTATGgtaagtttt**

[**F**](javascript:F(170)) [**T**](javascript:T(171)) [**S**](javascript:S(172)) [**R**](javascript:R(173)) [**Y**](javascript:Y(174)) [**K**](javascript:K(175)) [**L**](javascript:L(176)) [**N**](javascript:N(177)) [**Q**](javascript:Q(178)) [**K**](javascript:K(179)) [**F**](javascript:F(180)) [**I**](javascript:I(181)) [**K**](javascript:K(182)) [**N**](javascript:N(183)) [**I**](javascript:I(184)) [**M**](javascript:M(185))

**mTrop1-EX7**

**gtgggggggttctttt.........ttgtgttttcactttttctccccagTATGAGAATA**

[**Y**](javascript:Y(186)) [**E**](javascript:E(187)) [**N**](javascript:N(188)) [**N**](javascript:N(189))

**ATGTTATCACCATTGATCTGATGCAAAACTCTTCTCAGAAAACACAAGACGACGTGGACA**

[**V**](javascript:V(190)) [**I**](javascript:I(191)) [**T**](javascript:T(192)) [**I**](javascript:I(193)) [**D**](javascript:D(194)) [**L**](javascript:L(195)) [**M**](javascript:M(196)) [**Q**](javascript:Q(197)) [**N**](javascript:N(198)) [**S**](javascript:S(199)) [**S**](javascript:S(200)) [**Q**](javascript:Q(201)) [**K**](javascript:K(202)) [**T**](javascript:T(203)) [**Q**](javascript:Q(204)) [**D**](javascript:D(205)) [**D**](javascript:D(206)) [**V**](javascript:V(207)) [**D**](javascript:D(208)) [**I**](javascript:I(209))

**mTrop1-int7 2493 bp**

**TAGCTGATGTGGCTTACTATTTTGAAAAAGATgtaagtacaagccgtgtaattttta...**

[**A**](javascript:A(210)) [**D**](javascript:D(211)) [**V**](javascript:V(212)) [**A**](javascript:A(213)) [**Y**](javascript:Y(214)) [**Y**](javascript:Y(215)) [**F**](javascript:F(216)) [**E**](javascript:E(217)) [**K**](javascript:K(218)) [**D**](javascript:D(219))

**mTrop1-EX8**

**.......tctctactctccttctcctctgtagGTGAAGGGGGAGTCCCTGTTCCATTCTT**

[**V**](javascript:V(220)) [**K**](javascript:K(221)) [**G**](javascript:G(222)) [**E**](javascript:E(223)) [**S**](javascript:S(224)) [**L**](javascript:L(225)) [**F**](javascript:F(226)) [**H**](javascript:H(227)) [**S**](javascript:S(228)) [**S**](javascript:S(229))

**CTAAGAGCATGGACCTGAGAGTGAACGGAGAGCCGCTCGATCTGGACCCCGGGCAGACTC**

[**K**](javascript:K(230)) [**S**](javascript:S(231)) [**M**](javascript:M(232)) [**D**](javascript:D(233)) [**L**](javascript:L(234)) [**R**](javascript:R(235)) [**V**](javascript:V(236)) [**N**](javascript:N(237)) [**G**](javascript:G(238)) [**E**](javascript:E(239)) [**P**](javascript:P(240)) [**L**](javascript:L(241)) [**D**](javascript:D(242)) [**L**](javascript:L(243)) [**D**](javascript:D(244)) [**P**](javascript:P(245)) [**G**](javascript:G(246)) [**Q**](javascript:Q(247)) [**T**](javascript:T(248)) [**L**](javascript:L(249))

**TGATTTACTACGTTGATGAAAAGGCACCCGAGTTCTCCATGCAGGGCCTCACGGCCGGGA**

[**I**](javascript:I(250)) [**Y**](javascript:Y(251)) [**Y**](javascript:Y(252)) [**V**](javascript:V(253)) [**D**](javascript:D(254)) [**E**](javascript:E(255)) [**K**](javascript:K(256)) [**A**](javascript:A(257)) [**P**](javascript:P(258)) [**E**](javascript:E(259)) [**F**](javascript:F(260)) [**S**](javascript:S(261)) [**M**](javascript:M(262)) [**Q**](javascript:Q(263)) [**G**](javascript:G(264)) [**L**](javascript:L(265)) [**T**](javascript:T(266)) [**A**](javascript:A(267)) [**G**](javascript:G(268)) [**I**](javascript:I(269))

**mTrop1-int8**

**3273 bp**

**TCATCGCTGTCATTGTGGTGGTGTCATTAGCAGTCATCGCGGGGATTGTTGTCCTGgtg**

[**I**](javascript:I(270)) [**A**](javascript:A(271)) [**V**](javascript:V(272)) [**I**](javascript:I(273)) [**V**](javascript:V(274)) [**V**](javascript:V(275)) [**V**](javascript:V(276)) [**S**](javascript:S(277)) [**L**](javascript:L(278)) [**A**](javascript:A(279)) [**V**](javascript:V(280)) [**I**](javascript:I(281)) [**A**](javascript:A(282)) [**G**](javascript:G(283)) [**I**](javascript:I(284)) [**V**](javascript:V(285)) [**V**](javascript:V(286)) [**L**](javascript:L(287))

**mTrop1-EX9**

**agtacagggatgagtcagggct........ttaacagtaatggtttttctttcagGTTA**

[**V**](javascript:V(288)) [**I**](javascript:I(289))

**mTrop1-int9 769 bp**

**TATCTACAAGGAAGAAATCAGCAAAATATGAGAAGGCTGAGgtaagtggataaaagggta**

[**S**](javascript:S(290)) [**T**](javascript:T(291)) [**R**](javascript:R(292)) [**K**](javascript:K(293)) [**K**](javascript:K(294)) [**S**](javascript:S(295)) [**A**](javascript:A(296)) [**K**](javascript:K(297)) [**Y**](javascript:Y(298)) [**E**](javascript:E(299)) [**K**](javascript:K(300)) [**A**](javascript:A(301)) [**E**](javascript:E(302))

**mTrop1-EX10**

**tgctga..........aaccctgtttcctcctctgttgcagATAAAGGAGATGGGTGAGA**

[**I**](javascript:I(303)) [**K**](javascript:K(304)) [**E**](javascript:E(305)) [**M**](javascript:M(306)) [**G**](javascript:G(307)) [**E**](javascript:E(308)) [**I**](javascript:I(309))

**End of mTrop1 ORF**

**TCCACAGAGAGCTTAATGCCTAGCCGTGCTGAGTGCTGAACTGAGGAGGGGCCGCCCGAC**

[**H**](javascript:H(310)) [**R**](javascript:R(311)) [**E**](javascript:E(312)) [**L**](javascript:L(313)) [**N**](javascript:N(314)) [**A**](javascript:A(315)) [*****](javascript:Z(316))

**CGGAAGTGGCAGAAGAGCTCGGACTGCAGATGTATAAACCTGGGGAAGATGAAGACCTGC**

**GAAGGGTTACTGCTTTGATAGTTACTTTGTTAGTTTCACATTTGTAACAGTGAAATTTGT**

**ACTCGTAAATACAAGCAGCTGGACACCGGCATTACCGATCGTAAAATTAGACGAACGTCT**

**TATAGGTGCAGGTCCAGTGTGGTACTCAGAACTTAGCCTGCAAAGTTAAGAGAGTTGATG**

**CTTATTATGACAGAGTGTGCGTCGCAAACATTCCAACAGTAGAATGCGGTGACTAGTCTC**

**ATTTTTTTTTTTTTTTTGTGATTAAGGCTGCCCTTCTATATACCTGAGTCTTGTACATAA**

**TAAACTTTTTTTTAATGAAATAAAACATTTTAAAGTGAGTTTCTAAGTTTGTTTGAATCA**

**AATTTTCCTAGCATGTGCATAATTAAGATAATAGATGTCTAAATGCTCTGGCACTGCTAA**

**CTGGTACAAACCTGTAATTCTGTACTTGGGAGGTAGAGGTAGGAGGGTTAGCGCTTCCGA**

**GGTAGCTGCTGTGTATCTGCTCTGCCACTGACTGGCCTTGACTATCCAACACCCTATCTG**

**End of mTrop1 transcribed region**

**downstream untranscribed sequence**

**AAAGAAATAAAAATCAAACTTaagaaacgtgggtaagtcttgtgttatgg........**

**Coding sequence of the *mTrop1*-βGEO fusion cDNA**. Yellow highlight: *mTrop1* ORF (the leader sequence is underlined); grey highlight: βGEO ORF; primers are in magenta.

**mTrop1 EX2**

1 ATGGCGGGTCCCCAGGCCCTCGCGTTCGGGCTCCTGCTCGCGGTGGTCACAGCGACGCTG 60

1 [M](javascript:M(1)) [A](javascript:A(2)) [G](javascript:G(3)) [P](javascript:P(4)) [Q](javascript:Q(5)) [A](javascript:A(6)) [L](javascript:L(7)) [A](javascript:A(8)) [F](javascript:F(9)) [G](javascript:G(10)) [L](javascript:L(11)) [L](javascript:L(12)) [L](javascript:L(13)) [A](javascript:A(14)) [V](javascript:V(15)) [V](javascript:V(16)) [T](javascript:T(17)) [A](javascript:A(18)) [T](javascript:T(19)) [L](javascript:L(20)) 20

**mTrop1 EX3 Primer mT1Ex2F1**

61 GCCGCGGCTCAGAGAGACTGTGTCTGTGACAACTACAAGCTGGCAACAAGTTGCTCTCTG 120

21 [A](javascript:A(21)) [A](javascript:A(22)) [A](javascript:A(23)) [Q](javascript:Q(24)) [R](javascript:R(25)) [D](javascript:D(26)) [C](javascript:C(27)) [V](javascript:V(28)) [C](javascript:C(29)) [D](javascript:D(30)) [N](javascript:N(31)) [Y](javascript:Y(32)) [K](javascript:K(33)) [L](javascript:L(34)) [A](javascript:A(35)) [T](javascript:T(36)) [S](javascript:S(37)) [C](javascript:C(38)) [S](javascript:S(39)) [L](javascript:L(40)) 40

**Primer mT1Ex2F2**

121 AATGAATATGGTGAATGCCAGTGTACTTCCTATGGTACACAGAATACTGTCATTTGCTCC 180

41 [N](javascript:N(41)) [E](javascript:E(42)) [Y](javascript:Y(43)) [G](javascript:G(44)) [E](javascript:E(45)) [C](javascript:C(46)) [Q](javascript:Q(47)) [C](javascript:C(48)) [T](javascript:T(49)) [S](javascript:S(50)) [Y](javascript:Y(51)) [G](javascript:G(52)) [T](javascript:T(53)) [Q](javascript:Q(54)) [N](javascript:N(55)) [T](javascript:T(56)) [V](javascript:V(57)) [I](javascript:I(58)) [C](javascript:C(59)) [S](javascript:S(60)) 60

**βGEO Primer β-gal-Baygen-R3**

181 AAACGTCCCAGGTCCCGAAAACCAAAGAAGAAGAACGCAGATCGCAGATCGCAGATCCAG 240

61 [K](javascript:K(61)) [R](javascript:R(62)) [P](javascript:P(63)) [R](javascript:R(64)) [S](javascript:S(65)) [R](javascript:R(66)) [K](javascript:K(67)) [P](javascript:P(68)) [K](javascript:K(69)) [K](javascript:K(70)) [K](javascript:K(71)) [N](javascript:N(72)) [A](javascript:A(73)) [D](javascript:D(74)) [R](javascript:R(75)) [R](javascript:R(76)) [S](javascript:S(77)) [Q](javascript:Q(78)) [I](javascript:I(79)) [Q](javascript:Q(80)) 80

241 AAGTTCCTATTCCGAAGTTCCTATTCTCTAGAAAGTATAGGAACTTCTCAGATCTGCGGG 300

81 [K](javascript:K(81)) [F](javascript:F(82)) [L](javascript:L(83)) [F](javascript:F(84)) [R](javascript:R(85)) [S](javascript:S(86)) [S](javascript:S(87)) [Y](javascript:Y(88)) [S](javascript:S(89)) [L](javascript:L(90)) [E](javascript:E(91)) [S](javascript:S(92)) [I](javascript:I(93)) [G](javascript:G(94)) [T](javascript:T(95)) [S](javascript:S(96)) [Q](javascript:Q(97)) [I](javascript:I(98)) [C](javascript:C(99)) [G](javascript:G(100)) 100

**Primer β-gal-Baygen-R2**

301 CTGCAGGGAGAGTTGAGATGGAAGGCAGAGAAGGCTCCTTCTTCCCAGTCCTGGATCACC 360

101 [L](javascript:L(101)) [Q](javascript:Q(102)) [G](javascript:G(103)) [E](javascript:E(104)) [L](javascript:L(105)) [R](javascript:R(106)) [W](javascript:W(107)) [K](javascript:K(108)) [A](javascript:A(109)) [E](javascript:E(110)) [K](javascript:K(111)) [A](javascript:A(112)) [P](javascript:P(113)) [S](javascript:S(114)) [S](javascript:S(115)) [Q](javascript:Q(116)) [S](javascript:S(117)) [W](javascript:W(118)) [I](javascript:I(119)) [T](javascript:T(120)) 120

361 TTCTCCCTAAAGAACCAAAAGGTGTCTGTGCAGAAGTCTACTAGCAACCCCAAGTTCCAG 420

121 [F](javascript:F(121)) [S](javascript:S(122)) [L](javascript:L(123)) [K](javascript:K(124)) [N](javascript:N(125)) [Q](javascript:Q(126)) [K](javascript:K(127)) [V](javascript:V(128)) [S](javascript:S(129)) [V](javascript:V(130)) [Q](javascript:Q(131)) [K](javascript:K(132)) [S](javascript:S(133)) [T](javascript:T(134)) [S](javascript:S(135)) [N](javascript:N(136)) [P](javascript:P(137)) [K](javascript:K(138)) [F](javascript:F(139)) [Q](javascript:Q(140)) 140

421 CTGTCCGAAACGCTCCCACTCACCCTTCAGATACCCCAGGTCTCCCTTCAGTTTGCTGGT 480

141 [L](javascript:L(141)) [S](javascript:S(142)) [E](javascript:E(143)) [T](javascript:T(144)) [L](javascript:L(145)) [P](javascript:P(146)) [L](javascript:L(147)) [T](javascript:T(148)) [L](javascript:L(149)) [Q](javascript:Q(150)) [I](javascript:I(151)) [P](javascript:P(152)) [Q](javascript:Q(153)) [V](javascript:V(154)) [S](javascript:S(155)) [L](javascript:L(156)) [Q](javascript:Q(157)) [F](javascript:F(158)) [A](javascript:A(159)) [G](javascript:G(160)) 160

481 TCTGGCAACCTGACCCTGACTCTGGACAGAGGGATACTGTATCAGGAAGTGAACCTGGTG 540

161 [S](javascript:S(161)) [G](javascript:G(162)) [N](javascript:N(163)) [L](javascript:L(164)) [T](javascript:T(165)) [L](javascript:L(166)) [T](javascript:T(167)) [L](javascript:L(168)) [D](javascript:D(169)) [R](javascript:R(170)) [G](javascript:G(171)) [I](javascript:I(172)) [L](javascript:L(173)) [Y](javascript:Y(174)) [Q](javascript:Q(175)) [E](javascript:E(176)) [V](javascript:V(177)) [N](javascript:N(178)) [L](javascript:L(179)) [V](javascript:V(180)) 180

541 GTGATGAAAGTGACTCAGCCCGACAGCAACACTTTGACCTGTGAGGTGATGGGACCCACC 600

181 [V](javascript:V(181)) [M](javascript:M(182)) [K](javascript:K(183)) [V](javascript:V(184)) [T](javascript:T(185)) [Q](javascript:Q(186)) [P](javascript:P(187)) [D](javascript:D(188)) [S](javascript:S(189)) [N](javascript:N(190)) [T](javascript:T(191)) [L](javascript:L(192)) [T](javascript:T(193)) [C](javascript:C(194)) [E](javascript:E(195)) [V](javascript:V(196)) [M](javascript:M(197)) [G](javascript:G(198)) [P](javascript:P(199)) [T](javascript:T(200)) 200

601 TCACCCAAGATGAGACTGATCTTGAAGCAGGAGAATCAGGAGGCCAGGGTCTCCAGGCAG 660

201 [S](javascript:S(201)) [P](javascript:P(202)) [K](javascript:K(203)) [M](javascript:M(204)) [R](javascript:R(205)) [L](javascript:L(206)) [I](javascript:I(207)) [L](javascript:L(208)) [K](javascript:K(209)) [Q](javascript:Q(210)) [E](javascript:E(211)) [N](javascript:N(212)) [Q](javascript:Q(213)) [E](javascript:E(214)) [A](javascript:A(215)) [R](javascript:R(216)) [V](javascript:V(217)) [S](javascript:S(218)) [R](javascript:R(219)) [Q](javascript:Q(220)) 220

661 GAGAAAGTGATTCAAGTGCAGGCCCCTGAAGCAGGGGTGTGGCAATGTCTACTGAGTGAA 720

221 [E](javascript:E(221)) [K](javascript:K(222)) [V](javascript:V(223)) [I](javascript:I(224)) [Q](javascript:Q(225)) [V](javascript:V(226)) [Q](javascript:Q(227)) [A](javascript:A(228)) [P](javascript:P(229)) [E](javascript:E(230)) [A](javascript:A(231)) [G](javascript:G(232)) [V](javascript:V(233)) [W](javascript:W(234)) [Q](javascript:Q(235)) [C](javascript:C(236)) [L](javascript:L(237)) [L](javascript:L(238)) [S](javascript:S(239)) [E](javascript:E(240)) 240

721 GGTGAAGAGGTCAAGATGGACTCCAAGATCCAGGTTTTATCCAAAGGGTTGAACCAGACA 780

241 [G](javascript:G(241)) [E](javascript:E(242)) [E](javascript:E(243)) [V](javascript:V(244)) [K](javascript:K(245)) [M](javascript:M(246)) [D](javascript:D(247)) [S](javascript:S(248)) [K](javascript:K(249)) [I](javascript:I(250)) [Q](javascript:Q(251)) [V](javascript:V(252)) [L](javascript:L(253)) [S](javascript:S(254)) [K](javascript:K(255)) [G](javascript:G(256)) [L](javascript:L(257)) [N](javascript:N(258)) [Q](javascript:Q(259)) [T](javascript:T(260)) 260

781 ATGTTCCTGGCTGTCGTGCTGGGGAGCGCCTTCAGCTTTCTGGTTTTCACGGGGCTCTGC 840

261 [M](javascript:M(261)) [F](javascript:F(262)) [L](javascript:L(263)) [A](javascript:A(264)) [V](javascript:V(265)) [V](javascript:V(266)) [L](javascript:L(267)) [G](javascript:G(268)) [S](javascript:S(269)) [A](javascript:A(270)) [F](javascript:F(271)) [S](javascript:S(272)) [F](javascript:F(273)) [L](javascript:L(274)) [V](javascript:V(275)) [F](javascript:F(276)) [T](javascript:T(277)) [G](javascript:G(278)) [L](javascript:L(279)) [C](javascript:C(280)) 280

841 ATCCTATTCTGTGTCAGGTGCCGGCACCAACAGCGCCAGGCAGCACGGATGTCTCAGATC 900

281 [I](javascript:I(281)) [L](javascript:L(282)) [F](javascript:F(283)) [C](javascript:C(284)) [V](javascript:V(285)) [R](javascript:R(286)) [C](javascript:C(287)) [R](javascript:R(288)) [H](javascript:H(289)) [Q](javascript:Q(290)) [Q](javascript:Q(291)) [R](javascript:R(292)) [Q](javascript:Q(293)) [A](javascript:A(294)) [A](javascript:A(295)) [R](javascript:R(296)) [M](javascript:M(297)) [S](javascript:S(298)) [Q](javascript:Q(299)) [I](javascript:I(300)) 300

901 AAGAGGCTTCTCAGTGAGAAGAAGACTTGCCAGTGCTCCCACCGGATGCAGAAAAGCCAC 960

301 [K](javascript:K(301)) [R](javascript:R(302)) [L](javascript:L(303)) [L](javascript:L(304)) [S](javascript:S(305)) [E](javascript:E(306)) [K](javascript:K(307)) [K](javascript:K(308)) [T](javascript:T(309)) [C](javascript:C(310)) [Q](javascript:Q(311)) [C](javascript:C(312)) [S](javascript:S(313)) [H](javascript:H(314)) [R](javascript:R(315)) [M](javascript:M(316)) [Q](javascript:Q(317)) [K](javascript:K(318)) [S](javascript:S(319)) [H](javascript:H(320)) 320

961 AATCTCATATATGGGAGCGATGATCCCGTCGTTTTACAACGTCGTGACTGGGAAAACCCT 1020

321 [N](javascript:N(321)) [L](javascript:L(322)) [I](javascript:I(323)) [Y](javascript:Y(324)) [G](javascript:G(325)) [S](javascript:S(326)) [D](javascript:D(327)) [D](javascript:D(328)) [P](javascript:P(329)) [V](javascript:V(330)) [V](javascript:V(331)) [L](javascript:L(332)) [Q](javascript:Q(333)) [R](javascript:R(334)) [R](javascript:R(335)) [D](javascript:D(336)) [W](javascript:W(337)) [E](javascript:E(338)) [N](javascript:N(339)) [P](javascript:P(340)) 340

1021 GGCGTTACCCAACTTAATCGCCTTGCAGCACATCCCCCTTTCGCCAGCTGGCGTAATAGC 1080

341 [G](javascript:G(341)) [V](javascript:V(342)) [T](javascript:T(343)) [Q](javascript:Q(344)) [L](javascript:L(345)) [N](javascript:N(346)) [R](javascript:R(347)) [L](javascript:L(348)) [A](javascript:A(349)) [A](javascript:A(350)) [H](javascript:H(351)) [P](javascript:P(352)) [P](javascript:P(353)) [F](javascript:F(354)) [A](javascript:A(355)) [S](javascript:S(356)) [W](javascript:W(357)) [R](javascript:R(358)) [N](javascript:N(359)) [S](javascript:S(360)) 360

1081 GAAGAGGCCCGCACCGATCGCCCTTCCCAACAGTTGCGCAGCCTGAATGGCGAATGGCGC 1140

361 [E](javascript:E(361)) [E](javascript:E(362)) [A](javascript:A(363)) [R](javascript:R(364)) [T](javascript:T(365)) [D](javascript:D(366)) [R](javascript:R(367)) [P](javascript:P(368)) [S](javascript:S(369)) [Q](javascript:Q(370)) [Q](javascript:Q(371)) [L](javascript:L(372)) [R](javascript:R(373)) [S](javascript:S(374)) [L](javascript:L(375)) [N](javascript:N(376)) [G](javascript:G(377)) [E](javascript:E(378)) [W](javascript:W(379)) [R](javascript:R(380)) 380

1141 TTTGCCTGGTTTCCGGCACCAGAAGCGGTGCCGGAAAGCTGGCTGGAGTGCGATCTTCCT 1200

381 [F](javascript:F(381)) [A](javascript:A(382)) [W](javascript:W(383)) [F](javascript:F(384)) [P](javascript:P(385)) [A](javascript:A(386)) [P](javascript:P(387)) [E](javascript:E(388)) [A](javascript:A(389)) [V](javascript:V(390)) [P](javascript:P(391)) [E](javascript:E(392)) [S](javascript:S(393)) [W](javascript:W(394)) [L](javascript:L(395)) [E](javascript:E(396)) [C](javascript:C(397)) [D](javascript:D(398)) [L](javascript:L(399)) [P](javascript:P(400)) 400

1201 GAGGCCGATACTGTCGTCGTCCCCTCAAACTGGCAGATGCACGGTTACGATGCGCCCATC 1260

401 [E](javascript:E(401)) [A](javascript:A(402)) [D](javascript:D(403)) [T](javascript:T(404)) [V](javascript:V(405)) [V](javascript:V(406)) [V](javascript:V(407)) [P](javascript:P(408)) [S](javascript:S(409)) [N](javascript:N(410)) [W](javascript:W(411)) [Q](javascript:Q(412)) [M](javascript:M(413)) [H](javascript:H(414)) [G](javascript:G(415)) [Y](javascript:Y(416)) [D](javascript:D(417)) [A](javascript:A(418)) [P](javascript:P(419)) [I](javascript:I(420)) 420

1261 TACACCAACGTAACCTATCCCATTACGGTCAATCCGCCGTTTGTTCCCACGGAGAATCCG 1320

421 [Y](javascript:Y(421)) [T](javascript:T(422)) [N](javascript:N(423)) [V](javascript:V(424)) [T](javascript:T(425)) [Y](javascript:Y(426)) [P](javascript:P(427)) [I](javascript:I(428)) [T](javascript:T(429)) [V](javascript:V(430)) [N](javascript:N(431)) [P](javascript:P(432)) [P](javascript:P(433)) [F](javascript:F(434)) [V](javascript:V(435)) [P](javascript:P(436)) [T](javascript:T(437)) [E](javascript:E(438)) [N](javascript:N(439)) [P](javascript:P(440)) 440

1321 ACGGGTTGTTACTCGCTCACATTTAATGTTGATGAAAGCTGGCTACAGGAAGGCCAGACG 1380

441 [T](javascript:T(441)) [G](javascript:G(442)) [C](javascript:C(443)) [Y](javascript:Y(444)) [S](javascript:S(445)) [L](javascript:L(446)) [T](javascript:T(447)) [F](javascript:F(448)) [N](javascript:N(449)) [V](javascript:V(450)) [D](javascript:D(451)) [E](javascript:E(452)) [S](javascript:S(453)) [W](javascript:W(454)) [L](javascript:L(455)) [Q](javascript:Q(456)) [E](javascript:E(457)) [G](javascript:G(458)) [Q](javascript:Q(459)) [T](javascript:T(460)) 460

1381 CGAATTATTTTTGATGGCGTTAACTCGGCGTTTCATCTGTGGTGCAACGGGCGCTGGGTC 1440

461 [R](javascript:R(461)) [I](javascript:I(462)) [I](javascript:I(463)) [F](javascript:F(464)) [D](javascript:D(465)) [G](javascript:G(466)) [V](javascript:V(467)) [N](javascript:N(468)) [S](javascript:S(469)) [A](javascript:A(470)) [F](javascript:F(471)) [H](javascript:H(472)) [L](javascript:L(473)) [W](javascript:W(474)) [C](javascript:C(475)) [N](javascript:N(476)) [G](javascript:G(477)) [R](javascript:R(478)) [W](javascript:W(479)) [V](javascript:V(480)) 480

1441 GGTTACGGCCAGGACAGTCGTTTGCCGTCTGAATTTGACCTGAGCGCATTTTTACGCGCC 1500

481 [G](javascript:G(481)) [Y](javascript:Y(482)) [G](javascript:G(483)) [Q](javascript:Q(484)) [D](javascript:D(485)) [S](javascript:S(486)) [R](javascript:R(487)) [L](javascript:L(488)) [P](javascript:P(489)) [S](javascript:S(490)) [E](javascript:E(491)) [F](javascript:F(492)) [D](javascript:D(493)) [L](javascript:L(494)) [S](javascript:S(495)) [A](javascript:A(496)) [F](javascript:F(497)) [L](javascript:L(498)) [R](javascript:R(499)) [A](javascript:A(500)) 500

1501 GGAGAAAACCGCCTCGCGGTGATGGTGCTGCGTTGGAGTGACGGCAGTTATCTGGAAGAT 1560

501 [G](javascript:G(501)) [E](javascript:E(502)) [N](javascript:N(503)) [R](javascript:R(504)) [L](javascript:L(505)) [A](javascript:A(506)) [V](javascript:V(507)) [M](javascript:M(508)) [V](javascript:V(509)) [L](javascript:L(510)) [R](javascript:R(511)) [W](javascript:W(512)) [S](javascript:S(513)) [D](javascript:D(514)) [G](javascript:G(515)) [S](javascript:S(516)) [Y](javascript:Y(517)) [L](javascript:L(518)) [E](javascript:E(519)) [D](javascript:D(520)) 520

1561 CAGGATATGTGGCGGATGAGCGGCATTTTCCGTGACGTCTCGTTGCTGCATAAACCGACT 1620

521 [Q](javascript:Q(521)) [D](javascript:D(522)) [M](javascript:M(523)) [W](javascript:W(524)) [R](javascript:R(525)) [M](javascript:M(526)) [S](javascript:S(527)) [G](javascript:G(528)) [I](javascript:I(529)) [F](javascript:F(530)) [R](javascript:R(531)) [D](javascript:D(532)) [V](javascript:V(533)) [S](javascript:S(534)) [L](javascript:L(535)) [L](javascript:L(536)) [H](javascript:H(537)) [K](javascript:K(538)) [P](javascript:P(539)) [T](javascript:T(540)) 540

1621 ACACAAATCAGCGATTTCCATGTTGCCACTCGCTTTAATGATGATTTCAGCCGCGCTGTA 1680

541 [T](javascript:T(541)) [Q](javascript:Q(542)) [I](javascript:I(543)) [S](javascript:S(544)) [D](javascript:D(545)) [F](javascript:F(546)) [H](javascript:H(547)) [V](javascript:V(548)) [A](javascript:A(549)) [T](javascript:T(550)) [R](javascript:R(551)) [F](javascript:F(552)) [N](javascript:N(553)) [D](javascript:D(554)) [D](javascript:D(555)) [F](javascript:F(556)) [S](javascript:S(557)) [R](javascript:R(558)) [A](javascript:A(559)) [V](javascript:V(560)) 560

1681 CTGGAGGCTGAAGTTCAGATGTGCGGCGAGTTGCGTGACTACCTACGGGTAACAGTTTCT 1740

561 [L](javascript:L(561)) [E](javascript:E(562)) [A](javascript:A(563)) [E](javascript:E(564)) [V](javascript:V(565)) [Q](javascript:Q(566)) [M](javascript:M(567)) [C](javascript:C(568)) [G](javascript:G(569)) [E](javascript:E(570)) [L](javascript:L(571)) [R](javascript:R(572)) [D](javascript:D(573)) [Y](javascript:Y(574)) [L](javascript:L(575)) [R](javascript:R(576)) [V](javascript:V(577)) [T](javascript:T(578)) [V](javascript:V(579)) [S](javascript:S(580)) 580

1741 TTATGGCAGGGTGAAACGCAGGTCGCCAGCGGCACCGCGCCTTTCGGCGGTGAAATTATC 1800

581 [L](javascript:L(581)) [W](javascript:W(582)) [Q](javascript:Q(583)) [G](javascript:G(584)) [E](javascript:E(585)) [T](javascript:T(586)) [Q](javascript:Q(587)) [V](javascript:V(588)) [A](javascript:A(589)) [S](javascript:S(590)) [G](javascript:G(591)) [T](javascript:T(592)) [A](javascript:A(593)) [P](javascript:P(594)) [F](javascript:F(595)) [G](javascript:G(596)) [G](javascript:G(597)) [E](javascript:E(598)) [I](javascript:I(599)) [I](javascript:I(600)) 600

1801 GATGAGCGTGGTGGTTATGCCGATCGCGTCACACTACGTCTGAACGTCGAAAACCCGAAA 1860

601 [D](javascript:D(601)) [E](javascript:E(602)) [R](javascript:R(603)) [G](javascript:G(604)) [G](javascript:G(605)) [Y](javascript:Y(606)) [A](javascript:A(607)) [D](javascript:D(608)) [R](javascript:R(609)) [V](javascript:V(610)) [T](javascript:T(611)) [L](javascript:L(612)) [R](javascript:R(613)) [L](javascript:L(614)) [N](javascript:N(615)) [V](javascript:V(616)) [E](javascript:E(617)) [N](javascript:N(618)) [P](javascript:P(619)) [K](javascript:K(620)) 620

1861 CTGTGGAGCGCCGAAATCCCGAATCTCTATCGTGCGGTGGTTGAACTGCACACCGCCGAC 1920

621 [L](javascript:L(621)) [W](javascript:W(622)) [S](javascript:S(623)) [A](javascript:A(624)) [E](javascript:E(625)) [I](javascript:I(626)) [P](javascript:P(627)) [N](javascript:N(628)) [L](javascript:L(629)) [Y](javascript:Y(630)) [R](javascript:R(631)) [A](javascript:A(632)) [V](javascript:V(633)) [V](javascript:V(634)) [E](javascript:E(635)) [L](javascript:L(636)) [H](javascript:H(637)) [T](javascript:T(638)) [A](javascript:A(639)) [D](javascript:D(640)) 640

1921 GGCACGCTGATTGAAGCAGAAGCCTGCGATGTCGGTTTCCGCGAGGTGCGGATTGAAAAT 1980

641 [G](javascript:G(641)) [T](javascript:T(642)) [L](javascript:L(643)) [I](javascript:I(644)) [E](javascript:E(645)) [A](javascript:A(646)) [E](javascript:E(647)) [A](javascript:A(648)) [C](javascript:C(649)) [D](javascript:D(650)) [V](javascript:V(651)) [G](javascript:G(652)) [F](javascript:F(653)) [R](javascript:R(654)) [E](javascript:E(655)) [V](javascript:V(656)) [R](javascript:R(657)) [I](javascript:I(658)) [E](javascript:E(659)) [N](javascript:N(660)) 660

1981 GGTCTGCTGCTGCTGAACGGCAAGCCGTTGCTGATTCGAGGCGTTAACCGTCACGAGCAT 2040

661 [G](javascript:G(661)) [L](javascript:L(662)) [L](javascript:L(663)) [L](javascript:L(664)) [L](javascript:L(665)) [N](javascript:N(666)) [G](javascript:G(667)) [K](javascript:K(668)) [P](javascript:P(669)) [L](javascript:L(670)) [L](javascript:L(671)) [I](javascript:I(672)) [R](javascript:R(673)) [G](javascript:G(674)) [V](javascript:V(675)) [N](javascript:N(676)) [R](javascript:R(677)) [H](javascript:H(678)) [E](javascript:E(679)) [H](javascript:H(680)) 680

2041 CATCCTCTGCATGGTCAGGTCATGGATGAGCAGACGATGGTGCAGGATATCCTGCTGATG 2100

681 [H](javascript:H(681)) [P](javascript:P(682)) [L](javascript:L(683)) [H](javascript:H(684)) [G](javascript:G(685)) [Q](javascript:Q(686)) [V](javascript:V(687)) [M](javascript:M(688)) [D](javascript:D(689)) [E](javascript:E(690)) [Q](javascript:Q(691)) [T](javascript:T(692)) [M](javascript:M(693)) [V](javascript:V(694)) [Q](javascript:Q(695)) [D](javascript:D(696)) [I](javascript:I(697)) [L](javascript:L(698)) [L](javascript:L(699)) [M](javascript:M(700)) 700

2101 AAGCAGAACAACTTTAACGCCGTGCGCTGTTCGCATTATCCGAACCATCCGCTGTGGTAC 2160

701 [K](javascript:K(701)) [Q](javascript:Q(702)) [N](javascript:N(703)) [N](javascript:N(704)) [F](javascript:F(705)) [N](javascript:N(706)) [A](javascript:A(707)) [V](javascript:V(708)) [R](javascript:R(709)) [C](javascript:C(710)) [S](javascript:S(711)) [H](javascript:H(712)) [Y](javascript:Y(713)) [P](javascript:P(714)) [N](javascript:N(715)) [H](javascript:H(716)) [P](javascript:P(717)) [L](javascript:L(718)) [W](javascript:W(719)) [Y](javascript:Y(720)) 720

2161 ACGCTGTGCGACCGCTACGGCCTGTATGTGGTGGATGAAGCCAATATTGAAACCCACGGC 2220

721 [T](javascript:T(721)) [L](javascript:L(722)) [C](javascript:C(723)) [D](javascript:D(724)) [R](javascript:R(725)) [Y](javascript:Y(726)) [G](javascript:G(727)) [L](javascript:L(728)) [Y](javascript:Y(729)) [V](javascript:V(730)) [V](javascript:V(731)) [D](javascript:D(732)) [E](javascript:E(733)) [A](javascript:A(734)) [N](javascript:N(735)) [I](javascript:I(736)) [E](javascript:E(737)) [T](javascript:T(738)) [H](javascript:H(739)) [G](javascript:G(740)) 740

2221 ATGGTGCCAATGAATCGTCTGACCGATGATCCGCGCTGGCTACCGGCGATGAGCGAACGC 2280

741 [M](javascript:M(741)) [V](javascript:V(742)) [P](javascript:P(743)) [M](javascript:M(744)) [N](javascript:N(745)) [R](javascript:R(746)) [L](javascript:L(747)) [T](javascript:T(748)) [D](javascript:D(749)) [D](javascript:D(750)) [P](javascript:P(751)) [R](javascript:R(752)) [W](javascript:W(753)) [L](javascript:L(754)) [P](javascript:P(755)) [A](javascript:A(756)) [M](javascript:M(757)) [S](javascript:S(758)) [E](javascript:E(759)) [R](javascript:R(760)) 760

2281 GTAACGCGAATGGTGCAGCGCGATCGTAATCACCCGAGTGTGATCATCTGGTCGCTGGGG 2340

761 [V](javascript:V(761)) [T](javascript:T(762)) [R](javascript:R(763)) [M](javascript:M(764)) [V](javascript:V(765)) [Q](javascript:Q(766)) [R](javascript:R(767)) [D](javascript:D(768)) [R](javascript:R(769)) [N](javascript:N(770)) [H](javascript:H(771)) [P](javascript:P(772)) [S](javascript:S(773)) [V](javascript:V(774)) [I](javascript:I(775)) [I](javascript:I(776)) [W](javascript:W(777)) [S](javascript:S(778)) [L](javascript:L(779)) [G](javascript:G(780)) 780

2341 AATGAATCAGGCCACGGCGCTAATCACGACGCGCTGTATCGCTGGATCAAATCTGTCGAT 2400

781 [N](javascript:N(781)) [E](javascript:E(782)) [S](javascript:S(783)) [G](javascript:G(784)) [H](javascript:H(785)) [G](javascript:G(786)) [A](javascript:A(787)) [N](javascript:N(788)) [H](javascript:H(789)) [D](javascript:D(790)) [A](javascript:A(791)) [L](javascript:L(792)) [Y](javascript:Y(793)) [R](javascript:R(794)) [W](javascript:W(795)) [I](javascript:I(796)) [K](javascript:K(797)) [S](javascript:S(798)) [V](javascript:V(799)) [D](javascript:D(800)) 800

2401 CCTTCCCGCCCGGTGCAGTATGAAGGCGGCGGAGCCGACACCACGGCCACCGATATTATT 2460

801 [P](javascript:P(801)) [S](javascript:S(802)) [R](javascript:R(803)) [P](javascript:P(804)) [V](javascript:V(805)) [Q](javascript:Q(806)) [Y](javascript:Y(807)) [E](javascript:E(808)) [G](javascript:G(809)) [G](javascript:G(810)) [G](javascript:G(811)) [A](javascript:A(812)) [D](javascript:D(813)) [T](javascript:T(814)) [T](javascript:T(815)) [A](javascript:A(816)) [T](javascript:T(817)) [D](javascript:D(818)) [I](javascript:I(819)) [I](javascript:I(820)) 820

2461 TGCCCGATGTACGCGCGCGTGGATGAAGACCAGCCCTTCCCGGCTGTGCCGAAATGGTCC 2520

821 [C](javascript:C(821)) [P](javascript:P(822)) [M](javascript:M(823)) [Y](javascript:Y(824)) [A](javascript:A(825)) [R](javascript:R(826)) [V](javascript:V(827)) [D](javascript:D(828)) [E](javascript:E(829)) [D](javascript:D(830)) [Q](javascript:Q(831)) [P](javascript:P(832)) [F](javascript:F(833)) [P](javascript:P(834)) [A](javascript:A(835)) [V](javascript:V(836)) [P](javascript:P(837)) [K](javascript:K(838)) [W](javascript:W(839)) [S](javascript:S(840)) 840

2521 ATCAAAAAATGGCTTTCGCTACCTGGAGAGACGCGCCCGCTGATCCTTTGCGAATACGCC 2580

841 [I](javascript:I(841)) [K](javascript:K(842)) [K](javascript:K(843)) [W](javascript:W(844)) [L](javascript:L(845)) [S](javascript:S(846)) [L](javascript:L(847)) [P](javascript:P(848)) [G](javascript:G(849)) [E](javascript:E(850)) [T](javascript:T(851)) [R](javascript:R(852)) [P](javascript:P(853)) [L](javascript:L(854)) [I](javascript:I(855)) [L](javascript:L(856)) [C](javascript:C(857)) [E](javascript:E(858)) [Y](javascript:Y(859)) [A](javascript:A(860)) 860

2581 CACGCGATGGGTAACAGTCTTGGCGGTTTCGCTAAATACTGGCAGGCGTTTCGTCAGTAT 2640

861 [H](javascript:H(861)) [A](javascript:A(862)) [M](javascript:M(863)) [G](javascript:G(864)) [N](javascript:N(865)) [S](javascript:S(866)) [L](javascript:L(867)) [G](javascript:G(868)) [G](javascript:G(869)) [F](javascript:F(870)) [A](javascript:A(871)) [K](javascript:K(872)) [Y](javascript:Y(873)) [W](javascript:W(874)) [Q](javascript:Q(875)) [A](javascript:A(876)) [F](javascript:F(877)) [R](javascript:R(878)) [Q](javascript:Q(879)) [Y](javascript:Y(880)) 880

2641 CCCCGTTTACAGGGCGGCTTCGTCTGGGACTGGGTGGATCAGTCGCTGATTAAATATGAT 2700

881 [P](javascript:P(881)) [R](javascript:R(882)) [L](javascript:L(883)) [Q](javascript:Q(884)) [G](javascript:G(885)) [G](javascript:G(886)) [F](javascript:F(887)) [V](javascript:V(888)) [W](javascript:W(889)) [D](javascript:D(890)) [W](javascript:W(891)) [V](javascript:V(892)) [D](javascript:D(893)) [Q](javascript:Q(894)) [S](javascript:S(895)) [L](javascript:L(896)) [I](javascript:I(897)) [K](javascript:K(898)) [Y](javascript:Y(899)) [D](javascript:D(900)) 900

2701 GAAAACGGCAACCCGTGGTCGGCTTACGGCGGTGATTTTGGCGATACGCCGAACGATCGC 2760

901 [E](javascript:E(901)) [N](javascript:N(902)) [G](javascript:G(903)) [N](javascript:N(904)) [P](javascript:P(905)) [W](javascript:W(906)) [S](javascript:S(907)) [A](javascript:A(908)) [Y](javascript:Y(909)) [G](javascript:G(910)) [G](javascript:G(911)) [D](javascript:D(912)) [F](javascript:F(913)) [G](javascript:G(914)) [D](javascript:D(915)) [T](javascript:T(916)) [P](javascript:P(917)) [N](javascript:N(918)) [D](javascript:D(919)) [R](javascript:R(920)) 920

2761 CAGTTCTGTATGAACGGTCTGGTCTTTGCCGACCGCACGCCGCATCCAGCGCTGACGGAA 2820

921 [Q](javascript:Q(921)) [F](javascript:F(922)) [C](javascript:C(923)) [M](javascript:M(924)) [N](javascript:N(925)) [G](javascript:G(926)) [L](javascript:L(927)) [V](javascript:V(928)) [F](javascript:F(929)) [A](javascript:A(930)) [D](javascript:D(931)) [R](javascript:R(932)) [T](javascript:T(933)) [P](javascript:P(934)) [H](javascript:H(935)) [P](javascript:P(936)) [A](javascript:A(937)) [L](javascript:L(938)) [T](javascript:T(939)) [E](javascript:E(940)) 940

2821 GCAAAACACCAGCAGCAGTTTTTCCAGTTCCGTTTATCCGGGCAAACCATCGAAGTGACC 2880

941 [A](javascript:A(941)) [K](javascript:K(942)) [H](javascript:H(943)) [Q](javascript:Q(944)) [Q](javascript:Q(945)) [Q](javascript:Q(946)) [F](javascript:F(947)) [F](javascript:F(948)) [Q](javascript:Q(949)) [F](javascript:F(950)) [R](javascript:R(951)) [L](javascript:L(952)) [S](javascript:S(953)) [G](javascript:G(954)) [Q](javascript:Q(955)) [T](javascript:T(956)) [I](javascript:I(957)) [E](javascript:E(958)) [V](javascript:V(959)) [T](javascript:T(960)) 960

2881 AGCGAATACCTGTTCCGTCATAGCGATAACGAGCTCCTGCACTGGATGGTGGCGCTGGAT 2940

961 [S](javascript:S(961)) [E](javascript:E(962)) [Y](javascript:Y(963)) [L](javascript:L(964)) [F](javascript:F(965)) [R](javascript:R(966)) [H](javascript:H(967)) [S](javascript:S(968)) [D](javascript:D(969)) [N](javascript:N(970)) [E](javascript:E(971)) [L](javascript:L(972)) [L](javascript:L(973)) [H](javascript:H(974)) [W](javascript:W(975)) [M](javascript:M(976)) [V](javascript:V(977)) [A](javascript:A(978)) [L](javascript:L(979)) [D](javascript:D(980)) 980

2941 GGTAAGCCGCTGGCAAGCGGTGAAGTGCCTCTGGATGTCGCTCCACAAGGTAAACAGTTG 3000

981 [G](javascript:G(981)) [K](javascript:K(982)) [P](javascript:P(983)) [L](javascript:L(984)) [A](javascript:A(985)) [S](javascript:S(986)) [G](javascript:G(987)) [E](javascript:E(988)) [V](javascript:V(989)) [P](javascript:P(990)) [L](javascript:L(991)) [D](javascript:D(992)) [V](javascript:V(993)) [A](javascript:A(994)) [P](javascript:P(995)) [Q](javascript:Q(996)) [G](javascript:G(997)) [K](javascript:K(998)) [Q](javascript:Q(999)) [L](javascript:L(1000)) 1000

3001 ATTGAACTGCCTGAACTACCGCAGCCGGAGAGCGCCGGGCAACTCTGGCTCACAGTACGC 3060

1001 [I](javascript:I(1001)) [E](javascript:E(1002)) [L](javascript:L(1003)) [P](javascript:P(1004)) [E](javascript:E(1005)) [L](javascript:L(1006)) [P](javascript:P(1007)) [Q](javascript:Q(1008)) [P](javascript:P(1009)) [E](javascript:E(1010)) [S](javascript:S(1011)) [A](javascript:A(1012)) [G](javascript:G(1013)) [Q](javascript:Q(1014)) [L](javascript:L(1015)) [W](javascript:W(1016)) [L](javascript:L(1017)) [T](javascript:T(1018)) [V](javascript:V(1019)) [R](javascript:R(1020)) 1020

3061 GTAGTGCAACCGAACGCGACCGCATGGTCAGAAGCCGGGCACATCAGCGCCTGGCAGCAG 3120

1021 [V](javascript:V(1021)) [V](javascript:V(1022)) [Q](javascript:Q(1023)) [P](javascript:P(1024)) [N](javascript:N(1025)) [A](javascript:A(1026)) [T](javascript:T(1027)) [A](javascript:A(1028)) [W](javascript:W(1029)) [S](javascript:S(1030)) [E](javascript:E(1031)) [A](javascript:A(1032)) [G](javascript:G(1033)) [H](javascript:H(1034)) [I](javascript:I(1035)) [S](javascript:S(1036)) [A](javascript:A(1037)) [W](javascript:W(1038)) [Q](javascript:Q(1039)) [Q](javascript:Q(1040)) 1040

3121 TGGCGTCTGGCGGAAAACCTCAGTGTGACGCTCCCCGCCGCGTCCCACGCCATCCCGCAT 3180

1041 [W](javascript:W(1041)) [R](javascript:R(1042)) [L](javascript:L(1043)) [A](javascript:A(1044)) [E](javascript:E(1045)) [N](javascript:N(1046)) [L](javascript:L(1047)) [S](javascript:S(1048)) [V](javascript:V(1049)) [T](javascript:T(1050)) [L](javascript:L(1051)) [P](javascript:P(1052)) [A](javascript:A(1053)) [A](javascript:A(1054)) [S](javascript:S(1055)) [H](javascript:H(1056)) [A](javascript:A(1057)) [I](javascript:I(1058)) [P](javascript:P(1059)) [H](javascript:H(1060)) 1060

3181 CTGACCACCAGCGAAATGGATTTTTGCATCGAGCTGGGTAATAAGCGTTGGCAATTTAAC 3240

1061 [L](javascript:L(1061)) [T](javascript:T(1062)) [T](javascript:T(1063)) [S](javascript:S(1064)) [E](javascript:E(1065)) [M](javascript:M(1066)) [D](javascript:D(1067)) [F](javascript:F(1068)) [C](javascript:C(1069)) [I](javascript:I(1070)) [E](javascript:E(1071)) [L](javascript:L(1072)) [G](javascript:G(1073)) [N](javascript:N(1074)) [K](javascript:K(1075)) [R](javascript:R(1076)) [W](javascript:W(1077)) [Q](javascript:Q(1078)) [F](javascript:F(1079)) [N](javascript:N(1080)) 1080

3241 CGCCAGTCAGGCTTTCTTTCACAGATGTGGATTGGCGATAAAAAACAACTGCTGACGCCG 3300

1081 [R](javascript:R(1081)) [Q](javascript:Q(1082)) [S](javascript:S(1083)) [G](javascript:G(1084)) [F](javascript:F(1085)) [L](javascript:L(1086)) [S](javascript:S(1087)) [Q](javascript:Q(1088)) [M](javascript:M(1089)) [W](javascript:W(1090)) [I](javascript:I(1091)) [G](javascript:G(1092)) [D](javascript:D(1093)) [K](javascript:K(1094)) [K](javascript:K(1095)) [Q](javascript:Q(1096)) [L](javascript:L(1097)) [L](javascript:L(1098)) [T](javascript:T(1099)) [P](javascript:P(1100)) 1100

3301 CTGCGCGATCAGTTCACCCGTGCACCGCTGGATAACGACATTGGCGTAAGTGAAGCGACC 3360

1101 [L](javascript:L(1101)) [R](javascript:R(1102)) [D](javascript:D(1103)) [Q](javascript:Q(1104)) [F](javascript:F(1105)) [T](javascript:T(1106)) [R](javascript:R(1107)) [A](javascript:A(1108)) [P](javascript:P(1109)) [L](javascript:L(1110)) [D](javascript:D(1111)) [N](javascript:N(1112)) [D](javascript:D(1113)) [I](javascript:I(1114)) [G](javascript:G(1115)) [V](javascript:V(1116)) [S](javascript:S(1117)) [E](javascript:E(1118)) [A](javascript:A(1119)) [T](javascript:T(1120)) 1120

3361 CGCATTGACCCTAACGCCTGGGTCGAACGCTGGAAGGCGGCGGGCCATTACCAGGCCGAA 3420

1121 [R](javascript:R(1121)) [I](javascript:I(1122)) [D](javascript:D(1123)) [P](javascript:P(1124)) [N](javascript:N(1125)) [A](javascript:A(1126)) [W](javascript:W(1127)) [V](javascript:V(1128)) [E](javascript:E(1129)) [R](javascript:R(1130)) [W](javascript:W(1131)) [K](javascript:K(1132)) [A](javascript:A(1133)) [A](javascript:A(1134)) [G](javascript:G(1135)) [H](javascript:H(1136)) [Y](javascript:Y(1137)) [Q](javascript:Q(1138)) [A](javascript:A(1139)) [E](javascript:E(1140)) 1140

3421 GCAGCGTTGTTGCAGTGCACGGCAGATACACTTGCTGATGCGGTGCTGATTACGACCGCT 3480

1141 [A](javascript:A(1141)) [A](javascript:A(1142)) [L](javascript:L(1143)) [L](javascript:L(1144)) [Q](javascript:Q(1145)) [C](javascript:C(1146)) [T](javascript:T(1147)) [A](javascript:A(1148)) [D](javascript:D(1149)) [T](javascript:T(1150)) [L](javascript:L(1151)) [A](javascript:A(1152)) [D](javascript:D(1153)) [A](javascript:A(1154)) [V](javascript:V(1155)) [L](javascript:L(1156)) [I](javascript:I(1157)) [T](javascript:T(1158)) [T](javascript:T(1159)) [A](javascript:A(1160)) 1160

3481 CACGCGTGGCAGCATCAGGGGAAAACCTTATTTATCAGCCGGAAAACCTACCGGATTGAT 3540

1161 [H](javascript:H(1161)) [A](javascript:A(1162)) [W](javascript:W(1163)) [Q](javascript:Q(1164)) [H](javascript:H(1165)) [Q](javascript:Q(1166)) [G](javascript:G(1167)) [K](javascript:K(1168)) [T](javascript:T(1169)) [L](javascript:L(1170)) [F](javascript:F(1171)) [I](javascript:I(1172)) [S](javascript:S(1173)) [R](javascript:R(1174)) [K](javascript:K(1175)) [T](javascript:T(1176)) [Y](javascript:Y(1177)) [R](javascript:R(1178)) [I](javascript:I(1179)) [D](javascript:D(1180)) 1180

3541 GGTAGTGGTCAAATGGCGATTACCGTTGATGTTGAAGTGGCGAGCGATACACCGCATCCG 3600

1181 [G](javascript:G(1181)) [S](javascript:S(1182)) [G](javascript:G(1183)) [Q](javascript:Q(1184)) [M](javascript:M(1185)) [A](javascript:A(1186)) [I](javascript:I(1187)) [T](javascript:T(1188)) [V](javascript:V(1189)) [D](javascript:D(1190)) [V](javascript:V(1191)) [E](javascript:E(1192)) [V](javascript:V(1193)) [A](javascript:A(1194)) [S](javascript:S(1195)) [D](javascript:D(1196)) [T](javascript:T(1197)) [P](javascript:P(1198)) [H](javascript:H(1199)) [P](javascript:P(1200)) 1200

3601 GCGCGGATTGGCCTGAACTGCCAGCTGGCGCAGGTAGCAGAGCGGGTAAACTGGCTCGGA 3660

1201 [A](javascript:A(1201)) [R](javascript:R(1202)) [I](javascript:I(1203)) [G](javascript:G(1204)) [L](javascript:L(1205)) [N](javascript:N(1206)) [C](javascript:C(1207)) [Q](javascript:Q(1208)) [L](javascript:L(1209)) [A](javascript:A(1210)) [Q](javascript:Q(1211)) [V](javascript:V(1212)) [A](javascript:A(1213)) [E](javascript:E(1214)) [R](javascript:R(1215)) [V](javascript:V(1216)) [N](javascript:N(1217)) [W](javascript:W(1218)) [L](javascript:L(1219)) [G](javascript:G(1220)) 1220

3661 TTAGGGCCGCAAGAAAACTATCCCGACCGCCTTACTGCCGCCTGTTTTGACCGCTGGGAT 3720

1221 [L](javascript:L(1221)) [G](javascript:G(1222)) [P](javascript:P(1223)) [Q](javascript:Q(1224)) [E](javascript:E(1225)) [N](javascript:N(1226)) [Y](javascript:Y(1227)) [P](javascript:P(1228)) [D](javascript:D(1229)) [R](javascript:R(1230)) [L](javascript:L(1231)) [T](javascript:T(1232)) [A](javascript:A(1233)) [A](javascript:A(1234)) [C](javascript:C(1235)) [F](javascript:F(1236)) [D](javascript:D(1237)) [R](javascript:R(1238)) [W](javascript:W(1239)) [D](javascript:D(1240)) 1240

3721 CTGCCATTGTCAGACATGTATACCCCGTACGTCTTCCCGAGCGAAAACGGTCTGCGCTGC 3780

1241 [L](javascript:L(1241)) [P](javascript:P(1242)) [L](javascript:L(1243)) [S](javascript:S(1244)) [D](javascript:D(1245)) [M](javascript:M(1246)) [Y](javascript:Y(1247)) [T](javascript:T(1248)) [P](javascript:P(1249)) [Y](javascript:Y(1250)) [V](javascript:V(1251)) [F](javascript:F(1252)) [P](javascript:P(1253)) [S](javascript:S(1254)) [E](javascript:E(1255)) [N](javascript:N(1256)) [G](javascript:G(1257)) [L](javascript:L(1258)) [R](javascript:R(1259)) [C](javascript:C(1260)) 1260

3781 GGGACGCGCGAATTGAATTATGGCCCACACCAGTGGCGCGGCGACTTCCAGTTCAACATC 3840

1261 [G](javascript:G(1261)) [T](javascript:T(1262)) [R](javascript:R(1263)) [E](javascript:E(1264)) [L](javascript:L(1265)) [N](javascript:N(1266)) [Y](javascript:Y(1267)) [G](javascript:G(1268)) [P](javascript:P(1269)) [H](javascript:H(1270)) [Q](javascript:Q(1271)) [W](javascript:W(1272)) [R](javascript:R(1273)) [G](javascript:G(1274)) [D](javascript:D(1275)) [F](javascript:F(1276)) [Q](javascript:Q(1277)) [F](javascript:F(1278)) [N](javascript:N(1279)) [I](javascript:I(1280)) 1280

3841 AGCCGCTACAGTCAACAGCAACTGATGGAAACCAGCCATCGCCATCTGCTGCACGCGGAA 3900

1281 [S](javascript:S(1281)) [R](javascript:R(1282)) [Y](javascript:Y(1283)) [S](javascript:S(1284)) [Q](javascript:Q(1285)) [Q](javascript:Q(1286)) [Q](javascript:Q(1287)) [L](javascript:L(1288)) [M](javascript:M(1289)) [E](javascript:E(1290)) [T](javascript:T(1291)) [S](javascript:S(1292)) [H](javascript:H(1293)) [R](javascript:R(1294)) [H](javascript:H(1295)) [L](javascript:L(1296)) [L](javascript:L(1297)) [H](javascript:H(1298)) [A](javascript:A(1299)) [E](javascript:E(1300)) 1300

3901 GAAGGCACATGGCTGAATATCGACGGTTTCCATATGGGGATTGGTGGCGACGACTCCTGG 3960

1301 [E](javascript:E(1301)) [G](javascript:G(1302)) [T](javascript:T(1303)) [W](javascript:W(1304)) [L](javascript:L(1305)) [N](javascript:N(1306)) [I](javascript:I(1307)) [D](javascript:D(1308)) [G](javascript:G(1309)) [F](javascript:F(1310)) [H](javascript:H(1311)) [M](javascript:M(1312)) [G](javascript:G(1313)) [I](javascript:I(1314)) [G](javascript:G(1315)) [G](javascript:G(1316)) [D](javascript:D(1317)) [D](javascript:D(1318)) [S](javascript:S(1319)) [W](javascript:W(1320)) 1320

3961 AGCCCGTCAGTATCGGCGGAATTCCAGCTGAGCGCCGGTCGCTACCATTACCAGTTGGTC 4020

1321 [S](javascript:S(1321)) [P](javascript:P(1322)) [S](javascript:S(1323)) [V](javascript:V(1324)) [S](javascript:S(1325)) [A](javascript:A(1326)) [E](javascript:E(1327)) [F](javascript:F(1328)) [Q](javascript:Q(1329)) [L](javascript:L(1330)) [S](javascript:S(1331)) [A](javascript:A(1332)) [G](javascript:G(1333)) [R](javascript:R(1334)) [Y](javascript:Y(1335)) [H](javascript:H(1336)) [Y](javascript:Y(1337)) [Q](javascript:Q(1338)) [L](javascript:L(1339)) [V](javascript:V(1340)) 1340

4021 TGGTGTCAGGGGATCCCCCGGGCTGCAGCCAATATGGGATCGGCCATTGAACAAGATGGA 4080

1341 [W](javascript:W(1341)) [C](javascript:C(1342)) [Q](javascript:Q(1343)) [G](javascript:G(1344)) [I](javascript:I(1345)) [P](javascript:P(1346)) [R](javascript:R(1347)) [A](javascript:A(1348)) [A](javascript:A(1349)) [A](javascript:A(1350)) [N](javascript:N(1351)) [M](javascript:M(1352)) [G](javascript:G(1353)) [S](javascript:S(1354)) [A](javascript:A(1355)) [I](javascript:I(1356)) [E](javascript:E(1357)) [Q](javascript:Q(1358)) [D](javascript:D(1359)) [G](javascript:G(1360)) 1360

4081 TTGCACGCAGGTTCTCCGGCCGCTTGGGTGGAGAGGCTATTCGGCTATGACTGGGCACAA 4140

1361 [L](javascript:L(1361)) [H](javascript:H(1362)) [A](javascript:A(1363)) [G](javascript:G(1364)) [S](javascript:S(1365)) [P](javascript:P(1366)) [A](javascript:A(1367)) [A](javascript:A(1368)) [W](javascript:W(1369)) [V](javascript:V(1370)) [E](javascript:E(1371)) [R](javascript:R(1372)) [L](javascript:L(1373)) [F](javascript:F(1374)) [G](javascript:G(1375)) [Y](javascript:Y(1376)) [D](javascript:D(1377)) [W](javascript:W(1378)) [A](javascript:A(1379)) [Q](javascript:Q(1380)) 1380

4141 CAGACAATCGGCTGCTCTGATGCCGCCGTGTTCCGGCTGTCAGCGCAGGGGCGCCCGGTT 4200

1381 [Q](javascript:Q(1381)) [T](javascript:T(1382)) [I](javascript:I(1383)) [G](javascript:G(1384)) [C](javascript:C(1385)) [S](javascript:S(1386)) [D](javascript:D(1387)) [A](javascript:A(1388)) [A](javascript:A(1389)) [V](javascript:V(1390)) [F](javascript:F(1391)) [R](javascript:R(1392)) [L](javascript:L(1393)) [S](javascript:S(1394)) [A](javascript:A(1395)) [Q](javascript:Q(1396)) [G](javascript:G(1397)) [R](javascript:R(1398)) [P](javascript:P(1399)) [V](javascript:V(1400)) 1400

4201 CTTTTTGTCAAGACCGACCTGTCCGGTGCCCTGAATGAACTGCAGGACGAGGCAGCGCGG 4260

1401 [L](javascript:L(1401)) [F](javascript:F(1402)) [V](javascript:V(1403)) [K](javascript:K(1404)) [T](javascript:T(1405)) [D](javascript:D(1406)) [L](javascript:L(1407)) [S](javascript:S(1408)) [G](javascript:G(1409)) [A](javascript:A(1410)) [L](javascript:L(1411)) [N](javascript:N(1412)) [E](javascript:E(1413)) [L](javascript:L(1414)) [Q](javascript:Q(1415)) [D](javascript:D(1416)) [E](javascript:E(1417)) [A](javascript:A(1418)) [A](javascript:A(1419)) [R](javascript:R(1420)) 1420

4261 CTATCGTGGCTGGCCACGACGGGCGTTCCTTGCGCAGCTGTGCTCGACGTTGTCACTGAA 4320

1421 [L](javascript:L(1421)) [S](javascript:S(1422)) [W](javascript:W(1423)) [L](javascript:L(1424)) [A](javascript:A(1425)) [T](javascript:T(1426)) [T](javascript:T(1427)) [G](javascript:G(1428)) [V](javascript:V(1429)) [P](javascript:P(1430)) [C](javascript:C(1431)) [A](javascript:A(1432)) [A](javascript:A(1433)) [V](javascript:V(1434)) [L](javascript:L(1435)) [D](javascript:D(1436)) [V](javascript:V(1437)) [V](javascript:V(1438)) [T](javascript:T(1439)) [E](javascript:E(1440)) 1440

**Primer KO-*neo*-F1**

4321 GCGGGAAGGGACTGGCTGCTATTGGGCGAAGTGCCGGGGCAGGATCTCCTGTCATCTCAC 4380

1441 [A](javascript:A(1441)) [G](javascript:G(1442)) [R](javascript:R(1443)) [D](javascript:D(1444)) [W](javascript:W(1445)) [L](javascript:L(1446)) [L](javascript:L(1447)) [L](javascript:L(1448)) [G](javascript:G(1449)) [E](javascript:E(1450)) [V](javascript:V(1451)) [P](javascript:P(1452)) [G](javascript:G(1453)) [Q](javascript:Q(1454)) [D](javascript:D(1455)) [L](javascript:L(1456)) [L](javascript:L(1457)) [S](javascript:S(1458)) [S](javascript:S(1459)) [H](javascript:H(1460)) 1460

4381 CTTGCTCCTGCCGAGAAAGTATCCATCATGGCTGATGCAATGCGGCGGCTGCATACGCTT 4440

1461 [L](javascript:L(1461)) [A](javascript:A(1462)) [P](javascript:P(1463)) [A](javascript:A(1464)) [E](javascript:E(1465)) [K](javascript:K(1466)) [V](javascript:V(1467)) [S](javascript:S(1468)) [I](javascript:I(1469)) [M](javascript:M(1470)) [A](javascript:A(1471)) [D](javascript:D(1472)) [A](javascript:A(1473)) [M](javascript:M(1474)) [R](javascript:R(1475)) [R](javascript:R(1476)) [L](javascript:L(1477)) [H](javascript:H(1478)) [T](javascript:T(1479)) [L](javascript:L(1480)) 1480

**Primer KO-*neo*-F2**

4441 GTCCGGCTACCTGCCCATTCGACCACCAAGCGAAACATCGCATCGAGCGAGCACGTACT 4500

1481 [D](javascript:D(1481)) [P](javascript:P(1482)) [A](javascript:A(1483)) [T](javascript:T(1484)) [C](javascript:C(1485)) [P](javascript:P(1486)) [F](javascript:F(1487)) [D](javascript:D(1488)) [H](javascript:H(1489)) [Q](javascript:Q(1490)) [A](javascript:A(1491)) [K](javascript:K(1492)) [H](javascript:H(1493)) [R](javascript:R(1494)) [I](javascript:I(1495)) [E](javascript:E(1496)) [R](javascript:R(1497)) [A](javascript:A(1498)) [R](javascript:R(1499)) [T](javascript:T(1500)) 1500

4501 CGGATGGAAGCCGGTCTTGTCGATCAGGATGATCTGGACGAAGAGCATCAGGGGCTCGCG 4560

1501 [R](javascript:R(1501)) [M](javascript:M(1502)) [E](javascript:E(1503)) [A](javascript:A(1504)) [G](javascript:G(1505)) [L](javascript:L(1506)) [V](javascript:V(1507)) [D](javascript:D(1508)) [Q](javascript:Q(1509)) [D](javascript:D(1510)) [D](javascript:D(1511)) [L](javascript:L(1512)) [D](javascript:D(1513)) [E](javascript:E(1514)) [E](javascript:E(1515)) [H](javascript:H(1516)) [Q](javascript:Q(1517)) [G](javascript:G(1518)) [L](javascript:L(1519)) [A](javascript:A(1520)) 1520

4561 CCAGCCGAACTGTTCGCCAGGCTCAAGGCGCGCATGCCCGACGGCGAGGATCTCGTCGTG 4620

1521 [P](javascript:P(1521)) [A](javascript:A(1522)) [E](javascript:E(1523)) [L](javascript:L(1524)) [F](javascript:F(1525)) [A](javascript:A(1526)) [R](javascript:R(1527)) [L](javascript:L(1528)) [K](javascript:K(1529)) [A](javascript:A(1530)) [R](javascript:R(1531)) [M](javascript:M(1532)) [P](javascript:P(1533)) [D](javascript:D(1534)) [G](javascript:G(1535)) [E](javascript:E(1536)) [D](javascript:D(1537)) [L](javascript:L(1538)) [V](javascript:V(1539)) [V](javascript:V(1540)) 1540

4621 ACCCATGGCGATGCCTGCTTGCCGAATATCATGGTGGAAAATGGCCGCTTTTCTGGATTC 4680

1541 [T](javascript:T(1541)) [H](javascript:H(1542)) [G](javascript:G(1543)) [D](javascript:D(1544)) [A](javascript:A(1545)) [C](javascript:C(1546)) [L](javascript:L(1547)) [P](javascript:P(1548)) [N](javascript:N(1549)) [I](javascript:I(1550)) [M](javascript:M(1551)) [V](javascript:V(1552)) [E](javascript:E(1553)) [N](javascript:N(1554)) [G](javascript:G(1555)) [R](javascript:R(1556)) [F](javascript:F(1557)) [S](javascript:S(1558)) [G](javascript:G(1559)) [F](javascript:F(1560)) 1560

**Primer KO-*neo*-R2**

4681 ATCGACTGTGGCCGGCTGGGTGTGGCGGACCGCTATCAGGACATAGCGTTGGCTACCCGT 4740

1561 [I](javascript:I(1561)) [D](javascript:D(1562)) [C](javascript:C(1563)) [G](javascript:G(1564)) [R](javascript:R(1565)) [L](javascript:L(1566)) [G](javascript:G(1567)) [V](javascript:V(1568)) [A](javascript:A(1569)) [D](javascript:D(1570)) [R](javascript:R(1571)) [Y](javascript:Y(1572)) [Q](javascript:Q(1573)) [D](javascript:D(1574)) [I](javascript:I(1575)) [A](javascript:A(1576)) [L](javascript:L(1577)) [A](javascript:A(1578)) [T](javascript:T(1579)) [R](javascript:R(1580)) 1580

4741 GATATTGCTGAAGAGCTTGGCGGCGAATGGGCTGACCGCTTCCTCGTGCTTTACGGTATC 4800

1581 [D](javascript:D(1581)) [I](javascript:I(1582)) [A](javascript:A(1583)) [E](javascript:E(1584)) [E](javascript:E(1585)) [L](javascript:L(1586)) [G](javascript:G(1587)) [G](javascript:G(1588)) [E](javascript:E(1589)) [W](javascript:W(1590)) [A](javascript:A(1591)) [D](javascript:D(1592)) [R](javascript:R(1593)) [F](javascript:F(1594)) [L](javascript:L(1595)) [V](javascript:V(1596)) [L](javascript:L(1597)) [Y](javascript:Y(1598)) [G](javascript:G(1599)) [I](javascript:I(1600)) 1600

**Primer KO-*neo*-R1**

4801 GCCGCTCCCGATTCGCAGCGCATCGCCTTCTATCGCCTTCTTGACGAGTTCTTCTGA 4857

1601 [A](javascript:A(1601)) [A](javascript:A(1602)) [P](javascript:P(1603)) [D](javascript:D(1604)) [S](javascript:S(1605)) [Q](javascript:Q(1606)) [R](javascript:R(1607)) [I](javascript:I(1608)) [A](javascript:A(1609)) [F](javascript:F(1610)) [Y](javascript:Y(1611)) [R](javascript:R(1612)) [L](javascript:L(1613)) [L](javascript:L(1614)) [D](javascript:D(1615)) [E](javascript:E(1616)) [F](javascript:F(1617)) [F](javascript:F(1618)) [*](javascript:Z(1619)) 1619

**Supporting References**

1. Tybulewicz VL, Crawford CE, Jackson PK, Bronson RT, Mulligan RC (1991) Neonatal lethality and lymphopenia in mice with a homozygous disruption of the c-abl proto-oncogene. Cell 65: 1153-1163.

2. Sambrook J, Fritsch EF, Maniatis T (1989) Molecular cloning-A laboratory manual; Sambrook J, Fritsch EF, Maniatis T, editors. New York: Cold Spring Harbor Laboratory.

3. Feinberg AP, Vogelstein B (1983) A technique for radiolabeling DNA restriction endonuclease fragments to high specific activity. Anal Biochem 132: 6-13.

4. Rozen S, Skaletsky H (2000) Primer3 on the WWW for general users and for biologist programmers. Methods Mol Biol 132: 365-386.

5. Chomczynski P, Sacchi N (1987) Single-step method of RNA isolation by acid guanidinium thiocyanate-phenol-chloroform extraction. Anal Biochem 162: 156-159.

6. Bonasera V, Alberti S, A.c. S (2007) Protocol for high-sensitivity/long linear-range spectrofluorimetric DNA quantification using ethidium bromide. BioTechniques 43: 173-176.

7. Devereux J, Haeberli P, Smithies O (1984) A comprehensive set of sequence analysis programs for the VAX. Nucleic Acids Res 12: 387-395.

8. Thomas KR, Capecchi MR (1987) Site-directed mutagenesis by gene targeting in mouse embryo-derived stem cells. Cell 51: 503-512.

9. Zanna P, Trerotola M, Vacca G, Bonasera V, Palombo B, et al. (2007) Trop-1 is a novel cell growth stimulatory molecule that marks early stages of tumor progression. Cancer 110: 452-464.

10. Bergsagel PL, Korin CV, Timblin CR, Trepel J, Kuehl WM (1992) A murine cDNA encodes a pan-epithelial glycoprotein that is also expressed on plasma cells. J Immunol 148: 590-596.
