# Supplementary material for: mTrop1/Epcam Knockout Mice Develop Congenital Tufting Enteropathy through Dysregulation of Intestinal E-cadherin/β-catenin
Source: PLoS One. 2012 Nov 28;7(11):e49302. doi: 10.1371/journal.pone.0049302 (PMC3509129; doi:10.1371/journal.pone.0049302)
Supplement: Table S1 — Primer sequences. (DOC) [file pone.0049302.s002.doc]

**Table S1. Primer sequences**

| ***mTrop1* replacement vector; 5’ homology arm** |
| --- |
| X(FOR): 5’-GTTAATGAGCCTCATAAAGA-3’  VIII(FOR): 5’-TAACCCCCCTCCCTGATTAC-3’  VI (FOR): 5’-ATGACTCTAGTGGTTTTAAC-3’  IV (FOR): 5’-TCCTCTTCCTCCTCCTCCTT-3’  II(FOR): 5’-AAGAATCATAAGAAGCAGA-3’  mT1-3-I(FOR): 5’-CTGAAGGATTGTGGGAAGGAA-3’  XI(FOR): 5’-ACTGCTCTTTTGCCATCACC-3’  I(FOR): 5’-GACTCCACCCCCACAGTTAC-3’  XII(FOR): 5’-CCCGACACCTCACCACACCC-3’  XII-KpnI(FOR): 5’-GCGggtaccCCCGACACCTCACCACACCC-3’  IX(REV): 5’-ATCTACTTGTATAATTTAAC-3’  VII(REV): 5’-GCTACACAGAGAAACCCTGT-3’  V(REV): 5’-TTAGAATTCTAGCACTGAAC-3’  III(REV): 5’- TCCTGGTAACCTTCCTACCT-3’  I(REV): 5’-GTAACTGTGGGGGTGGAGTC-3’  XII(REV): 5’-GGGTGTGGTGAGGTGTCGGG-3’  XIII REV): 5’-GCATCTCAAGGAGAATAGCC-3  XIII-BamHI(REV) 5’-GCGggatccGCATCTCAAGGAGAATAGCC-3’  mT1-3-XIII(REV) 5’-GCCCACCCCCACTAGTTAAT-3’  XIV(REV) 5’-TCAGATCAGCAGGGTGAGGG-3’ |
| ***mTrop1* replacement vector; 3’ homology arm** |
| XV(FOR) 5’-CCCGGTCCCCGGATGTGGGC-3’  mT1-3-XV(FOR) 5’-CGGAGGTGTGTTTTTCCTTT-3’  XVI(FOR) 5’-GGAAGTTTTCTACTTTAGGC-3’  mT1-3-XVI(FOR) 5’-AACTGGGTTTGCATGAAGAAG-3’  mT1-3-XVI-SalI(FOR) 5’-GCGgtcgacAACTGGGTTTGCATGAAGAAG-3’  mT1-3-XX(FOR) 5’-GAGGGCTGTAGTGAGCTTGG-3’  mT1-3-XIX(REV) 5’-GGGCCTTGGGACTCTAACTC-3’  mT1-3-XVII(REV) 5’-AGAGCCCCAGCTTCTCTACC-3’  mT1-3-XVIIbis(REV) 5’-AGTGAACCCACAAGGAAACG-3’  mT1-3-XVIIbis-NotI(REV) 5’-GCGgcggccgcCGTTTCCTTGTGGGTTCACT-3’  XVII(REV) 5’-GCTCTGATGGTCGTAGGGGC-3’  XVIII(REV) 5’-GGGTATTGTGTAAATGTTCT-3’ |
| **5’ Southern probes for targeted ES cell analysis** |
| KOmT1probe5-ext-F2(FOR) 5’-AAACTGGGCAATATGGGATTC-3’  KOmT1probe5-ext-R2(REV) 5’-GGCTCCCTTGTTCGTTCTTC-3’ |
| **3’ Southern probes for targeted ES cell analysis** |
| KOmT1probe3-ext-F1(FOR) 5’-TTCACCACAACGCTTACAGG-3’  KOmT1probe3-ext-R1(REV) 5’-GCCCATCGTGATGTGATCT-3’ |
| **Mouse genotyping and RT-PCR** |
| KO-*neo*-F1(FOR) 5’-aggatctcctgtcatctcaccttgctcctg-3’  KO-*neo*-R1(REV) 5’-aagaactcgtcaagaaggcgatagaaggcg-3’  KO-*neo*-F2(FOR) 5’-CATTCGACCACCAAGCGAAACATC-3’  KO-*neo*-R2(REV) 5’-ATATCACGGGTAGCCAACGCTATG-3’  m-*βglobin*-F1(FOR) 5’-ccaatctgctcacacaggatagagagggcagg-3’  m-*βglobin*-R1(REV) 5’-ccttgaggctgtccaagtgattcaggccatcg-3’  Bay-pGT1-R3(REV) 5’-TTCCCATGAATTCCAAGCTC-3’  mT1EX2-F2(FOR) 5’-TGAATATGGTGAATGCCAGTG-3’  Bay-pGT1-R4(REV) 5’-TCTAGGACAAGAGGGCGAGA-3’  mT1EX2-F1(FOR) 5’-TGGCAACAAGTTGCTCTCTG-3’  mT1EX3-R1(REV) 5’-GTCGTACAGCCCATCGTTGT-3’  mT1EX3-R2(REV) 5’-CTTGTCGGTTCTTCGGACTC-3’  mT1Int3-R1(REV) 5’-AAATATAACTGGATTAGGGCCAATAAAGCC-3’  β-gal-Baygen-R2(REV)5’-GTGATCCAGGACTGGGAAGA-3’  β-gal-Baygen-R3(REV)5’-GCGATCTGCGTTCTTCTTCT-3’  Bay-pGT1-R1bis(REV)5’-ACTCCAACCTCCGCAAACTC-3’  pGT1-βGEO-R1(REV) 5’-GACAGTATCGGCCTCAGGAAGATC-3’  pGT1-βGEO-R3 5’-TGCAGCCCGCAGATCTGAGAAGGTTCC-3’ |
