# Supplementary material for: mTrop1/Epcam Knockout Mice Develop Congenital Tufting Enteropathy through Dysregulation of Intestinal E-cadherin/β-catenin
Source: PLoS One. 2012 Nov 28;7(11):e49302. doi: 10.1371/journal.pone.0049302 (PMC3509129; doi:10.1371/journal.pone.0049302)
Supplement: Table S2 — Genotype frequencies in litters from heterozygous mTrop1 +/−× mTrop1 +/− crossings. (DOC) [file pone.0049302.s003.doc]

**Table S2. Genotype frequencies in litters from heterozygous *mTrop1*+/– x *mTrop1*+/– crossings.**

| **PUP AGE** | **LITTER** | **PUPS** |  | **GENOTYPE** |  |
| --- | --- | --- | --- | --- | --- |
|  |  |  | **+/+** | **+/-** | **-/-** |
|  | 1 | 9 | 2 | 6 | 1 |
|  | 2 | 11 | 3 | 6 | 2 |
|  | 3 | 10 | 3 | 4 | 3 |
|  | 4 | 6 | 1 | 3 | 2 |
| **At birth** | 5 | 9 | 2 | 3 | 4 |
|  | 6 | 8 | 2 | 2 | 4 |
|  | 7 | 8 | 1 | 4 | 3 |
|  | 8 | 4 | 0 | 3 | 1 |
|  | 9 | 10 | 4 | 5 | 1 |
|  | 10 | 8 | 3 | 4 | 1 |
|  | **Total** | **83** | **21** | **40** | **22** |
|  | 1 | 7 | 3 | 4 | 0 |
|  | 2 | 6 | 2 | 4 | 0 |
| **4 weeks** | 3 | 8 | 3 | 5 | 0 |
|  | 4 | 3 | 2 | 1 | 0 |
|  | **Total** | **24** | **10** | **14** | **0** |

21 (+/+) : 40 (+/-) : 22 (-/-) *vs* 1:2:1 (monogenic Mendelian inheritance): χ^2^ = 1.59 (H_0_ rejection for 2 degrees of freedom: χ^2^ ≥ 5.99).
