# Supplementary figures and images for: mTrop1/Epcam Knockout Mice Develop Congenital Tufting Enteropathy through Dysregulation of Intestinal E-cadherin/β-catenin
Source: PLoS One. 2012 Nov 28;7(11):e49302. doi: 10.1371/journal.pone.0049302 (PMC3509129; doi:10.1371/journal.pone.0049302)

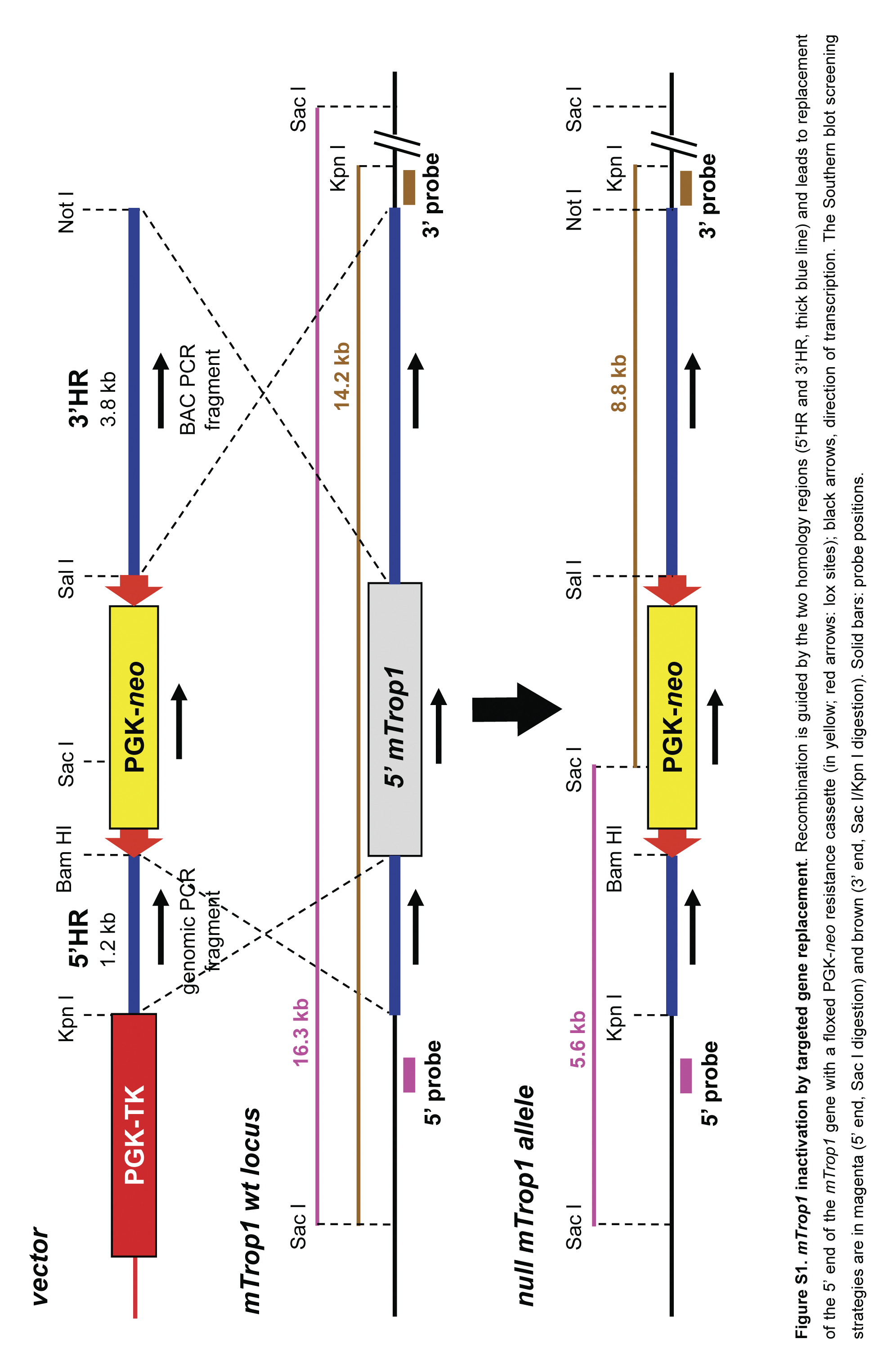

Supplement: Figure S1 — mTrop1 inactivation by targeted gene replacement. (TIF) [file pone.0049302.s004.tif]

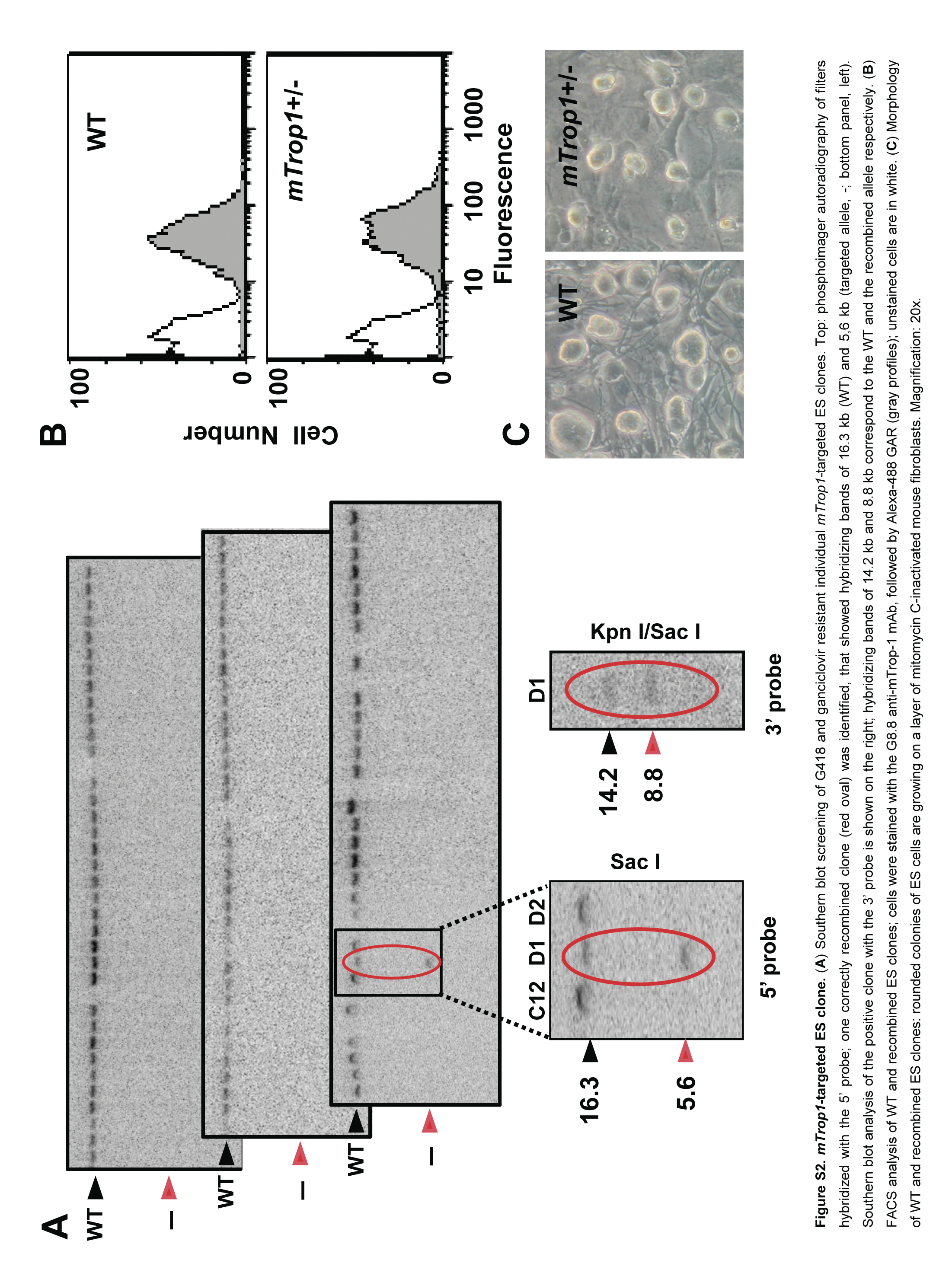

Supplement: Figure S2 — mTrop1 -targeted ES clone. (TIF) [file pone.0049302.s005.tif]

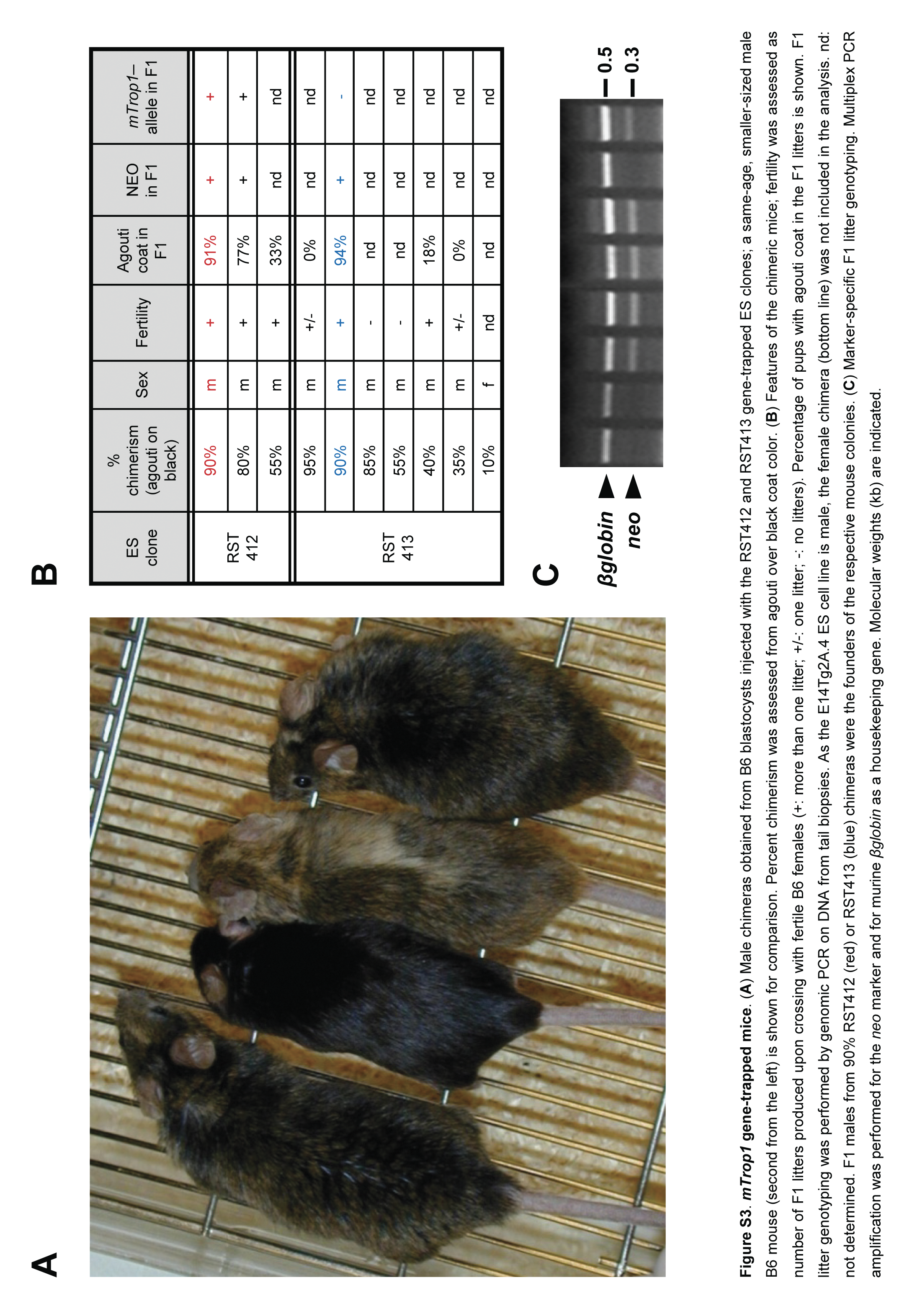

Supplement: Figure S3 — mTrop1 gene-trapped mice. (TIF) [file pone.0049302.s006.tif]

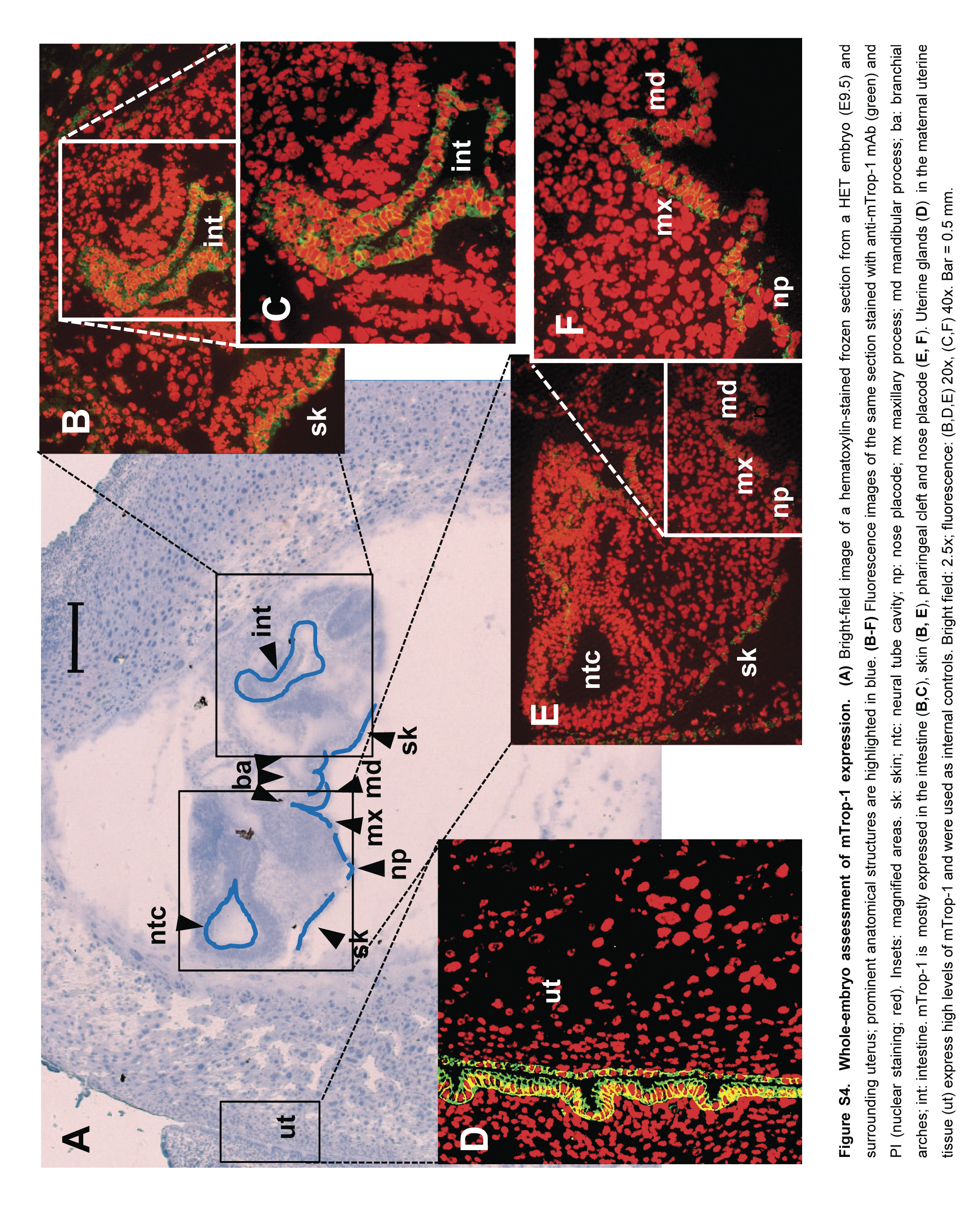

Supplement: Figure S4 — Whole-embryo assessment of mTrop-1 expression. (TIF) [file pone.0049302.s007.tif]

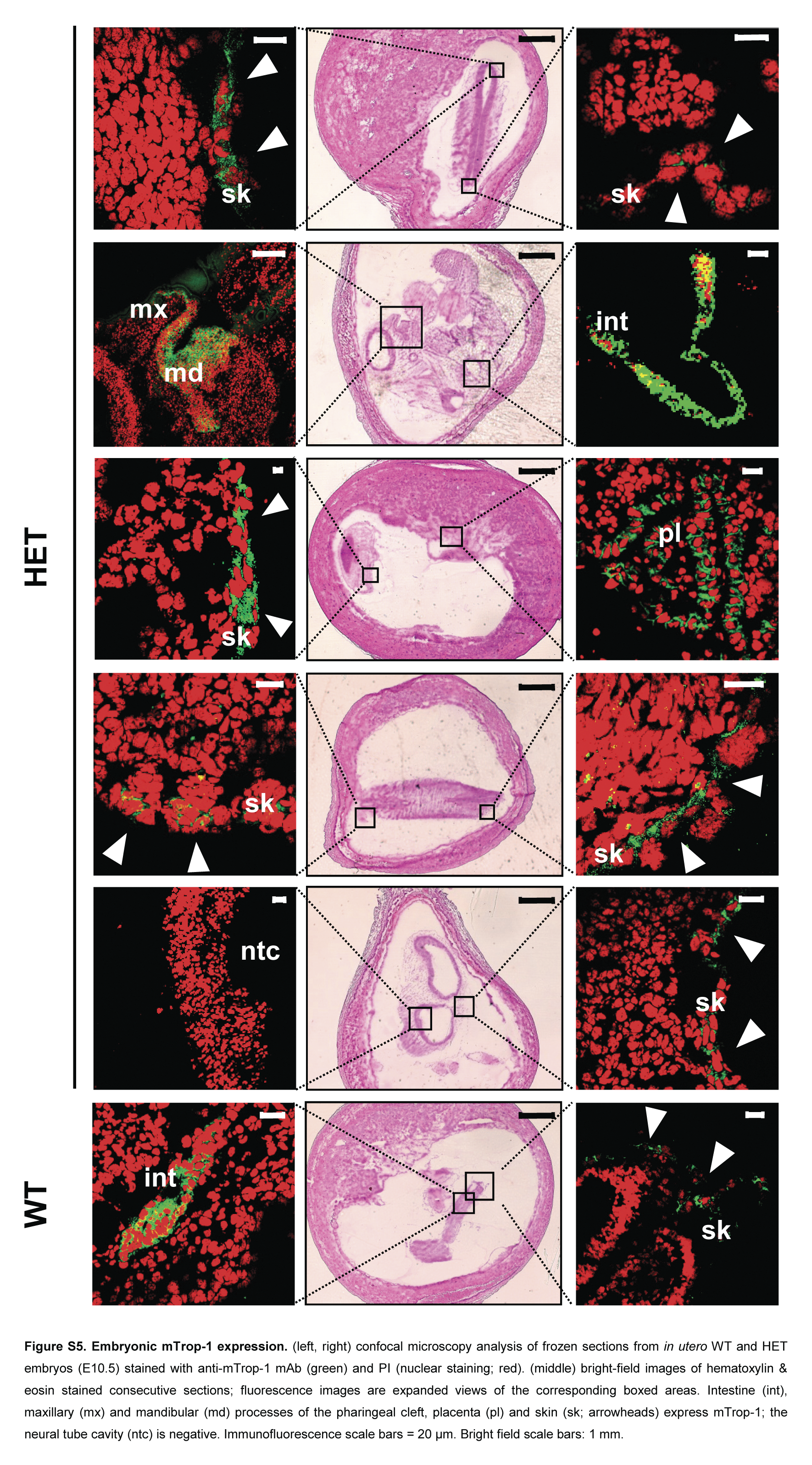

Supplement: Figure S5 — Embryonic mTrop-1 expression. (TIF) [file pone.0049302.s008.tif]

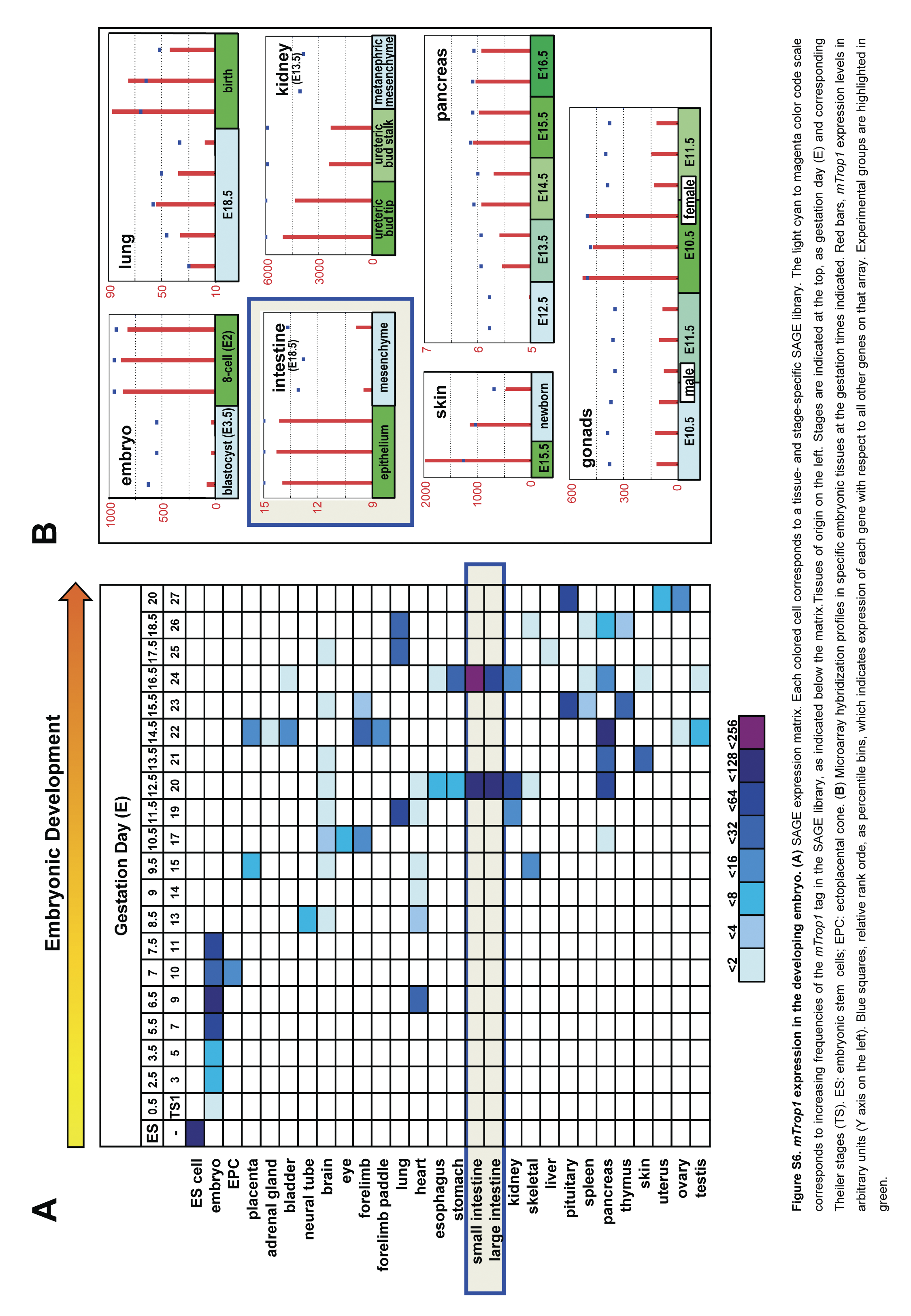

Supplement: Figure S6 — mTrop1 expression in the developing embryo. (TIF) [file pone.0049302.s009.tif]

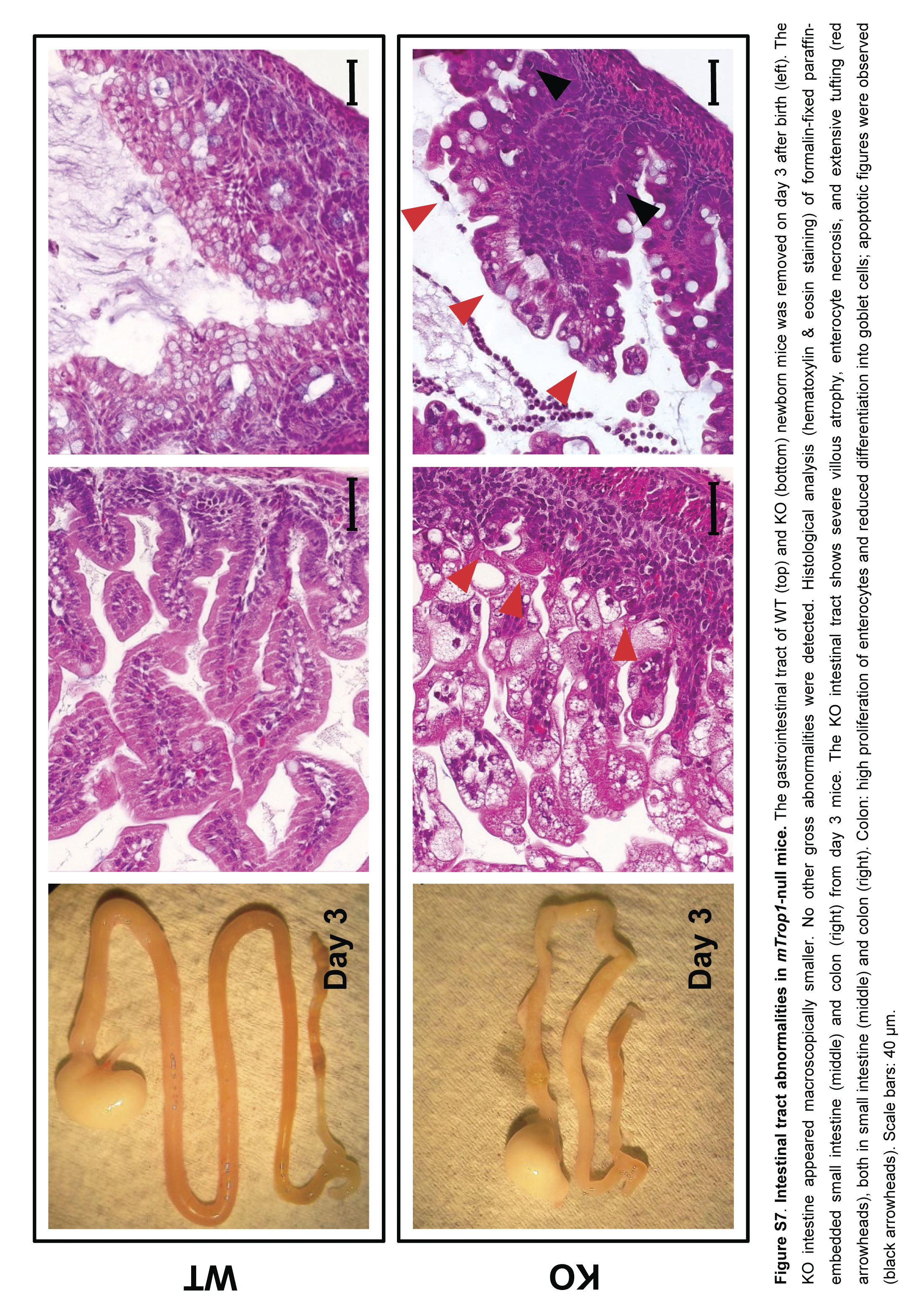

Supplement: Figure S7 — Intestinal tract abnormalities in mTrop1 -null mice. (TIF) [file pone.0049302.s010.tif]

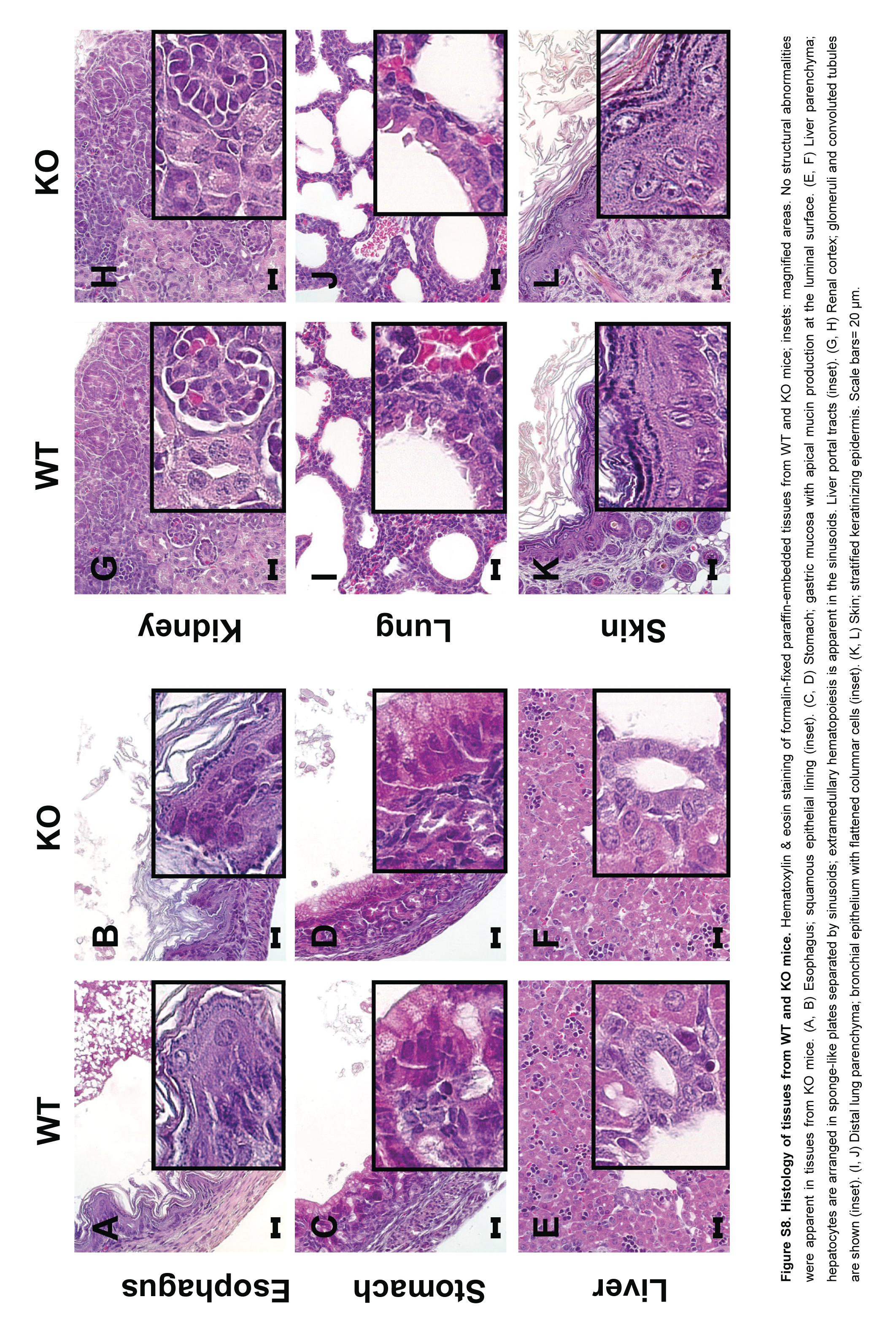

Supplement: Figure S8 — Histology of tissues from WT and KO mice. (TIF) [file pone.0049302.s011.tif]
